# Hydrological regimes and drainage systems of aerial rivers across South America

**DOI:** 10.1038/s41467-026-76303-y

**Published:** 2026-08-03

**Authors:** Wei Weng, Ping Fu, Ho Tin Hung, Kai-Chih Tseng, Li-Pen Wang, Yun-Man Hsu, Min-Hui Lo, Luís Costa, Vincent Dubreuil

**Affiliations:** 1 Department of Geography, National Taiwan University, Taipei, Taiwan, ROC; 2 Department of Civil Engineering, National Taiwan University, Taipei, Taiwan, ROC; 3 Department of Atmospheric Sciences, National Taiwan University, Taipei, Taiwan, ROC; 4 World Benchmarking Alliance, Amsterdam, The Netherlands; 5 Potsdam Institute for Climate Impact Research, Potsdam, Germany; 6 CNRS, UMR 6554 LETG, Université Rennes 2, Rennes, France

**Keywords:** Water resources, Governance, Element cycles, Hydrology, Environmental impact

## Abstract

Aerial rivers, long-term preferential pathways of atmospheric moisture flows, sustain ecosystems and regional hydrology. Maximizing their downwind discharge potential remains elusive without an objective way to identify critical upwind source regions. Here we analyze the drainage patterns of aerial rivers across South America and develop a mathematical method to identify turning points in moisture drainage curves, beyond which the intensity of upwind moisture contribution declines sharply. This provides a criterion for objectively delineating critical upwind basins and reveals pronounced continental variation in their spatial extent. These patterns reflect distinct regimes within the continental aerial river system, enabling its classification into four major drainage types: headwater, drainage, outfall, and plain areas. The four types differ in their potential for atmospheric moisture management and in their susceptibility to ecological tipping points, with implications for refining forest conservation priorities. Together, these findings support more effective land-water planning and moisture governance under a changing climate.

## Abstract (web summary)

This study shows that atmospheric moisture transport across South America is organised into four distinct aerial-river drainage regimes and identifies the critical upwind basins that most strongly influence downwind rainfall.

## Introduction

Atmospheric moisture is an invisible yet vital water resource, and its strategic management could be key to future water sustainability. However, the dynamic nature of moisture flows complicates effective management. Short-term flows shift direction and intensity, such as land–sea breezes, which can reverse the direction of moisture transport to coastal cities within a single day^1^. Similarly, atmospheric rivers (ARs) transport large volumes of moisture over periods ranging from a few hours to typically 3–5 days^2,3^.

Beyond these short-lived phenomena, scientists have identified long-term moisture pathways known as aerial rivers^4^, or flying rivers^5^. These represent preferential pathways of moisture transport that persist over extended periods and traverse any given region. In contrast to other filamentary structures of atmospheric flow—such as low-level jets (LLJs), which are narrow, fast-moving currents in the lower troposphere governed normally by diurnal to seasonal cycles^6,7^, or ARs, which are transient corridors of concentrated water vapor lasting less than a day and spanning ~400–600 km^2,3^—aerial rivers operate on broader spatial and temporal scales. They are long-term statistical pathways of moisture transport that integrate dynamic variations in wind. They embody the cumulative influence of short-term processes such as land–sea breezes, LLJs, ARs, and other atmospheric events, capturing their net effect on moisture transport at climatological timescales. Unlike LLJs and ARs, which are episodic and regional, aerial rivers can encompass any region on Earth and are often described as climatology expressed through the persistent flow of atmospheric moisture over time^5,8,9^.

**Fig. 1:**
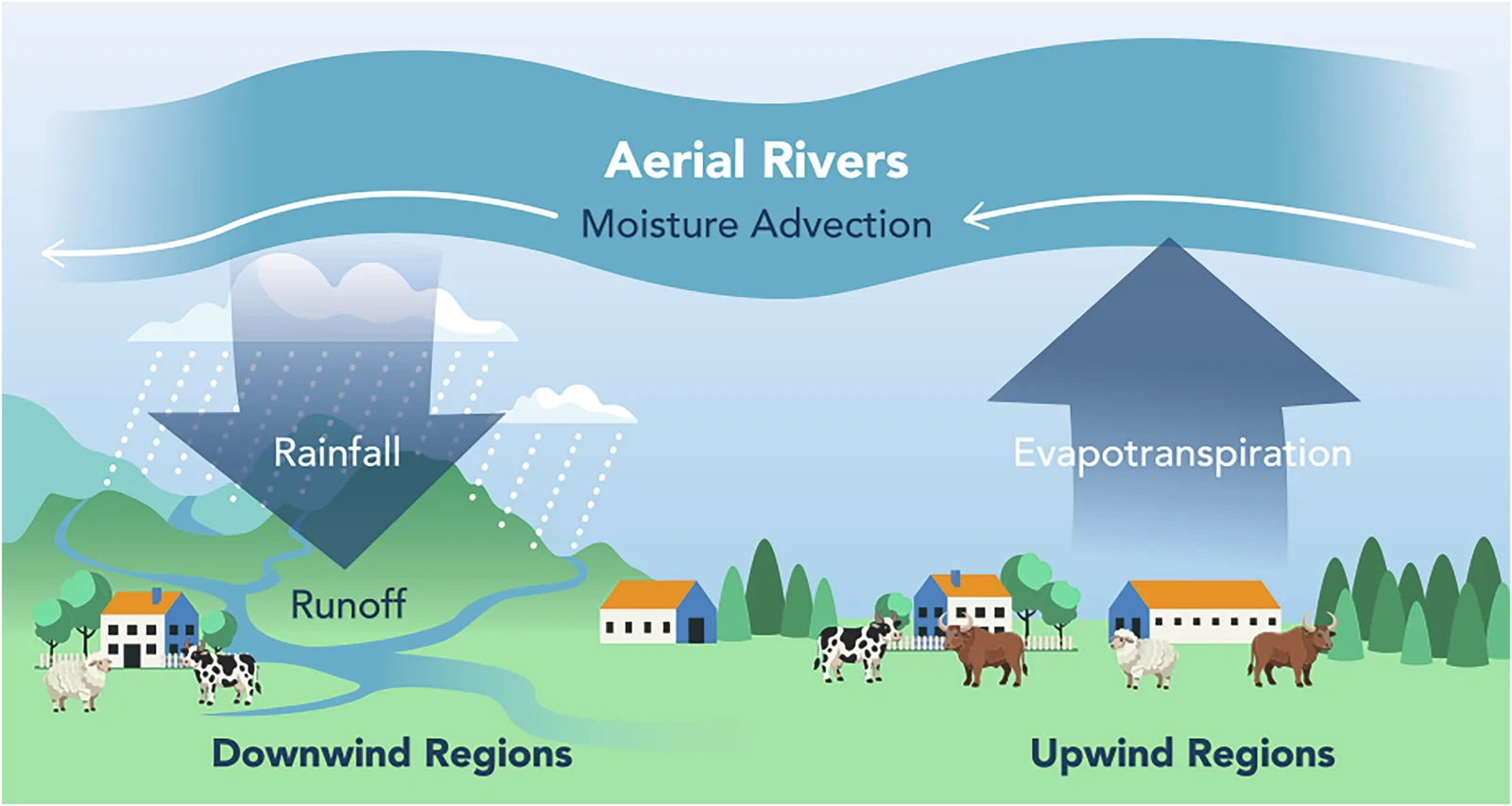
Conceptual diagram of aerial rivers. Aerial rivers are preferential pathways of moisture flow in the atmosphere. They are recharged by the evapotranspiration process in upwind regions and discharged by the precipitation process in downwind regions. Their integration into land-water management has traditionally been limited.

Aerial rivers shape hydrology worldwide. Opposite to the surface rivers, aerial rivers are recharged by the evapotranspiration process, routed by wind patterns, and discharged by precipitation processes (see Fig. 1). When flowing inland, land activities in upwind regions modify evapotranspiration, thereby influencing the moisture load of aerial rivers and their discharge in downwind areas^10^. These interactions are substantial from a management perspective^8,9,11^, as the significance of aerial rivers extends beyond sustaining downwind rainfall^9,12^ to regulating river runoff^8,13^ and total water storage^14^. For instance, research shows that deforestation in the upwind regions of Peruvian Ucayali can contribute to a 5–13% reduction in annual rainfall and a 19–50% decrease in annual runoff in the region^8^ via aerial rivers. This highlights the importance of incorporating aerial rivers into water management frameworks^11,15,16^. However, most existing water management institutions operate within catchment or political boundaries. Since aerial rivers transcend both topographic and administrative borders^17^, their integration into water management requires explicit accounting of upwind–downwind connections, particularly the identification of upwind source regions as management targets. Similar to surface water systems, where upstream land use determines downstream water quality and quantity and is therefore subject to management, upwind land use regulates the moisture entering aerial rivers and shapes downwind water availability^8,9,11–13,18^. Identifying upwind regions for targeted interventions, such as land conservation or land-use planning, is thus essential for effectively managing the water resources sustained by aerial rivers^11,15^, hereafter referred to as *aerial river management*.

Although advances in tracking atmospheric moisture flows have improved our ability to map upwind–downwind relationships^19–21^, delineating upwind regions for aerial river management remains a critical challenge. Given that aerial rivers are long-term prominent phenomena containing frequent changes of wind, the moisture provision from upwind regions follows a probabilistic manner^5^, leading to a hitched size-contribution^9,22^ characteristic of upwind moisture sources. In other words, point-to-point moisture provision is generally minimal or negligible. Conversely, tracing all regions that contribute moisture to a target area can result in the inclusion of extensive regions, many of which hold little practical relevance for management. For example, Keys et al.^23^ traced the moisture-providing regions for Pakistan and found that they extended from Scandinavia to Madagascar. To address this, a common approach for identifying upwind regions is to aggregate top-ranked moisture-contributing areas based on their level of significance to exclude regions with minimal relevance^8,24,25^. It is typically done by applying fixed thresholds predefined by researchers^8,9,25,26^. While some studies adopt a 40 percent threshold^8,24^, others use 70 percent^23^ or 80 percent^25^. The applicability of these conventional thresholds across regions remains to be assessed. This also underscores the need for a systematic approach to identify the most relevant upwind areas^8,26,27^.

One possible approach is to analyze the turning point of the moisture-drainage curve. Weng et al.^24^ presented such a curve for Santa Cruz de la Sierra, Bolivia. They indicated a turning point on the curve, beyond which the intensity of the city’s upwind moisture contributions declined sharply (Supplementary Fig. S1), but they did not specify how this turning point could be defined.

In this study, we explicitly examine the moisture drainage curve, which describes the relationship between the size and contribution of upwind moisture sources^23,24^ to a given region. We develop a mathematical method to systematically identify turning points where upwind moisture contribution intensity decreases sharply, enabling a broader assessment of their occurrence and variation across the continent. Using this approach, we further evaluate the justification of conventional one-size-fits-all thresholds employed in previous studies. We also examine regional differences in drainage conditions and how these reflect underlying continental aerial river patterns. For a given region, its upwind areas are variously referred to as source regions^24^, atmospheric basins^26^, or precipitationsheds^23,27^. In this paper, we adopt the term “atmospheric basins” for clarity. The portions of these basins identified as major contributors of moisture through the turning point analysis are referred to as “critical upwind basins.” Finally, we discuss the implications of the identified drainage patterns for water management and regional hydrology. Our analysis focuses on the South American continent, a hotspot for aerial river research^4,5,8,24,28,29^, allowing comparisons with existing studies. We concentrate on continental aerial rivers due to their relevance for water management through upwind land interventions. Accordingly, the drainage curves are derived from upwind contributions to terrestrial-sourced rainfall.

## Results

### Turning points reveal four major drainage types

**Fig. 2:**
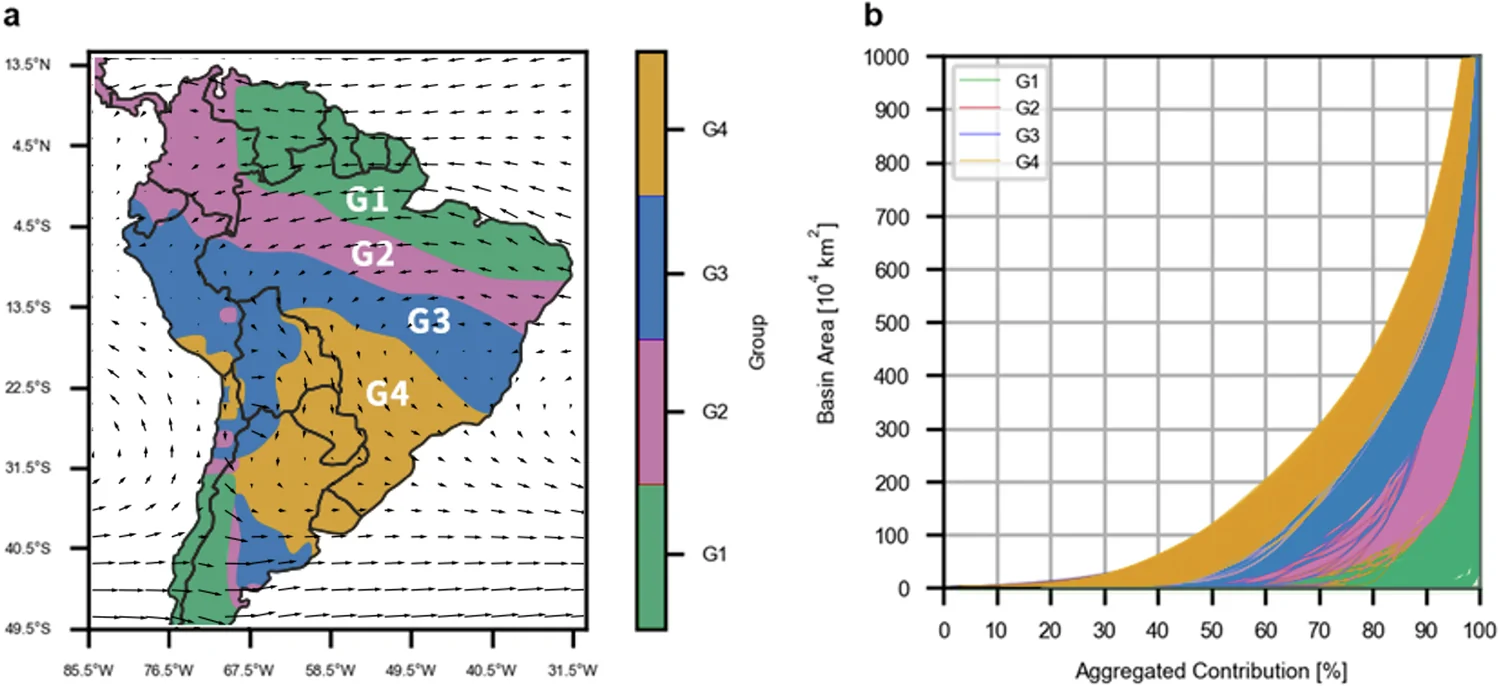
Spatial pattern of aerial river drainage types across South America. **a** Map of the four moisture drainage types (G1, G2, G3, and G4) **b** Moisture drainage curves across the South American continent and their classification into four clusters. Each drainage curve represents the relationship between upwind basin size and its contribution to terrestrial-sourced rainfall at the corresponding grid cell. G1 receives a high percentage of terrestrial moisture from a small upwind basin, whereas G4 requires a significantly larger upwind basin to reach the same contribution level compared to other types. In (**a**), the arrows represent the long-term average 843 hPa wind field (2006–2015) from ERA-Interim. Note that the division of the four types in (**a**) does not align with climatic or topographic boundaries.

**Fig. 3:**
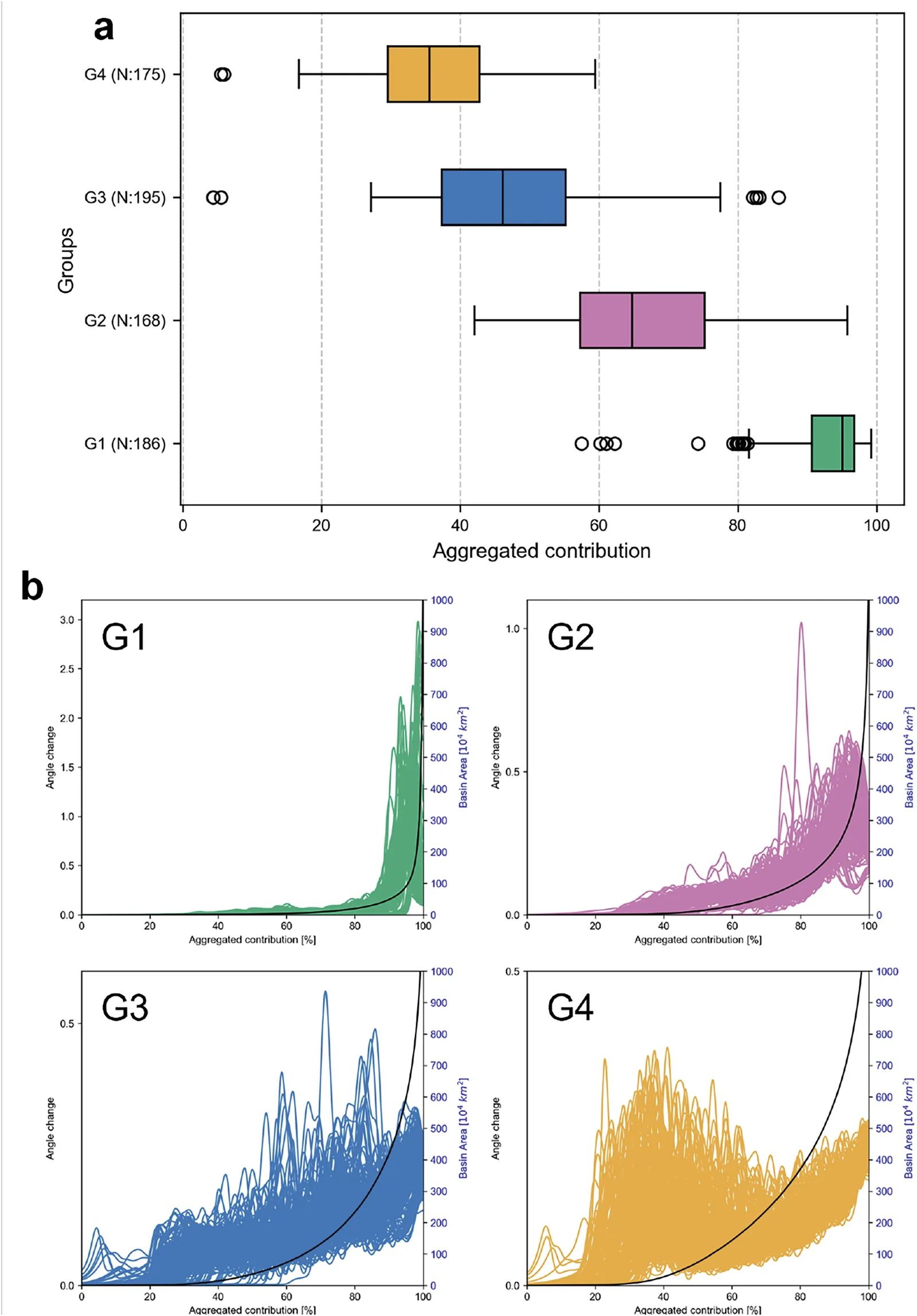
Identifying turning points through changes in curve angle. **a** Turning points of the four moisture drainage types. The turning point threshold varies across the four drainage types. The box shows the interquartile range, with the tick mark indicating the median value. The whiskers extend to the data points outside the middle 50%, and circles represent outliers, defined as data values located 1.5 times the interquartile range above the third quartile or below the first quartile. *N* represents the number of grid cells in each type. **b** First derivative of the curve angle for each type. The peak marks the turning point, indicating abrupt local changes in the curve’s angle. The black lines represent the median moisture drainage curve for each type, showing the relationship between basin size and aggregated contribution.

We analyzed moisture drainage curves for 724 grid cells (1.5° × 1.5°; see “Methods”) across the South American continent and observed significant variation in their shapes. A map of these 724 curves is available in Supplementary A. Using Calinski-Harabasz scores (see “Methods”), we identified four clusters, which were then classified using k-means. Figure 2 presents the classification results, showing both the different curve types and their spatial distribution. In addition to analyzing curve shapes, we examined the turning points for each moisture drainage type, as shown in Fig. 3.

The varying shapes of the moisture drainage curves indicate differences in moisture drainage conditions across the continent. As Fig. 2 shows, four distinct clusters of moisture drainage types are identified. The first type (G1, green) are areas where contribution from a small upwind basin dominates their terrestrial sourced rainfall. As Fig. 3b shows, the turning points for G1 occur at around 90–95% contribution, meaning that the critical upwind basin—defined in this study as the basin area before reaching the turning point—accounts for approximately 90–95% of terrestrial-sourced rainfall. G1 regions are primarily located in the windward Amazon, the Guiana Highlands, and much of Chile and Patagonia. The second type (G2, red) includes a west-east band extending from Colombia to the mid-Amazon and northeastern Brazil, as well as a small portion in northern Patagonia. G2’s critical upwind basin is less dominant in moisture contribution compared to G1 (see Fig. 3a), supplying around 60–75% of G2’s terrestrial-sourced rainfall. The third type (G3, blue) covers the southern Amazon, the Brazilian Highlands, the Pampas, and parts of the Andes. G3 is less efficient in the size-contribution relationship of its upwind basin, requiring a much larger area to achieve the same percentage of terrestrial-sourced rainfall as G1 and G2. The critical upwind basin in G3 contributes around 40–55% of terrestrial-sourced rainfall. The fourth type (G4, orange) includes the Chaco region, central-west Brazil, and the La Plata Basin. Upwind basin contribution per unit area in G4 is even lower than in G3. The turning points of G4 occur at around 30–40% of terrestrial-sourced rainfall.

### The characteristics of aerial rivers in each type

We employed Lagrangian specification of motion tracing to examine conditions of aerial rivers in each moisture drainage type. We analyzed the moisture traveling distance and time over the continent, the water content of the moisture flow, and the speed of the moisture flow as it reaches each type. The results are shown in Table 1. Generally, there are differences in moisture traveling time, speed, load, and delivery intensity between types, as indicated by the statistical difference tests in Table S1. Aerial river delivery intensity differs from the upwind moisture contribution intensity used for the drainage curve in that it represents the path-averaged linear density of moisture transport. We found that the aerial river travels the most to reach G4 regions, followed by G3 and G2; while G1 regions require the shortest travel distance from the ocean. It also takes the shortest time for the aerial river to reach G1, followed by G2, then G3, and the longest time to reach G4. The water content of the aerial river evolves as it delivers moisture to G1 and G2 with similar delivery intensity, but declines from G3 to G4. G2 receives the greatest moisture load from terrestrial aerial rivers. However, the moisture load decreases significantly when reaching G3. When the aerial river reaches G4, the moisture load is the smallest.

**Table 1:** Aerial river characteristics and difference tests across moisture drainage types

	Grid cell counts	Average continental recycling ratio	Water content [mm]	Moisture traveling time [h]	Moisture traveling distance [km]	Delivery intensity [mm km^−1^]	Wind speed at 850 hPa [m s^−1^]
G1	186	0.12	565.90	34.74	856.29	1.12*	7.49
G2	168	0.33	872.65	92.10	1831.30*	0.82*	4.81
G3	195	0.44	485.40*	156.27	2065.31*	0.31	3.22*
G4	175	0.49	429.88*	246.21	3657.95	0.14	3.48*

“*” indicates no significant difference (*P* ≥ 0.01) between values for the same variable. For detailed *P* values from the difference tests, please refer to Supplementary Table S1.

## Discussions

Here, we discuss the features of each moisture drainage type and their fluvial positions within aerial river systems, while also evaluating the potential and strategies for aerial river management for each type. In this study, aerial river management refers to land interventions, such as forest preservation or reforestation^15,18,24,30^, within critical upwind basins to secure or enhance the moisture load of aerial rivers, which ultimately precipitates in each moisture drainage type.

### G1 continental aerial river headwater regions

G1 represents regions where aerial rivers enter the South American continent from the oceans. G1 does not necessarily correspond to coastal areas, but rather to regions that serve as the primary entry points of moisture into the continent, such as central and southern Chile, which receive westerly moisture inflows from the Pacific^31,32^. Similar to surface watersheds, G1 regions are the headwater regions where the continental moisture transport originates. They feature an absolute dominance of continental-sourced rainfall from their critical upwind basins. This feature is evident in the sharp turning point at around 90–95% continental-sourced rainfall contribution in their moisture drainage curves. G1’s critical upwind basin is substantial, averaging 930,000 km². Additionally, G1 regions rely primarily on moisture directly sourced from the ocean, with continental-sourced rainfall contributing an average of 12% of total precipitation (see Table 1), of which the critical upwind basin optimally accounts for about 11%.

Critical upwind basin: dominating influence (averaged 90–95%, 930,000 km²).

Aerial river management potential: averaged 11% of total rainfall, due to oceanic dependency.

### G2 atmospheric drainage

G2 represents regions where atmospheric drainage increases while the dominance of the critical upwind basin over atmospheric drainage decreases. We found that as moisture flows from G1 into the continent’s interior (G2), the turning points of moisture drainage curves evolve within G2. They initially shift toward lower x-values, hinting at a lower contribution from G2’s critical upwind basins compared to G1 (see Supplementary Fig. S4a, b). At this stage, upwind moisture contribution intensity remains stable, but overall atmospheric drainage expands, and the dominance of critical upwind basins reduces. These areas mark the transition from G1 to G2, such as the northern part of Patagonia (illustrated by the Chubut example in Supplementary Fig. S4c). Subsequently, as aerial rivers travel further inland, the moisture drainage curves exhibit vertical shift toward higher y-values, reflecting a decline in per-area upwind contribution, or upwind moisture contribution intensity, along drainage expansion (see Supplementary Fig. S4a, b). This pattern is observed in regions such as the central Amazon and the northern part of the Magdalena River basin, where upwind moisture contribution intensity decreases as drainage grows (illustrated by the Valle del Cauca example in Supplementary Fig. S4c).

G2 is the region where aerial river delivery intensity begins to decline not yet significantly decreased compared to G1 (see Table 1). Meanwhile, atmospheric drainage continues to expand, making G2 the area that receives the largest amount of terrestrial-sourced moisture across the continent. The turning point for G2 occurs at approximately 60–75% of continental-sourced rainfall, yet its critical upwind basin is smaller than that of G1 (around 410,000 km²). As 33% of G2’s total rainfall depends on continental-sourced moisture (see Table 1), the critical upwind basin contributes approximately 20–25% of its total rainfall (including both terrestrial and oceanic sources).

Critical upwind basin: moderate influence (averaged 60–75%, 410,000 km^2^).

Aerial river management potential: averaged 20–25% of total rainfall.

### G3 aerial river outfall

The G3 type occurs where aerial rivers slow abruptly, resembling river outfall areas^5^. Although the travel distance from the ocean is comparable to G2, by the time the aerial river reaches G3, its moisture delivery intensity is markedly reduced, delivering far less moisture. The critical upwind basin for G3 is smaller than that of G2 (averaging 260,000 km²) and contributes only 40–55% to continental-sourced rainfall. Nevertheless, G3 regions rely more heavily on continental moisture sources for precipitation (see Table 1), which makes the management of aerial rivers in these regions especially relevant.

More importantly, implementing aerial river management in G3 can be strategic for surface water management as well. In particular, the Peruvian, Chilean, and Brazilian portions of G3 are located in the upstream regions of major river basins. Managing aerial rivers in these areas is especially valuable for integrated water management, as surface runoff can amplify the effects of aerial river interventions^24^.

Critical upwind basin: moderate influence (averaged 40–55%, 260,000 km^2^).

Aerial river management potential: averaged 18–24% of total rainfall (including both terrestrial and oceanic sources), strategic for water management through the interconnected dynamics of aerial rivers and surface rivers^24^, as most G3 areas are located in the upstream regions of major surface river basins.

### G4 atmospheric plain

G4 areas are located further downwind than G1, G2, and G3. Unlike surface river plains, G4 aerial river plains receive the least moisture. When aerial rivers reach G4, they have no significant changes in speed, but their moisture delivery intensity is lower than in G3, being the lowest across the continent. In G4, the critical upwind basins show the lowest dominance in continental moisture provision. Nevertheless, because G4 regions have the highest dependency on continental moisture instead of oceanic source, their critical upwind basins actually contribute the largest share of their total rainfall among the four drainage types. Thus, even though aerial rivers deliver moisture less efficiently further downwind, the role of critical upwind basins can be more relevant for water management in G4 regions, particularly in water-scarce cities and semi-arid regions. For instance, the Bolivian city of Santa Cruz de la Sierra, identified in the literature as having strong potential for aerial river management^24^, is situated in G4.

Critical upwind basin: moderate influence (averaged 30–40%, averaged 140,000 km^2^).

Aerial river management potential: averaged 15–20% of total rainfall (including both terrestrial and oceanic sources), as this area depends heavily on continental-sourced rainfall.

Atmospheric moisture follows specific climatological pathways, forming an aerial drainage system. For a given region, its critical upwind basin is the dominant moisture source that provides moisture more efficiently via aerial rivers than other areas within the drainage domain. Aerial rivers as statistical phenomena of long-term moisture transport combine the effects of seasonal cycles and interannual variability, as well as processes of local recycling, random diffusion, and large-scale advection. Among these contributing factors, certain mechanisms exert a stronger control on sustaining precipitation than others over extended periods. Within this context, the critical upwind basin captures the main corridor of probabilistic aerial rivers that maintain regional rainfall. Our results demonstrate that these dominant source areas can be systematically identified using the turning points of drainage curves.

The occurrence of curve turning points differs among the four main drainage types (G1, G2, G3, G4), each falling within characteristic ranges and reflecting variations in the size and relevance of their critical upwind basins. From G1 to G4 (upwind to downwind), critical upwind basins decrease in both size and dominance of moisture provision. This occurs because, as aerial rivers move inland, their moisture delivery becomes less intense (lower dominance) and is increasingly constrained by prevailing winds (smaller critical upwind basin area). A pronounced “fall” in aerial river delivery intensity occurs at the G2–G3 transition, where wind speed also declines (see Table 1). Since the travel distance of moisture between G2 and G3 is not substantially different (see Table 1), we interpret this plummet as analogous to river outfall regions.

Across South America, two major continental aerial river drainage systems can be identified, each progressing from upwind to downwind through the sequence of G1, G2, G3, and G4. The tropical system originating from the continental northeast extends from the continent’s northern coastline to Paraguay and southern Brazil. It is substantially larger than the temperate system, which originates from the continental southwest and spans from Patagonia to the La Plata Basin. These two systems generally align with prevailing wind regimes of the austral Hadley and Ferrel cells, while also capturing the influence of regional periodic moisture pathways such as ARs^31,32^, terrain-induced flows^33,34^, and LLJs^6^. A sharp “fall” of G2 and G3 transition occurs between 5°S and 13°S within the tropical system (see Fig. 2a), where moisture delivery intensity declines abruptly. A corresponding meridional “fall” appears in the Salado River Basin (See Fig. 2a), marking a collective slowdown of moisture flow that may be influenced by lee-waves in Patagonia^34^. Ultimately, these two systems converge in the La Plata Basin, where G4 regions of the tropical and temperate aerial rivers join.

Turning to the methodological framework, the mathematical turning points on moisture drainage curves mark the threshold where moisture contribution intensity declines sharply from upwind regions, offering an objective criterion for identifying critical upwind basins of a given region. The significant variation in turning point occurrence across the continent demonstrates that commonly used thresholds—such as 40%, 70%, or 80%^9,24,25^—for designating critical upwind regions are not universally applicable. An 80% threshold results in an excessively large upwind area for most types except G1, which is the type less relevant for continental moisture management. For G2, G3, and G4 areas, the 40% threshold is more applicable but does not always align with the turning-point-indicated threshold in most cases (see Supplementary Fig. S3).

**Fig. 4:**
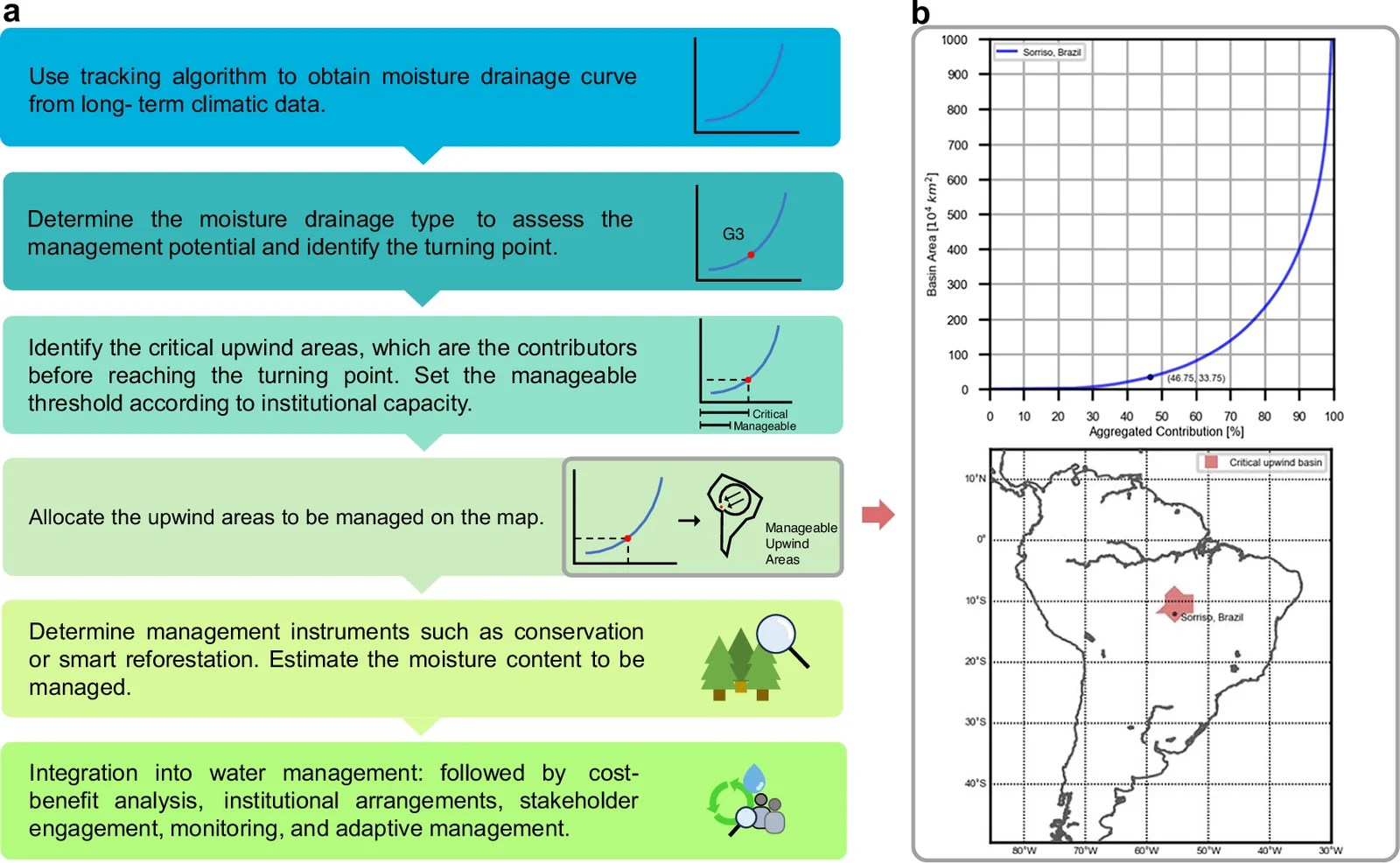
Framework and turning-point method for integrating critical upwind basins into water-resources management. **a** Framework for integrating aerial rivers into water management. **b** Identifying critical upwind basin with the moisture drainage curve and turning point: a case of Sorriso municipality.

**Fig. 5:**
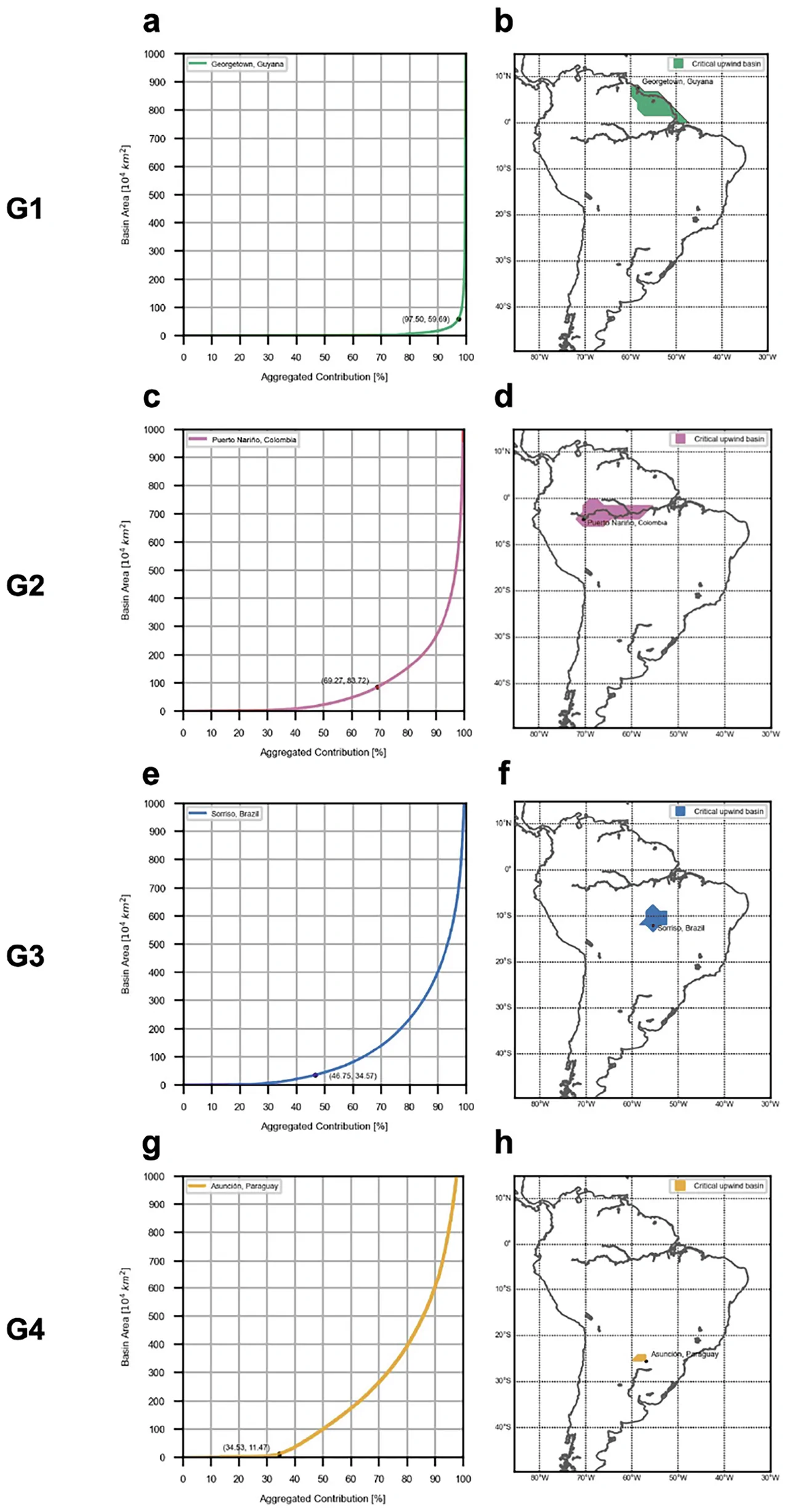
Example cases illustrating the identification of critical upwind basins. **a**, **c**, **e**, **g** Moisture drainage curves and turning points for selected regions (Georgetown, Puerto Nariño, Sorriso, and Asunción, respectively). **b**, **d**, **f**, **h** The corresponding maps of the critical upwind basins for each region.

Our study advances the understanding of aerial river characteristics and provides systematic methods for identifying turning points on moisture drainage curves, enabling the delineation of case-specific critical upwind basins. Moisture sustained by these basins via aerial rivers can be tracked and quantitatively integrated into broader water resource assessments. We develop a framework to assess the potential of aerial rivers and support their integration into water management (Fig. 4; see Supplementary C and Fig. 5 for example cases). First, we recommend obtaining the moisture drainage curve (see Method) and evaluating the aerial river management potential according to the moisture drainage type. G4 is the most effective region for aerial river management, followed by G3, then G2, with G1 being the least effective in terms of aerial river management per unit of critical upwind basin area. In regions with the potential to manage aerial rivers, identifying the turning points of moisture drainage curves can help determine case-specific critical upwind basins for targeted management—beyond which management efforts would be significantly less effective. For regions in South America, the moisture drainage type can be determined directly using the map in Fig. 2a, and the upwind basin size can be estimated based on the median value curve of each type (see Supplementary Fig. S5)—even without specialized modeling expertise. Critical upwind basins represent regions with the highest potential for effective aerial river management. However, the extent of these areas selected for management ultimately depends on the capacity of governing institutions, including their financial and political resources. In many cases, the critical upwind basins identified through turning points may not align with existing land management jurisdictions. As these basins often transcend administrative and topographic boundaries, their effective management requires institutional collaboration among entities responsible for regional governance^11,24^.

After accounting for management capacity, the critical upwind basin designated for management should be defined and mapped to guide decision-making (Figs. 4 and 5). Finally, appropriate management instruments, such as conservation^9,18,30^ or strategic reforestation^12,15,24^, should be selected and implemented, alongside an assessment of their potential impacts. For the atmospheric moisture component, moisture tracking tools can be applied to estimate the additional or preserved moisture delivered to the region via aerial rivers^12,21,24^. This should provide information for water planning that incorporates aerial rivers into broader cost–benefit analyses to define objectives, assess trade-offs, and set priorities^15^ (see Supplementary C for an example of critical upwind basin selection and the associated reforestation impact assessment). It should be complemented by institutional arrangements that define short- and long-term targets, clarify roles and coordination mechanisms, and establish supporting legal frameworks^11,35^. These arrangements should actively engage multiple stakeholders^35^ across affected regions to ensure inclusivity and effectiveness. Finally, adaptive management^36,37^ should be implemented to monitor outcomes, evaluate interventions, and revise plans in response to emerging uncertainties, feedback, and changing conditions.

The aerial rivers and their basin management challenge the current water management thinking. Managing aerial rivers at the continental scale necessitates a reconceptualization of the governable dimensions of hydrology and a redefinition of the critical zones where water resource conservation intersects with land-use decisions. For example, headwater areas of surface rivers are traditionally prioritized for water conservation. As existing literature suggests that out-of-basin land-use changes can influence river water within a basin^8,18,38^, a more integrative approach incorporating aerial rivers would identify the atmospheric basin of surface river headwaters as a strategic target for management^8,25^. Our results on critical upwind basins explicitly indicate where exactly the conservation efforts should focus. Regarding their connection to surface rivers, managing aerial rivers is essential for integrated water resources management.

**Fig. 6:**
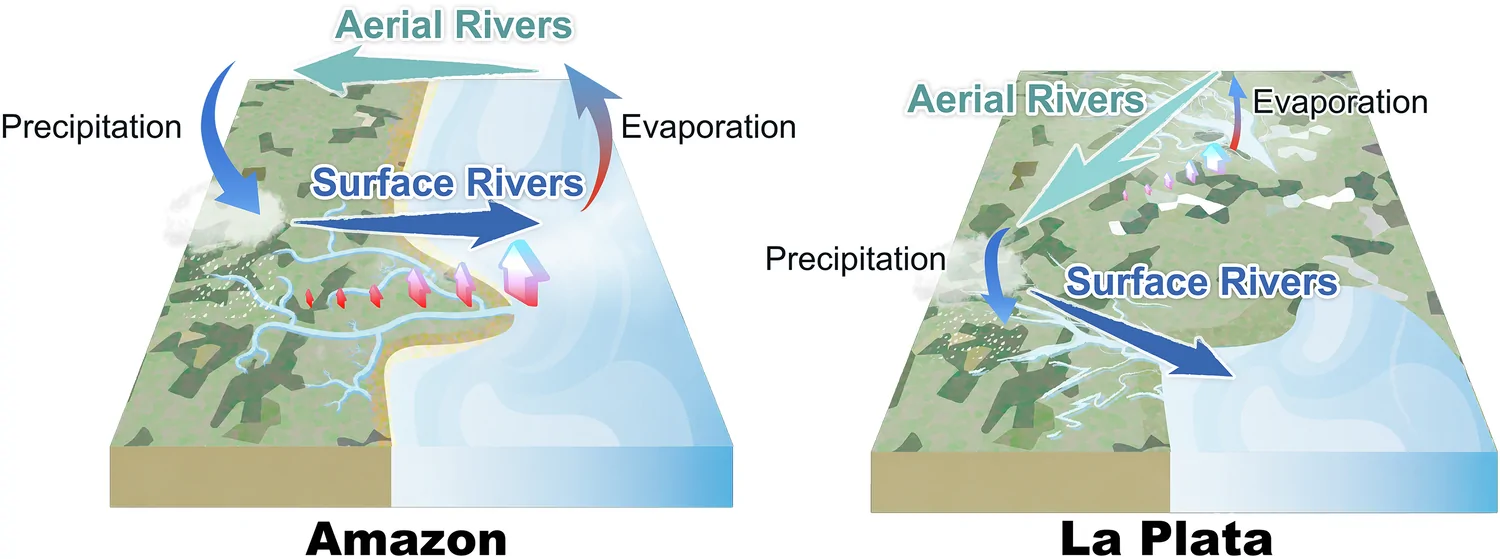
Hydrological integrity within basins: the connection between aerial rivers and surface rivers in the Amazon River Basin and La Plata River Basin. The spatial relationship between aerial and surface basins plays a crucial role in shaping the region’s hydrological integrity. In the Amazon Basin, the inverse flow directions of aerial rivers and surface rivers create a mutually reinforcing system that sustains moisture transport. However, this is not the case in the La Plata Basin, where the atmospheric basin supplying moisture to surface rivers lies outside the surface basin itself, limiting its ability to enhance inner-basin circulation.

Building on the connection between aerial rivers and surface rivers, we discuss two basin examples to complement the paradigm of hydrological integrity^39,40^ within the basin. This is exemplified by the major cases of the Amazon Basin and the La Plata Basin^41^. The downstream areas of the Amazon Basin function as upwind sources for upstream regions, supplying moisture through aerial rivers, with aerial and surface rivers together enhancing the long-term continental hydrological cycle (see Fig. 6). On the other hand, the La Plata River Basin presents a different case. While its headwater areas are located in a G4 region, offering potential for aerial river management to sustain water resources, its critical upwind areas lie outside the basin itself. As a result, the connection between aerial rivers and surface rivers does not enhance inner-basin hydrological circulation for the La Plata Basin^28,41^. In this case, the spatial displacement between aerial and surface basins can weaken hydrological connectivity and integrity.

In either case, the fluvial position under the aerial river regime can undermine power relations defined by the surface river regime previously. Upstream actors often gain leverage through dams or diversions that control flow timing and water allocation, leaving downstream users dependent on managed releases. Likewise, upwind regions could influence the moisture delivered by the aerial rivers to downwind regions. In the Amazon, the downstream region lies upwind of aerial rivers, a configuration that could shift existing power relations determined by surface rivers. However, this shift may, in some cases, create opportunities^24^ for cross-boundary collaboration, fostering a more integrated approach to managing water and moisture resources^16,30,42^. Beyond these two examples, the displacement between aerial river basins and surface river basins presents various combinations with different implications for hydrological integrity. This represents an interesting avenue for future research.

Our results suggest that the ecological tipping point^43,44^ varies across the continent due to differences in moisture drainage type. In terms of delivery intensity, a unit loss of moisture from a critical upwind basin is transmitted most efficiently to receivers in G1, followed by G2, G3, and finally G4. However, given their greater dependence on terrestrially sourced rainfall, G3 and G4 exhibit a greater proportional loss of moisture relative to their total rainfall for each unit area of deforestation within their critical upwind basins. Furthermore, although G3 and G4 are located downwind within the aerial river system, they also function as upstream regions of the surface Amazon River. From the perspective of the forest biotic pump^45^, deforestation in either downwind (surface-upstream) or upwind (surface-downstream) regions may weaken the hydrological cycle sustained by the coupled aerial and surface river systems in Amazonia (Fig. 6). This implication, however, must be considered alongside other key drivers of Amazonian tipping points, including soil properties such as fertility, texture, and water-holding capacity, as well as land-use practices and the integrity of the existing forest canopy^46,47^. These biophysical and ecological factors significantly mediate the forest’s response to external pressures such as deforestation and climate change, shaping the thresholds at which irreversible shifts may occur^48,49^.

In summary, we introduced a mathematical method to pinpoint critical upwind basins by identifying turning points on moisture drainage curves—thresholds beyond which upwind moisture contribution intensity sharply declines. By analyzing moisture drainage curves across South America, we identified four distinct drainage types, each characterized by a specific range of turning point thresholds. This demonstrates that the widely used one-size-fits-all thresholds are not universally applicable, underscoring the need for region-specific criteria.

The moisture drainage types we found here are geographic divisions shaped by long-term planetary atmospheric moisture transport, which is relevant for regional water resources. They correspond to fluvial positions within the continental aerial river system: headwater, drainage, outfall, and plain. While aerial rivers share certain characteristics with surface rivers, such as drainage systems and variations in speed, they also exhibit unique features; for instance, they carry the least moisture load when reaching the most downwind regions. It is clear that additional characteristics of aerial rivers and their connections with broader ecological systems remain to be explored. Furthermore, the features of aerial rivers may vary across different geographic regions. Nevertheless, the moisture drainage curve provides a practical approach for generating region-specific insights, supporting both the management of aerial rivers and further scientific investigation within the study area and beyond.

## Methods

### Study design

The investigation was carried out as follows. First, we employed a moisture tracking algorithm driven by observation-based climatic data to trace long-term moisture flows for describing the aerial rivers. Using the tracking outputs, we then derived moisture drainage curves and analyzed their evolution across the South American continent. Next, we identified distinct moisture drainage types and examined the features of turning points. Finally, we examined the characteristics of aerial rivers in each type and discussed the implications. Following, we outline the data and tools used to obtain moisture drainage curves and conduct the analysis. We also present the classification methods and explain how we calculated the variables used to examine the characteristics of aerial rivers in each moisture drainage type.

### Deriving moisture drainage curves

Moisture drainage curves describe how the moisture received in a given region is attributed to its upwind source regions. These curves are derived from long-term moisture tracking results, which enable the description of aerial rivers. We utilized a moisture tracking algorithm that is based on the water balance principle, the WAM-2layers^50^, to describe moisture flows and aerial rivers. Specifically, we employed WAM-2layers in a posteriori manner. Driven by historical evapotranspiration, precipitation, wind, and humidity data, the algorithm facilitates tracing of moisture flows at an Eulerian specification of the field. We traced moisture flows over the South American continent (30°W–85.5°W, 15°N–49.5°S) for the long-term period of 2006–2015. To ensure consistency, we used datasets with spatial coverage over the continent and coherent temporal coverage from 2006 to 2015 as input data for moisture tracking. We used the Global Precipitation Measurement (GPM) Integrated Multi-satellitE Retrievals for GPM (IMERG) Final Run product^51^ as precipitation input, while evaporation input was obtained from the Global Land Evaporation Amsterdam Model (GLEAM) v3.6a^52^. Wind and humidity data were derived from the ERA-Interim product^53^. All input data were adjusted to the operational resolution of WAM-2layers, with a temporal step of 3 h and a spatial resolution of 1.5°. The WAM-2layers algorithm provides a good representation of moisture flows on a continental scale^28,50^, which meets the objectives of this study.

Using the outputs of WAM-2layers, we calculated the average annual mean values to identify the preferential pathways of moisture flows over the long term. This approach allows us to capture the recurrent paths of moisture transport, i.e., aerial rivers. We then used the tracing results to identify the smallest upwind areas contributing to the percentages of continental-sourced rainfall received by any grid cell. Next, from the results for all percentages, we derived the relationship between the size of the upwind basin and its contribution to the continental-sourced rainfall for that grid cell. We extended the analysis to obtain moisture drainage curves across the South American continent (see Supplementary Fig. S2 for a map of curves). The data curves were transformed to natural logarithms and subsequently interpolated into 1000 evenly spaced intervals. The interpolated values were then converted back to their original scale using the exponential function; the rationale and sensitivity of this procedure are detailed in Supplementary D. To identify turning points, we first computed the local curve angle as the arctangent of the slope at each point. We then derived the rate of change of this angle and detected peaks in the derivative, which correspond to sharp shifts in curve orientation (see Fig. 3b). This indicates that the tangent angle rises markedly after the turning point. Peaks were selected using a data-driven prominence level (0.05), fulfilling the following criteria: (1) the number of peaks across all groups remains consistent within ±1% of this prominence, and (2) the first and second peaks on each curve are separated by at least 10% of the aggregated contribution. In this context, a peak is identified when its amplitude exceeds the surrounding baseline by at least 0.05. The conditions for detecting peaks corresponding to turning points were defined as follows:

*θ(x)* denote the arctangent of the slope for drainage curve, let

1 $$\theta ^{\prime} (x)=\frac{d\theta }{{dx}}$$

be its first derivative, a point *x*_*i*_ is identified as a local peak of the angle derivative if

2 $$\theta ^{\prime} \left({x}_{i-1}\right) < \theta ^{\prime} \left({x}_{i}\right) > \theta ^{\prime} \left({x}_{i+1}\right),$$

and the prominence condition is satisfied:

3 $$\theta ^{\prime} \left({x}_{i}\right)-\max \{\theta ^{\prime} \left({x}_{L}\right),\theta ^{\prime} \left({x}_{R}\right)\}\ge 0.05$$

where *x*_*L*_ and *x*_*R*_ are the nearest left and right positions such that $\theta ^{\prime} \left(x\right)\le \theta ^{\prime} \left({x}_{i}\right)$.

### Classification of curves

We measured the Calinski-Harabasz Score^54^ to determine the optimal number of clusters that best differentiate the moisture drainage curves. The Calinski-Harabasz score (also known as the Variance Ratio Criterion or the Calinski-Harabasz index) measures the ratio of between-cluster dispersion to within-cluster dispersion. Higher values indicate more distinct clusters. As shown in Supplementary Fig. S6, the highest Calinski-Harabasz score was observed at four clusters, suggesting that this configuration achieves the best separation among the clusters tested.

We applied the k-means method to partition moisture drainage curves into four clusters, minimizing variance within each cluster. This method groups curves by shape, treating them as vectors based on key features like slope and peaks. The algorithm starts with four centroids, assigns each curve to the nearest centroid, and updates cluster centers iteratively. Moisture curves are first converted into feature vectors by sampling points along each curve (e.g., 100 points form a 100-dimensional vector). The k-means algorithm then operates in this high-dimensional space, assigning curves to clusters based on Euclidean distance, which is computed by summing the squared differences between corresponding points. The centroids update iteratively, refining clusters until they stabilize. This approach ensures that curves with similar shapes are grouped together, minimizing variation within clusters. Via this approach, we obtained the four moisture drainage types, and we attributed each type to its respective location on the map (Fig. 2).

### Variables characterizing aerial rivers in statistical tests

To differentiate moisture drainage types, we derived key variables in Table 1 from long-term moisture tracking results to analyze aerial river characteristics. The average continental recycling ratio was obtained from the long-term (2006–2015) averaged WAM-2layers tracking results described earlier. For each grid cell, the model estimates the proportion of precipitation originating from continental evapotranspiration relative to that sourced from the ocean. The continental recycling ratio for the grid cell is then defined as the fraction of its total precipitation that comes from continental evapotranspiration. To estimate the average moisture traveling distance and time for aerial rivers to reach each moisture drainage type, we developed a simple point tracking algorithm based on the Lagrangian specification of motion. This tracking utilized the long-term averaged (2006–2015) ERA-Interim climatic data as input, ensuring consistency with the WAM-2layers tracking. The algorithm simulates the movement of moisture particles using the 843 hPa wind speed data from ERA-Interim as the velocity field for aerial rivers. We applied a backtracking approach by initializing particles at the grid cells of each moisture drainage type and tracing their paths backward to the ocean under a reversed velocity field. Each particle’s position was updated at 3-h intervals by solving the Lagrangian equations of motion using time-stepping algorithms. This method allowed us to record the moisture trajectory lengths and the residence times that emerge from the transport between ocean entry points and target grids. The water content variable was calculated by summing the moisture load along the pathway from the ocean to the target grid cells of each moisture drainage type, and is expressed as normalized annual precipitation depth in millimeters (mm) (see Supplementary E for mathematical formulation). Finally, delivery intensity was calculated as the group-average value of the total water content reaching each grid cell divided by the corresponding travel distance.

### Limitation

To analyze aerial rivers, we utilized moisture flow representations derived from numerical algorithms, acknowledging the inherent data and process uncertainties. While GPM and GLEAM datasets are generally considered robust^52,55^, they have certain limitations. The GLEAM product relies on remotely sensed soil moisture and vegetation indices, which introduce uncertainties—particularly in the densely vegetated Amazon, where satellite detection of soil moisture is challenging. Additionally, GLEAM tends to overestimate evapotranspiration in mountainous regions^52^. Conversely, GPM data is known to underestimate precipitation in mountainous areas^56^. Due to these input data limitations, the actual moisture drainage curves for G3 regions—many of which are mountainous—may more closely resemble those of G4 than currently represented by the available data. Since we employed an a posteriori approach and analyzed moisture flows on a long-term basis, our study implicitly accounts for the impacts of various processes that influence moisture transport, such as convection^57^, large-scale land-use change^58,59^, and extreme events, including droughts^59,60^, floods^61,62^, and fires^63^. However, the partition of each event and its influence on the moisture drainage is beyond the scope of this research.

The representation of climatological aerial rivers may be limited by the temporal coverage of our dataset. Over the 10-year study period, interannual variability in the continental recycling ratio is minor, and ENSO-related fluctuations remain modest (see Supplementary F). Nevertheless, the study period predominantly coincides with the cold phase of the Pacific Decadal Oscillation (PDO), interrupted by a brief warm phase in 2014-2015 that overlapped with an El Niño event and a modest (~2%) decline in the continental recycling ratio. Whether this decline reflects PDO modulation requires further investigation as additional decades of data become available. Concurrently, the Atlantic Multidecadal Oscillation (AMO) persisted in a positive phase, which typically suppresses moisture transport from the tropical Atlantic, enhances rainfall concentration over central-eastern Brazil, and alters precipitation patterns in southeastern South America^64^. Consequently, the aerial river influence identified in this study may be less pronounced than during a negative AMO phase, when moisture delivery to the SALLJ exit region (G4) tends to be enhanced^65,66^.

We conducted analyses of sub-period label stability, together with longer-period sensitivity tests, to evaluate the temporal robustness of the clustering results (see Supplementary G). First, we examined sub-period lengths of 1, 3, and 5 years. A 3-year window reduces much of the interannual variability, while a 5-year window already captures the principal patterns in a clear and concentrated manner, with entropy values indicating high temporal stability. For the longer-period analysis, the 10-year clustering patterns presented in the manuscript are in strong agreement with those derived from the 18-year GLEAM–GPM (2001–2018), the longest continuous gap-free period available for this input data pair. The difference in grouping labels is about 5% across the continent. To assess sensitivity to longer-term climate variability, we also examined the differences between 10-year (2006–2015) and 30-year (1990–2019) periods employing ERA5 reanalysis data; drainage type assignments differed for only approximately 5% of grid cells (see Supplementary Fig. S12). These findings suggest that aerial river drainage patterns are dynamic, yet retain a broadly stable large-scale structure across decades. However, differences in clustering between the ERA5- and GLEAM–GPM-based results exceed those observed across different temporal windows within the same dataset, indicating that input-data uncertainty contributes more to result variability than temporal-window length. Although this uncertainty should decline as large-scale observational datasets improve, the turning-point methodology remains robust in identifying critical upwind regions and drainage types across datasets. ERA5 was used primarily for temporal sensitivity testing, whereas GLEAM–GPM was retained for the main analysis because of its more observation-based character (see Supplementary H).

Regarding modeling uncertainty, Zemp et al. [ref. ^28^; see their Table 2, p. 13344] demonstrated that estimates of the Amazon Basin regional recycling ratio from WAM-2layers are consistent, falling within one standard deviation of values obtained using atmospheric general circulation models and other online tracking methods. To assess the robustness of our clustering results, we applied a bootstrap resampling approach, which repeatedly resamples the data with replacement and recalculates the clustering for each iteration. The results indicate high stability: 83% of the grid cells had a majority probability of 1, meaning the dominant class appeared in all 1000 runs. Detailed bootstrap results are provided in Supplementary I, which also discusses clustering results for solutions with more clusters that mainly reflect subdivisions of existing patterns rather than new ones. We focused on the geographical patterns of moisture drainage from aerial rivers across the South American continent. For local applications at scales smaller than the municipality level, a downscaled analysis would be necessary. Nevertheless, our continental-scale analysis demonstrates that moisture drainage patterns vary geographically, reflecting distinct regional characteristics. Given the diversity across continents and landscapes, additional moisture drainage types likely exist beyond those identified in this study. This highlights the need for future research to extend the exploration of aerial rivers to other regions. Importantly, the research framework developed here provides a robust foundation for investigating these dynamics across diverse geographies.

## Supplementary information


Supplementary Information
Transparent Peer Review file


## Data Availability

The raw data supporting the findings of this study are publicly available and were downloaded from the official websites of Integrated Multi-satellitE Retrievals for GPM (IMERG; https://gpm.nasa.gov/data/imerg) and the Global Land Evaporation Amsterdam Model (GLEAM; https://www.gleam.eu/). All data required to evaluate the conclusions of this study are present in the paper or the Supplementary Materials.

## Code availability

The WAM-2layers model code is available at https://github.com/WAM2layers/WAM2layers. The simple point tracking algorithm used to characterize aerial rivers, based on a Lagrangian specification of motion, is available on Zenodo https://doi.org/10.5281/zenodo.17547644.
