# Supplementary material for: Hydrological regimes and drainage systems of aerial rivers across South America
Source: Nat Commun. 2026 Aug 3;17:9341. doi: 10.1038/s41467-026-76303-y (PMC13529790; doi:10.1038/s41467-026-76303-y)
Supplement: Supplementary file 1 [file 41467_2026_76303_MOESM1_ESM.pdf]

Supplementary

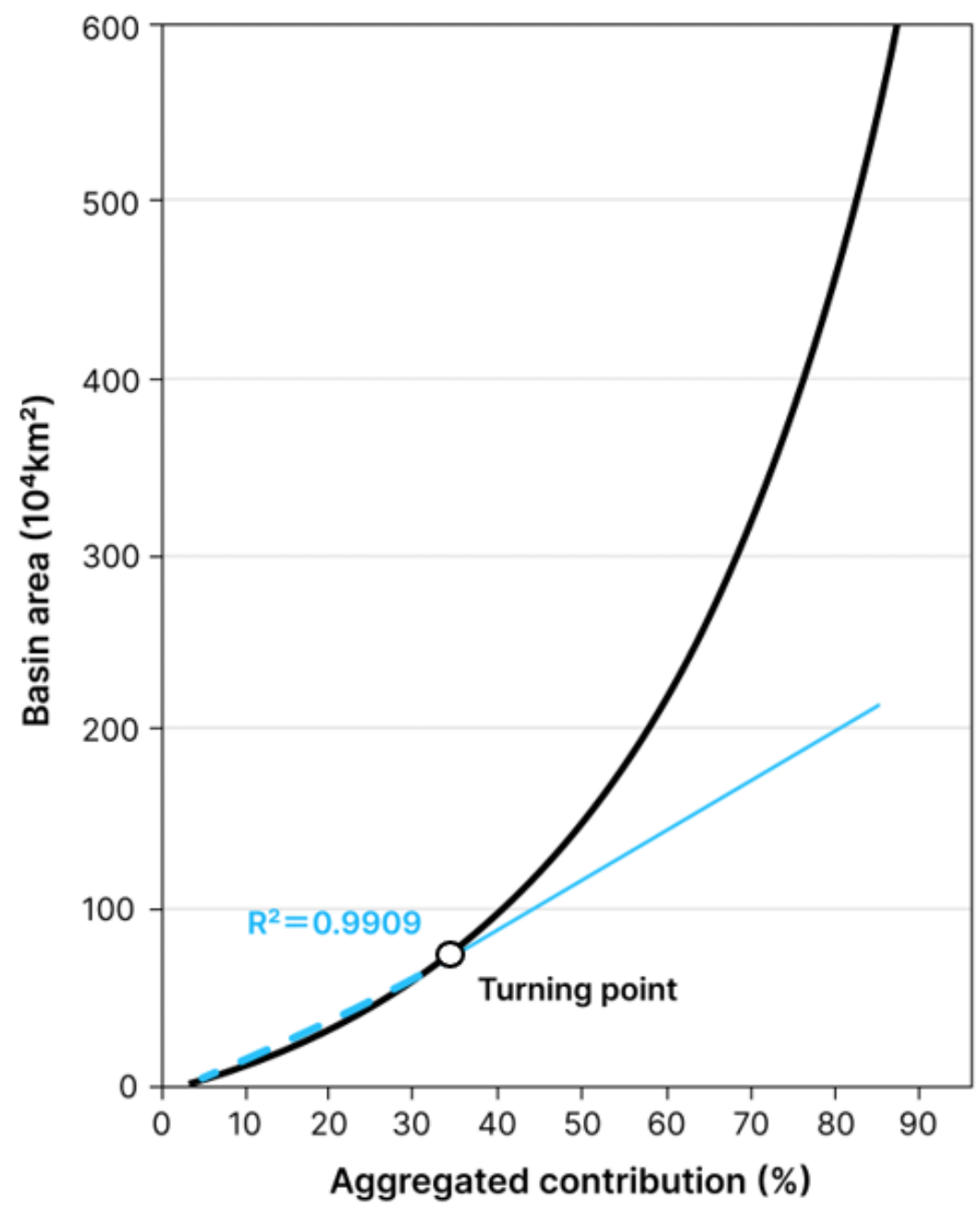

Fig. S1. Moisture drainage curve example: Santa Cruz de la Sierra (Bolivia). Adapted from Figure A2 in *Aerial river management by smart cross-border reforestation* by Weng et al., Land Use Policy, 84 (2019) [1], under the CC BY 4.0 license. The curve represents the minimum land area required to supply a given percentage of terrestrial-sourced rainfall received by Santa Cruz de la Sierra (Bolivia). Beyond the turning point, the land area needed per unit of moisture contribution increases sharply. This turning point thus serves as a scientific threshold, delineating the most influential upwind regions for more effective water management.

A. Map of moisture drainage curves

Fig. S2. presents the results of moisture drainage curves across the South American continent, arranged by their geographic location. It allows for the observation of how the curves evolve across the continent.

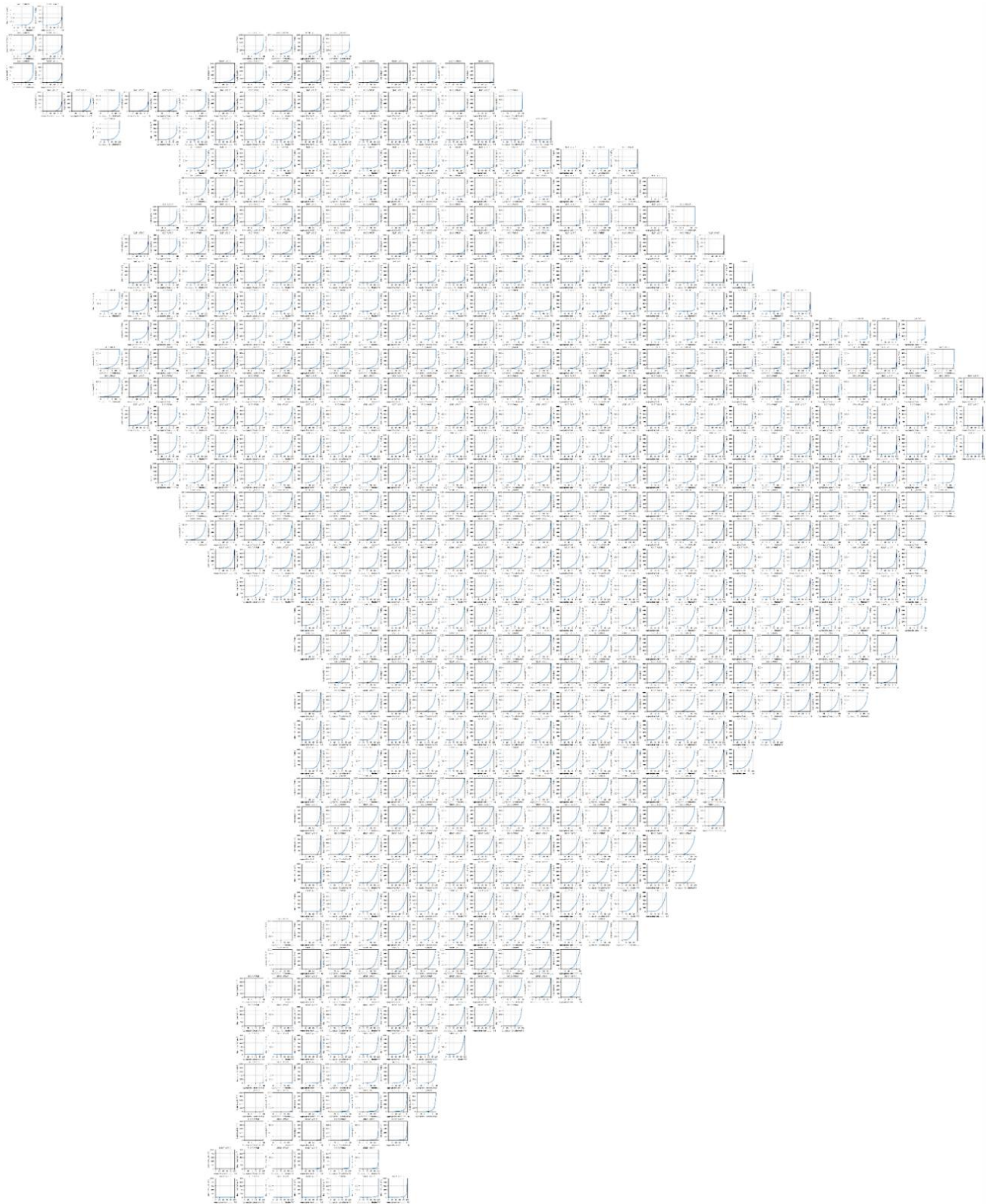

Fig. S2. Map of moisture drainage curves.

Distribution of moisture drainage curves across the South American continent (original size figure link on Zenodo: https://doi.org/10.5281/zenodo.17547634 [2]).

B. Fixed thresholds, especially larger ones, greatly affect upwind region delineation

We set a specific percentage of continental-sourced moisture as a threshold and calculated the corresponding upwind basin size contributing to that threshold for each data point. Figure. S3a. illustrates the results across the range of 0-100% of continental-sourced moisture. This analysis highlights how fixed threshold selections in the literature can lead to vastly different conclusions (see Fig. S3). We performed frequency counts for each moisture drainage type and present the profiles for 40% and 70% thresholds as examples in Fig. S3b and S3c.

For example, at the 40% threshold (see Fig. S3b), we estimated the upwind basin size required to contribute 40% of the terrestrial-sourced rainfall for each data point. We then counted the frequency and used colors to label the composition of moisture drainage types. As shown in Fig. S3b, G1 reaches the 40% threshold quickly with a small upwind basin size, while G2, G3, and G4 require larger upwind basins to achieve the same share. In other words, the upwind basin contribution per unit area is highest in G1, lower in G2 and G3, and lowest in G4. This difference becomes more pronounced at higher thresholds. As seen in Fig. S3c, at the 70% threshold, the upwind basin size for G4 is generally double that of G3 and triple that of G2.

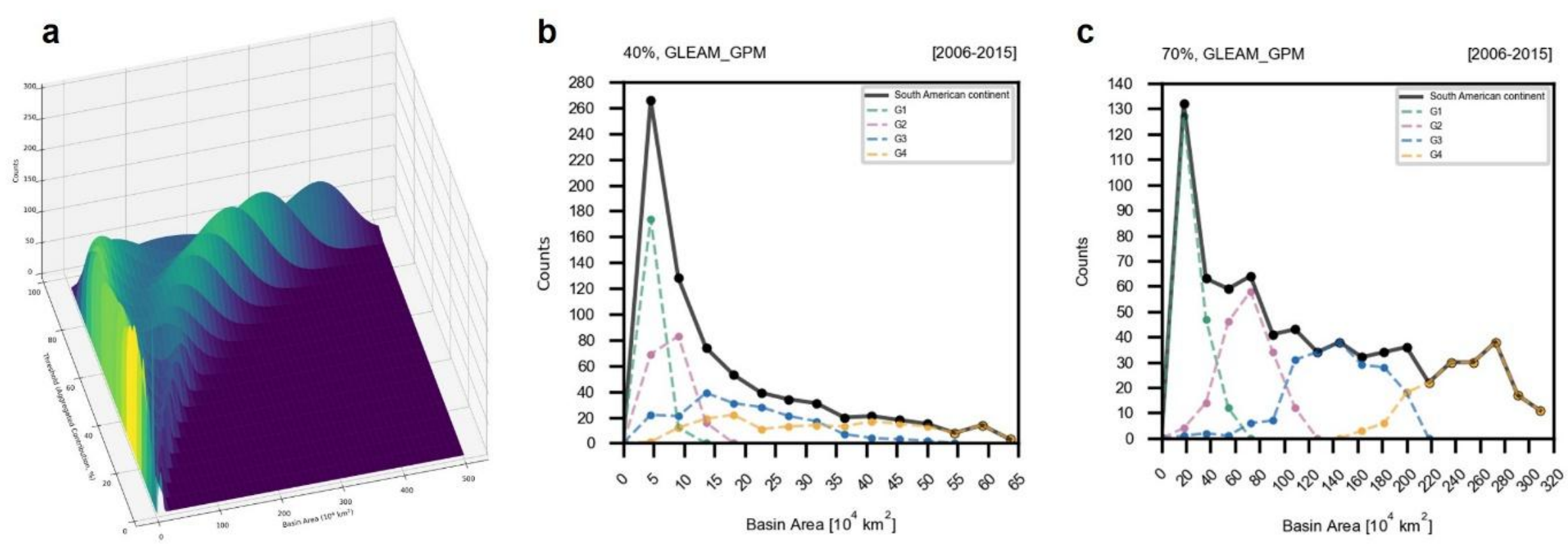

Fig. S3. (a) Relationship between upwind basin size, aggregated contribution (threshold), and counts. (b) 40 % threshold profile. Size of the upwind basin required to contribute 40% of continental-sourced rainfall at each data point, along with the frequency counts for each moisture drainage type. **(c) 70% threshold profile.** Size of the upwind basin required to contribute 70% of continental-sourced rainfall at each data point, along with the frequency counts for each moisture drainage type.

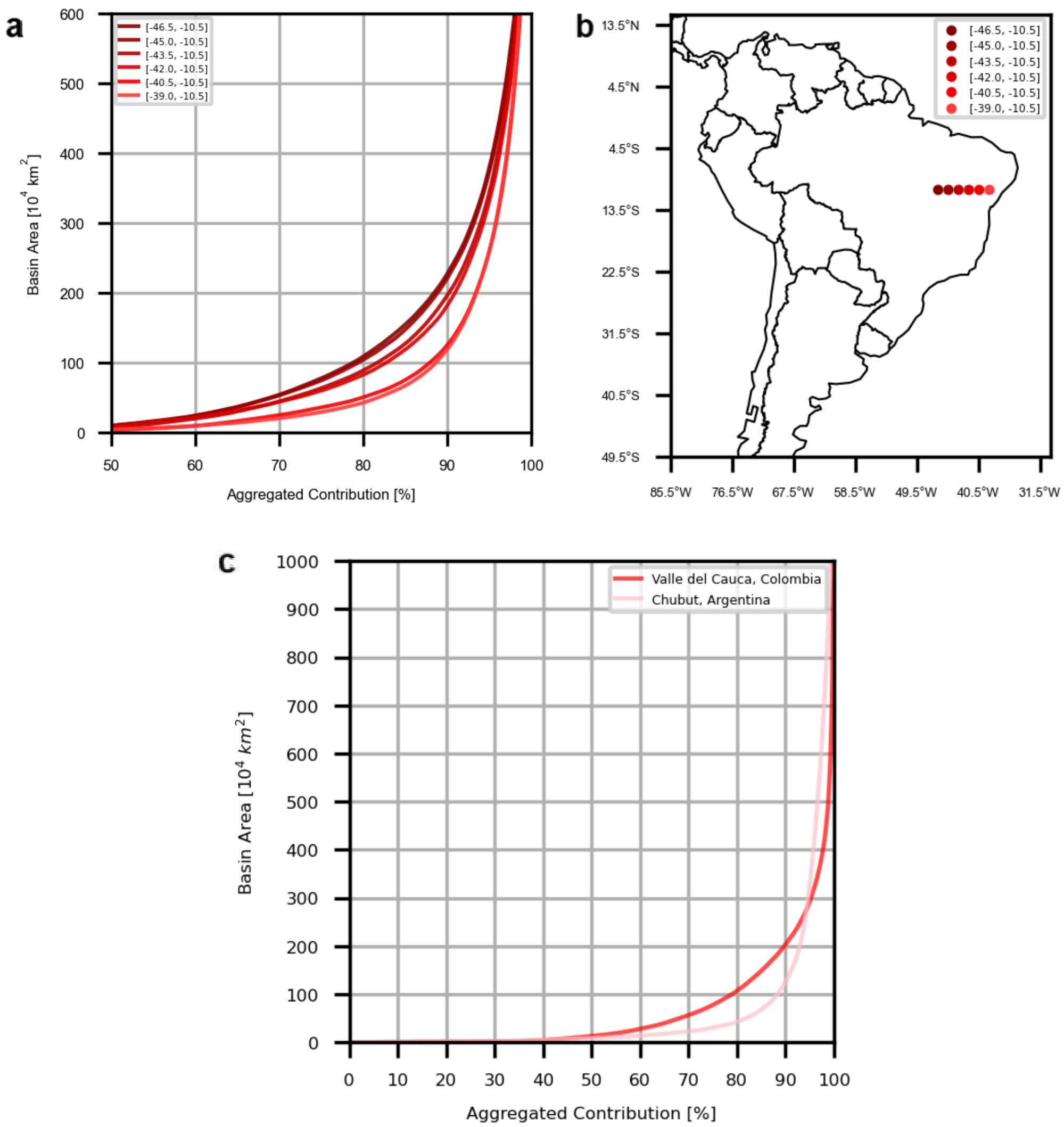

Fig. S4. (a) Evolution of G2 drainage curves from the G1 transition toward inland areas (following 10.5°S and prominent wind direction). (b) The respective locations of the curves. The G2 drainage curves initially shift toward lower x-values, indicating a reduced contribution from G2's critical upwind basins relative to G1, while upwind moisture contribution intensity remains stable under expanding atmospheric drainage. As aerial rivers progress inland, the curves exhibit an upward shift toward higher y-values, reflecting diminished per-area upwind contributions and a decline in upwind moisture contribution intensity during continued drainage expansion. **(c) The example of the variations within G2**. The G2 curve in Chubut, Argentina shows where the turning point shifts left as atmospheric drainage expands while upwind moisture contribution intensity remains stable. In contrast, the G2 curve in Valle del Cauca, Colombia reflects a decline in upwind moisture contribution intensity occurring alongside the expansion of atmospheric drainage.

C. Cases for identifying critical upwind basins for aerial river management

In the main text, we use Sorriso Municipality (Fig. 4b)—the largest soybean-producing municipality in Brazil—as an example to demonstrate the aerial river management framework for sustaining regional crop systems. Located in South America, one can observe from Fig. 2a. that this region is classified as G3. For G3 regions, critical upwind basins account for 18-24% of the total rainfall. If this range aligns with the authority's needs and capacity, one can then follow the framework for aerial river management outlined in Fig. 4. For regions outside of South America, the procedure outlined in Fig. 4a should be followed directly.

First, we follow the procedure in Fig. 4a and use the algorithm to obtain the moisture drainage curve for Sorriso's grid cell (Fig. 4b). Next, we analyze local angle changes to identify the turning point, which occurs at 47%. This allows the authority to assess whether the size of critical upwind basin (337,500km^2) exceeds its management capacity. Once a manageable basin size is confirmed, we can map it out (as demonstrated in Fig. 4b and Fig. 5, which shows the entire critical upwind basin) to delineate the regions for targeted management. From there, the authority can determine management instruments, such as conservation or strategic reforestation, and estimate the moisture content to be regulated along the aerial river.

For G1 (Fig. 5a, 5b), G2 (Fig. 5c, 5d), and G4 (Fig. 5g, 5h) types, we provide an illustrative region for each case—Georgetown for G1, Puerto Nariño for G2, and Asunción for G4—to demonstrate how their critical upwind regions can be identified (Fig. 5 in the main text).

Assessing downstream runoff impacts from strategic reforestation in critical upwind basin

Using reforestation as an example of targeted management, reforestation potential within Sorriso's critical upwind basin can first be identified using forest restoration potential datasets, such as Bastin et al. (2019) [3]. Potential environmental impacts of such interventions must then be evaluated. One critical concern is that reforestation can alter regional water balances and may reduce downstream water availability, particularly in areas already subject to water stress. Accordingly, downstream water-risk conditions should be assessed using relevant datasets and methodologies [4], such as Aqueduct Water Risk Atlas [5] and WaterGAP [6]. Critical upwind basins whose downstream catchments have high overall water risk should be excluded from reforestation planning. For Sorriso's critical upwind basin, the downstream catchment is classified as low-medium risk in the Aqueduct Water Risk Atlas [5], suggesting minor existing water stress. Where downstream regions are not already under severe water stress, managers should assess the potential trade-offs affecting downstream water availability [4]. Downstream catchments may experience both evapotranspiration losses associated with expanded forest cover and precipitation gains through aerial river within the catchment [7]. Managers may consult published frameworks, such as the methodology of Weng et al. (2019) [1] or the dataset of Hoek van Dijke et al. (2022) [8], to evaluate these interacting effects. Critical upwind basins in which reforestation is projected to significantly reduce downstream water availability should be excluded from intervention planning. Using the dataset developed by Hoek van Dijke et al. (2022) [8], the projected reduction in annual runoff reaching Santarém resulting from reforestation in Sorriso's critical upwind basin is approximately 1.2%, indicating a rather limited hydrological impact. In this context, a downstream runoff change of this magnitude should be incorporated into a broader impact assessment to guide management decisions for Sorriso's critical upwind basin. Such an assessment should involve stakeholders across affected regions to evaluate potential trade-offs. Management plans should also include mitigation measures and adaptive management frameworks to monitor hydrological outcomes and address any adverse effects on downstream water resources [4].

Comparable assessment approaches should also be applied to environmental impacts beyond hydrology.

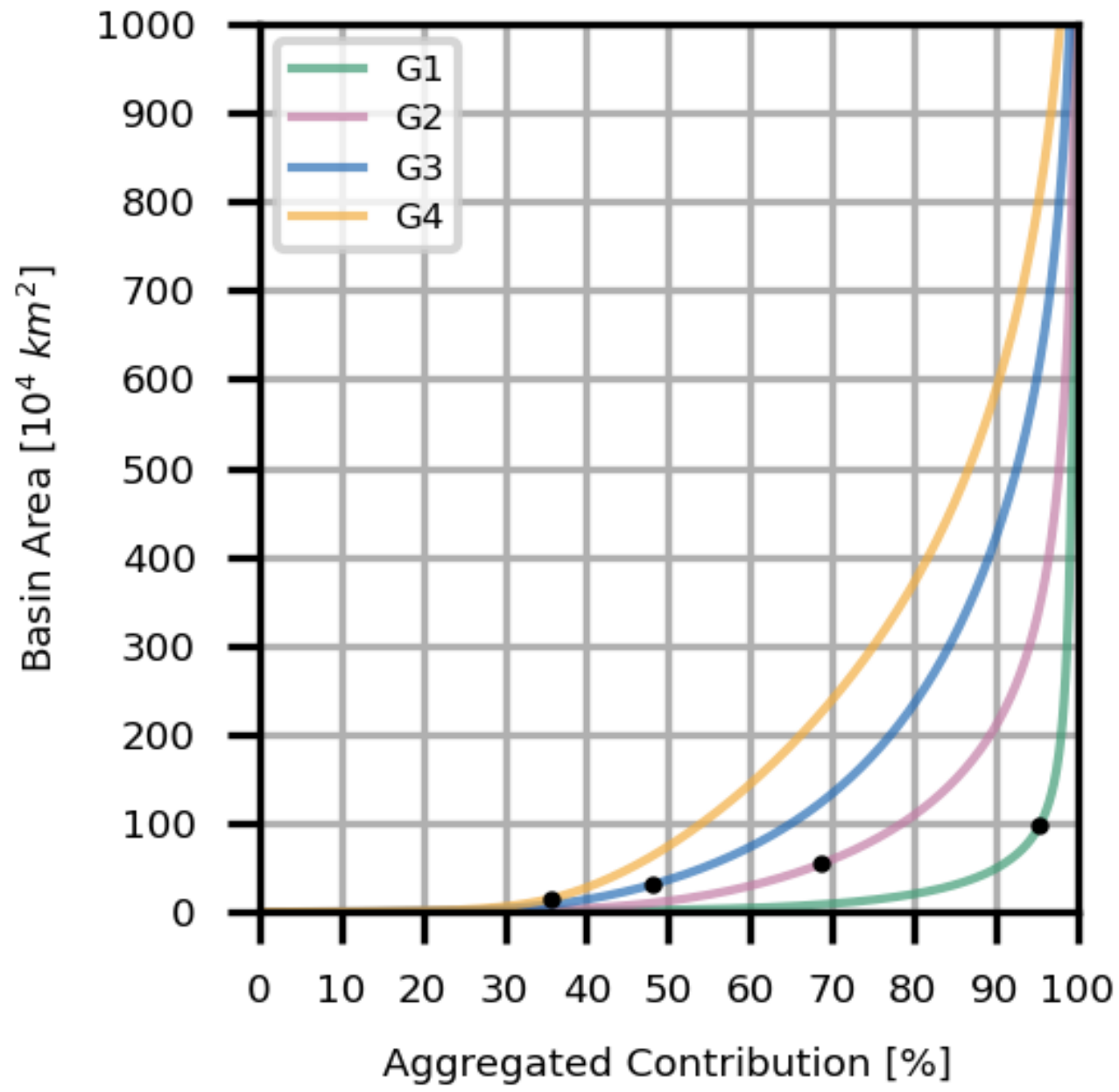

Fig. S5. The median curve of each moisture drainage type.

As aerial rivers travel further, the moisture drainage curves evolve, transitioning from G1 to G2, G3, and finally to G4. The turning points represent the median value of the tuning points for each type.

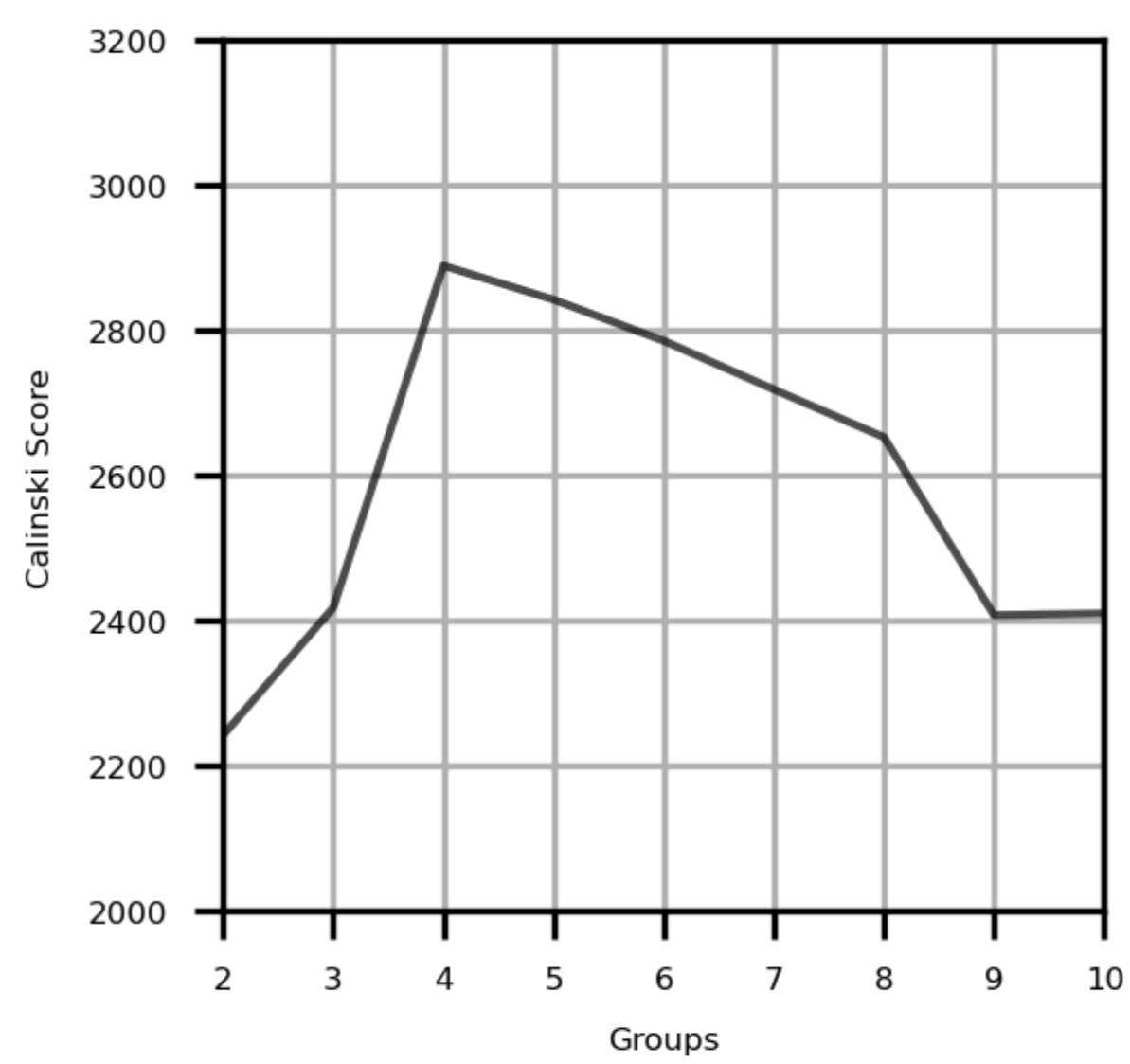

Fig. S6. Calinski-Harabasz score for moisture drainage curve clustering. The score indicates that the optimal number of clusters, yielding the highest score, is four. These four clusters best differentiate the moisture drainage curves.

D. Rationale and sensitivity assessment of the log-transformed data preprocessing

Here, we clarify the physical and mathematical rationale underlying the preprocessing procedure and assess its influence on turning point identification.

Mathematical rationale for log-transformed preprocessing

The preprocessing pipeline consists of three steps applied to the raw moisture drainage curves generated by the WAM-2layers model. First, each raw moisture drainage curve is converted into a cumulative distribution function (CDF), which is then transformed using the natural logarithm. Second, linear interpolation is applied to the log-transformed CDF. Third, the interpolated curve is transformed back to its original scale using the exponential function and differentiated to obtain the corresponding probability density function (PDF) and local curve angles.

Interpolation is performed in log-CDF space because moisture accumulation along an aerial river pathway is typically highly skewed, with rapid accumulation in dominant source regions followed by a long tail of weaker contributions. Under these conditions, interpolation on the original scale would disproportionately weight segments with large absolute values. By contrast, interpolation in log-CDF space better preserves the relative structure of both high- and low-contribution segments. Mathematically, an approximately linear relationship in log-CDF space implies an exponential- or Pareto-like scaling of cumulative moisture accumulation. The corresponding PDF, obtained by differentiation, exhibits a lower-order, smoothly varying structure consistent with the statistical-mechanical behavior of large-scale atmospheric diffusion and advection. This procedure suppresses localized, high-frequency numerical variability associated with the discrete model grid while preserving the macroscopic climatological characteristics of aerial river basins.

Comparison of turning point identification using log-transformed preprocessed and raw data approaches

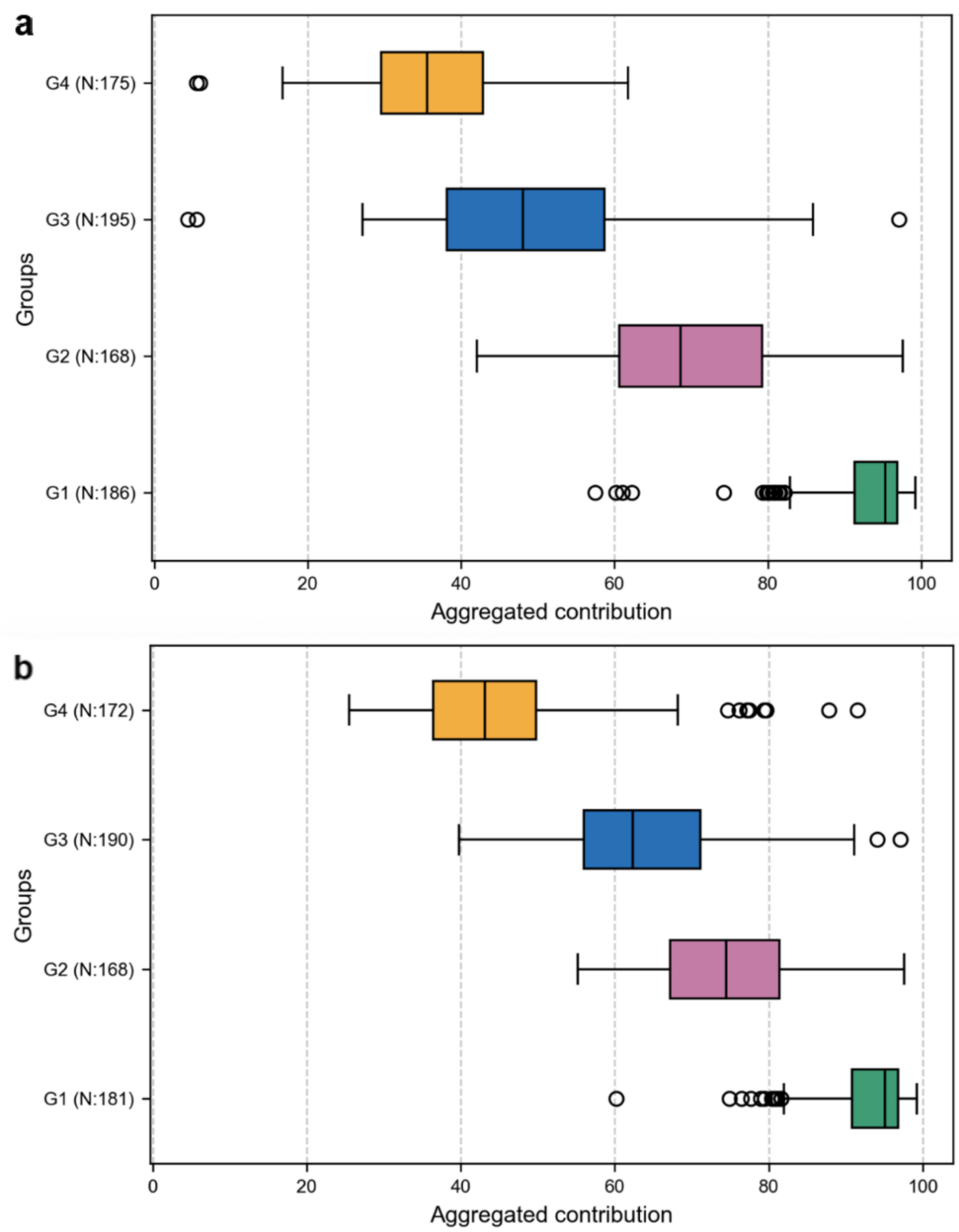

Fig. S7. (a) Boxplots of turning points derived from the log-transformed moisture drainage curves. (b) boxplots of turning points derived from the raw data.

The turning points derived from the preprocessed curves were compared with those obtained directly from the raw data (Fig. S7). Overall, the two approaches produce generally consistent conclusions, with minor differences in the distribution tails and outliers. The number of curves assigned to each drainage type differs by less than 3% between the two approaches, indicating that the classification is stable with respect to preprocessing. The preprocessed analysis produces lower median turning point values for G3 and G4 and show changes in some extreme values. Nevertheless, the interquartile ranges and relative separation among the groups remain broadly comparable. These results indicate that preprocessing primarily preserves the dominant group structure and relative contribution patterns.

The most pronounced difference occurs in G3, which is characterized by more complex terrain and transitional moisture delivery regimes. This suggests that G3 is more sensitive to local-scale variability in the raw tracking outputs. The complexity in moisture delivery behavior within G3 may provide an interesting avenue for future research.

Nevertheless, although calculating angles changes directly from the raw data is technically straightforward, we note that using raw, non-log-transformed curves for turning point detection introduces additional mathematical uncertainty. The raw tracking outputs contain high-frequency, small-scale spatial features and step-like discontinuities associated with the discrete model grid. Applying angle-change analysis to these raw fields amplifies such variability, producing multiple artificial local peaks that obscure the identification of the macroscopic turning point. This sensitivity is reflected in the missing value in the raw-data analysis (Fig. S7). To address this issue, we applied a physically informed smoothing procedure to the moisture drainage curve to suppress grid-scale artefacts while retaining the large-scale structure relevant for turning-point identification.

E. Mathematical formulation for calculating moisture content

The governing equation for the path-integrated water content of grid cell i, W_i, is defined as follows, with its physical upper bound given by Equation (S1):

$$W_i = \frac{\sum_{j \in V_i} m_{ji} \times 1000}{A_i} \leq Pr_i \quad \text{(S1)}$$

where:
W_i = water content through terrestrial source accumulated by grid cell i.
V_i = the set of unique grid cells along the backtracked pathway to target cell i by simple point-Lagrangian tracking algorithm.
$j \in V_i$ grid cell j belongs to V_i.
m_{ji} = moisture contribution from grid cell j to grid cell i, recorded by WAM-2layers output.
Pr_i = the total precipitation at the target cell i.
A_i = size of grid cell i (m^2)

Here, m_{ji} denotes the individual source-to-sink water-content contribution (m^3) from upwind grid cell j to target grid cell i, as obtained from the WAM-2layers tracking matrix. Because the algorithm aggregates these contributions across the set of unique grid cells, V, traversed by the backtracked trajectory, double-counting is avoided.
By expressing W_i as an equivalent annual precipitation depth (mm), obtained by dividing the integrated moisture volume by the area of the target grid cell i, the formulation becomes independent of grid-cell area. Its maximum possible value is physically constrained by the mean annual precipitation at the target grid cell, Pr_i.

F. Interannual variation of continental recycling ratio and ENSO influence (2006–2015)

According to NOAA Physical Sciences Laboratory, between 2006 and 2015, the region experienced roughly two cycles between El Niño and La Niña phases, with 2007 and 2010, and 2015 classified as El Niño years, and 2008 and 2011 as La Niña years. Figure S8 presents the ten-year anomalies of the annual mean continental recycling ratio, showing that interannual variation remains small. The ten-year average annual continental recycling ratio is 0.35, with a standard deviation of 0.012, yielding a coefficient of variation of approximately 3.5%. These anomalies show little correspondence with ENSO phases. However, the Pacific Decadal Oscillation (PDO) briefly shifted to a warm phase in 2014–2015, coinciding with an El Niño event and a modest (~2%) decline in the continental recycling ratio. Whether this decrease is attributable to the PDO remains uncertain and will require further investigation as longer-term datasets become available.

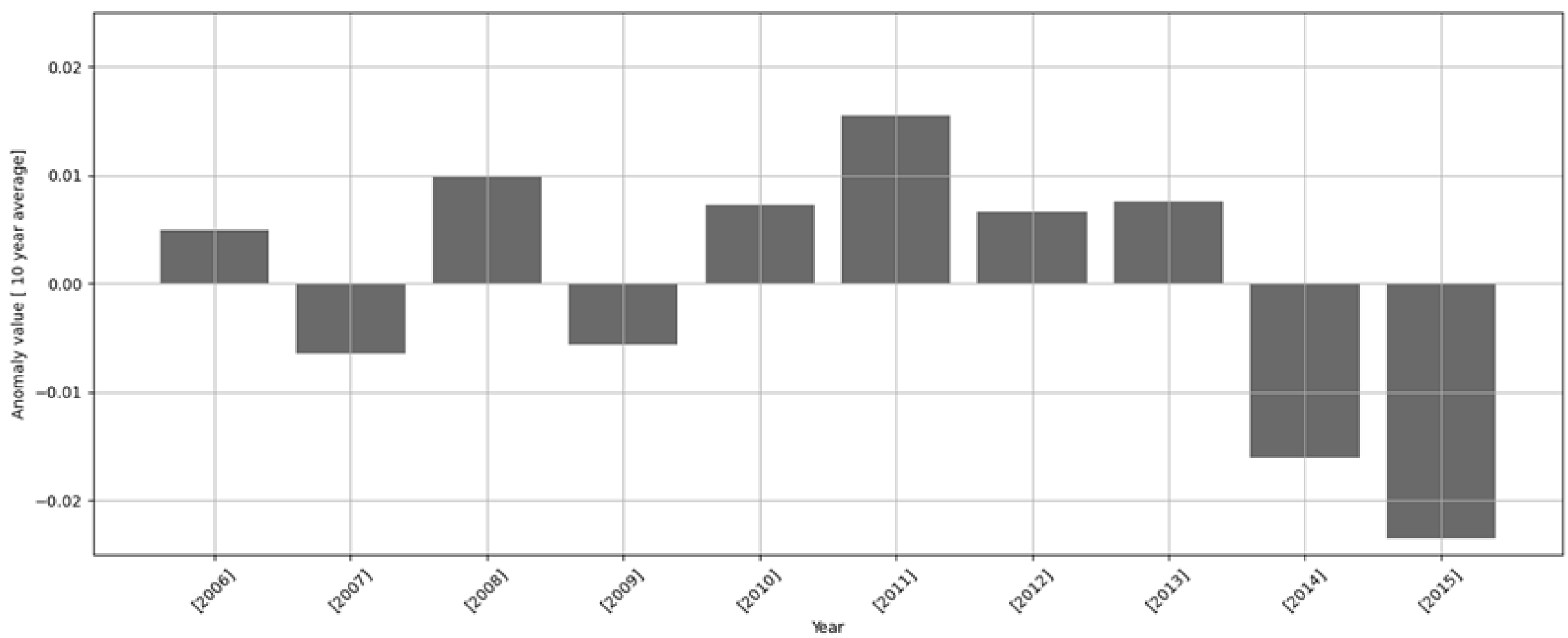

Fig. S8. Ten-year anomalies of the annual mean continental recycling ratio (2006–2015). Minor interannual variation is observed with limited correspondence to ENSO phases, though a modest (~2%) decrease occurs in 2014–2015, coinciding with a PDO warm phase and an El Niño event.

G. Evaluation of temporal stability

We conducted a sub-period label stability analysis to evaluate the temporal robustness of the clustering results. For each temporal window (1, 3, and 5 years), K-means clustering results were mapped onto the original four-group baseline derived from the 10-year mean (Fig. S9). In the 1-year case, the spatial pattern is more strongly affected by interannual variability and exhibits higher entropy. As the temporal window increases to 5 years, entropy decreases to 0 over most regions. The remaining zones of relatively high entropy (pink-purple areas in Fig. S9) correspond to transitional regions between drainage types. These should therefore be interpreted as real dynamic boundaries rather than as artifacts of the clustering algorithm.

The boxplot (Fig. S10) provides a statistical quantification of the stability pattern shown in Fig. S9. For the four-class case, normalized Shannon entropy values below 0.2 indicate high temporal

stability. Mean entropy across the study domain declines substantially and rapidly, from 0.42 in the 1-year window to 0.17 in the 5-year window, indicating strong convergence of the classification at the 5-year timescale. Since the 5-year window already captures the principal patterns in a clear and concentrated manner, the 10-year period used in our analysis can be considered statistically robust.

For longer-period tests, the 10-year clustering patterns used in this study are in strong agreement with those derived from the 18-year GLEAM–GPM record (2001–2018), the longest continuous period available without data gaps, with only about a 5% difference in grouping across the continent. We further compared the results derived from ERA5 reanalysis data for 10-year (2006–2015) and 30-year (1990–2019) periods, and found the clustering structure to remain similarly robust, again with only about a 5% difference. These results suggest that aerial river drainage patterns are dynamic, yet maintain an overall stable large-scale structure across decades.

It should be noted, however, that the clustering difference between the ERA5-based results and those based on the GLEAM–GPM pair used in the manuscript is larger than the difference associated with extending the temporal window from 10 to 30 years. This indicates that uncertainty in the input data contributes more to result variation than the choice of temporal window length. This source of uncertainty is expected to decrease as large-scale observational datasets continue to improve. Nevertheless, the turning-point methodology proves robust in identifying critical upwind regions and drainage types across different input datasets. In this context, the ERA5-based reanalysis experiment was included primarily to assess sensitivity to longer-term climate variability, whereas the GLEAM–GPM dataset was retained for the main analysis because of its more observation-based character.

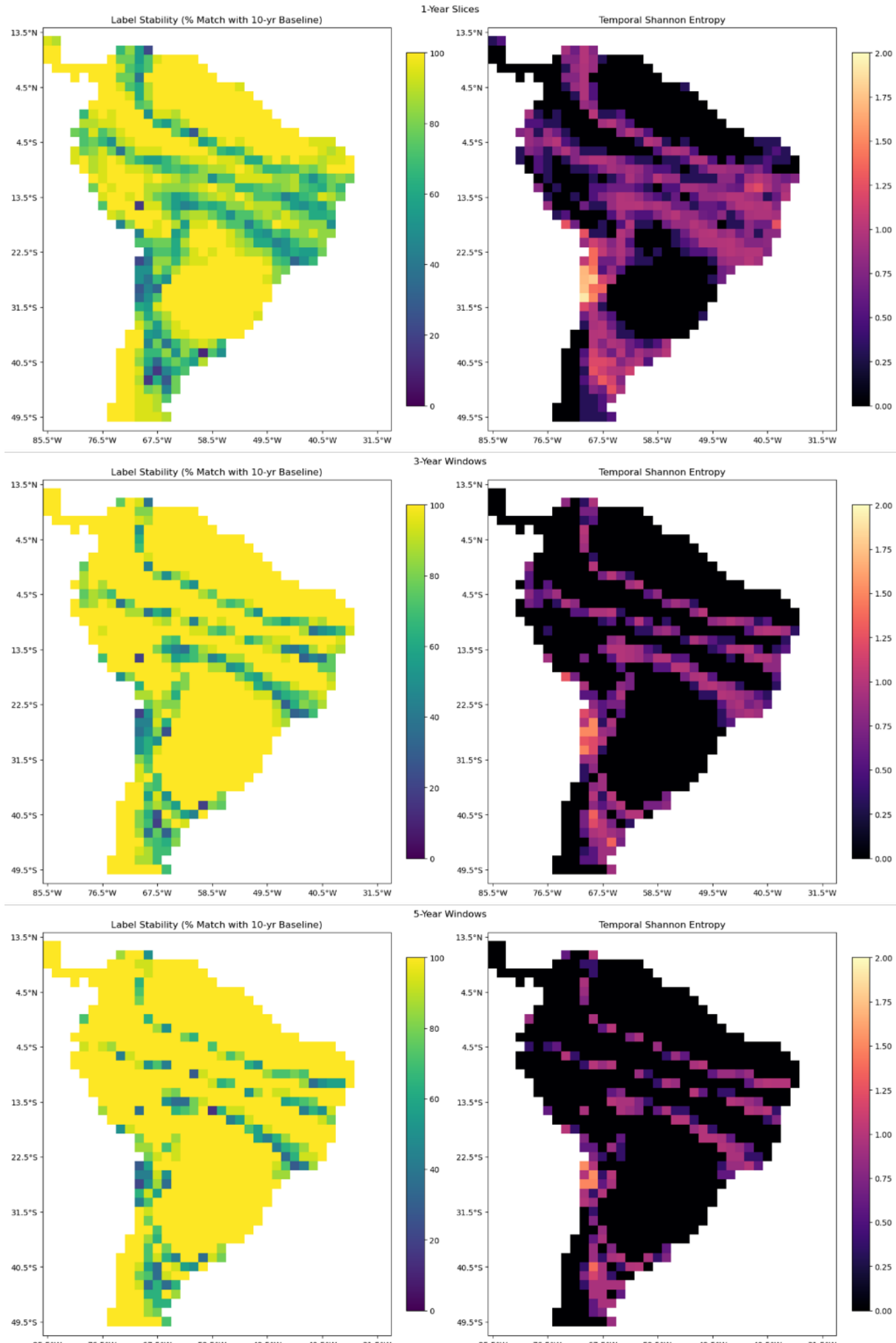

Fig. S9. Sub-period analysis of clustering stability and temporal classification entropy using 1-, 3-, and 5-year moving windows. Stability is defined as the proportion of temporal slices in which a grid cell is assigned to the same class as in the 10-year baseline. Temporal variability in class assignment is further quantified using Shannon entropy. An entropy value of 0 indicates complete temporal stability, with the grid cell remaining in the same class throughout all temporal slices, whereas higher entropy values indicate more frequent shifts among the four classes over time. The three panels show the smoothing effect associated with increasing temporal window length.

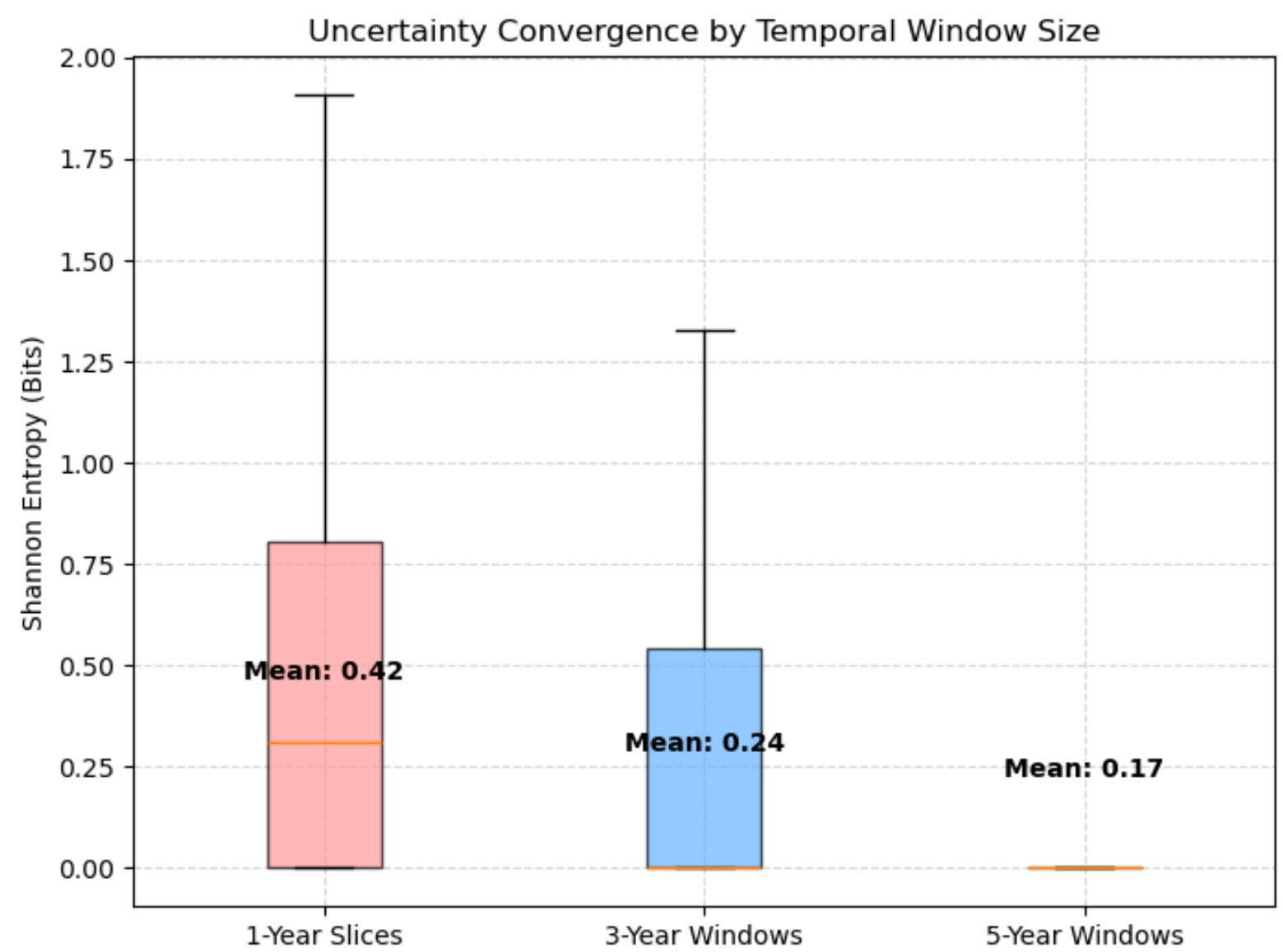

Fig. S10. Statistical quantification of clustering stability across 1-, 3-, and 5-year moving windows. Mean entropy across the study domain declines substantially and rapidly, from 0.42 in the 1-year window to 0.17 in the 5-year window. In the four-class case, normalized Shannon entropy values below 0.2 indicate high temporal stability, suggesting that the principal patterns are captured clearly and in a concentrated manner at the 5-year timescale.

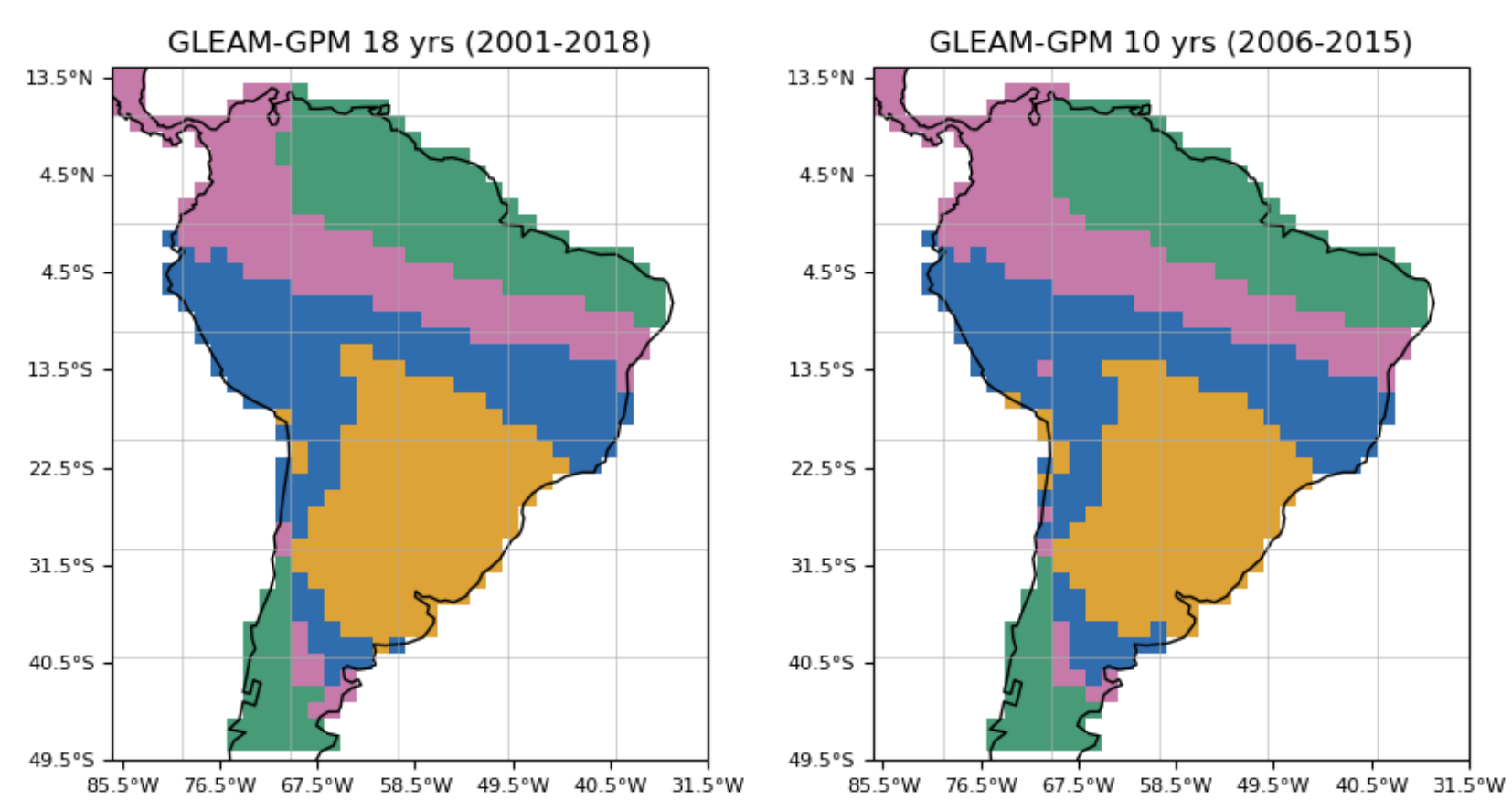

Fig. S11. Comparison of 10-year and 18-year clustering results based on the GLEAM–GPM experiments. The spatial patterns show strong agreement, with only about a 5% difference in labeling across the continent.

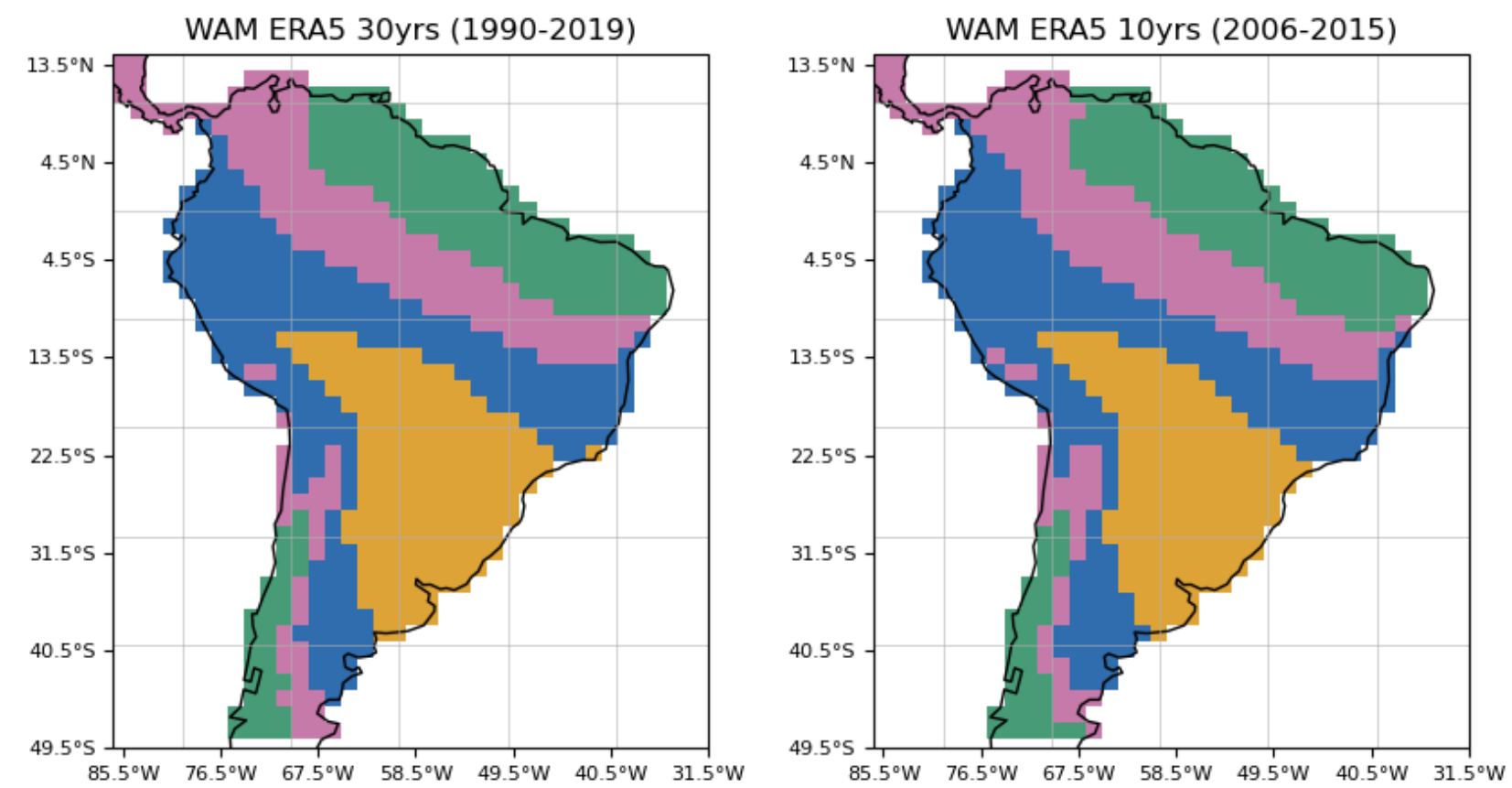

Fig. S12. Comparison between the 10-year and 30-year clustering results based on the ERA5 reanalysis experiments. The spatial patterns show strong agreement across decades, with only about a 5% difference in class labels across the continent.

H. ERA-5 30 years analysis of temporal uncertainty

To assess the sensitivity of the identified aerial river drainage structures to the length of the analysis period, we conducted an additional analysis using ERA5 over a continuous 30-year period (1990-2019). ERA5 was selected for this purpose because it provides a sufficiently long and uninterrupted record, allowing longer-term temporal variability to be examined. However, ERA5 is a model-based reanalysis product that combines numerical weather prediction with data assimilation and should therefore not be interpreted as observational evidence.

Accordingly, the ERA5 analysis is included solely as a supplementary assessment of temporal sensitivity and is not intended to replace the primary observation-based GPM–GLEAM analysis or to support a quantitative comparison between the two datasets. Differences in the precipitation and evaporation inputs derived from ERA5 and GPM–GLEAM have been documented in previous studies [9] [10], while regional uncertainties in ERA5 have been discussed by Baker et al. (2021) [11] and Lavers et al. (2022) [12].

Comparisons across different analysis periods within each dataset, namely the 10-year and 30-year ERA5 analyses, and the 10-year and 18-year GPM–GLEAM results, indicate that the principal aerial river drainage structures remain robust over longer temporal windows. The larger discrepancies arise from differences between datasets rather than from the length of the analysis period (Figs. S11 and S12)

The moisture tracking analysis was repeated using ERA5 reanalysis data [13] for two periods, 2006–2015 and 1990–2019, while retaining the same spatial domain and WAM-2layers tracking configuration [14]. ERA5 evaporation (E), total precipitation (P), and the wind and humidity fields used to drive the tracking were ingested at their native 0.25° spatial resolution. To ensure comparability with the original analysis, the tracked evaporation and tagged precipitation fields were aggregated from 0.25° to the 1.5° analysis grid (44 × 38 cells) using area-conserving 6 × 6 block averaging, which preserves the kg m^{-2} (≡ mm) flux magnitudes. The aggregated fields were then used to derive monthly and annual-mean climatologies expressed in (mm yr^{-1}). Backward trajectories were driven by the climatological mean 850-hPa horizontal wind for each analysis period: 2006–2015 for the 10-year analysis and 1990–2019 for the 30-year analysis. The wind fields were calculated as the vector mean of the ERA5 zonal and meridional wind components and subsequently interpolated onto the 1.5° analysis grid.

Below, we present the corresponding ERA5-based analyses as a supplementary reference for assessing temporal variability (Figs S13-S23 and Table S2). ERA5 reanalysis should not be regarded as observational evidence, but rather as an independent long-term climatic reference used to assess the sensitivity of the results to longer analysis periods. GPM–GLEAM, being observation-based, remains the primary dataset in this study for defining the main aerial river drainage characteristics.

<table>
<tr><th></th><th>ERA5 10 years (2006-2015, matching the study period of manuscript)</th><th>ERA5 30 years (1990-2019)</th></tr>
<tr><td>Comparison with Fig. 2b</td><td>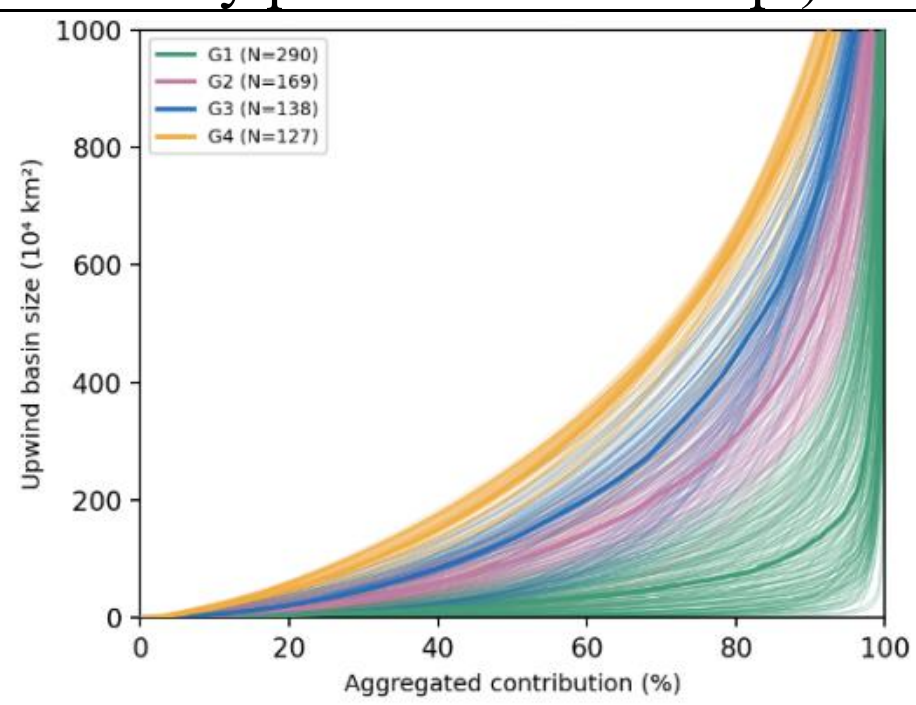

Fig. S13. ERA5 10 years comparison with Fig. 2b</td><td>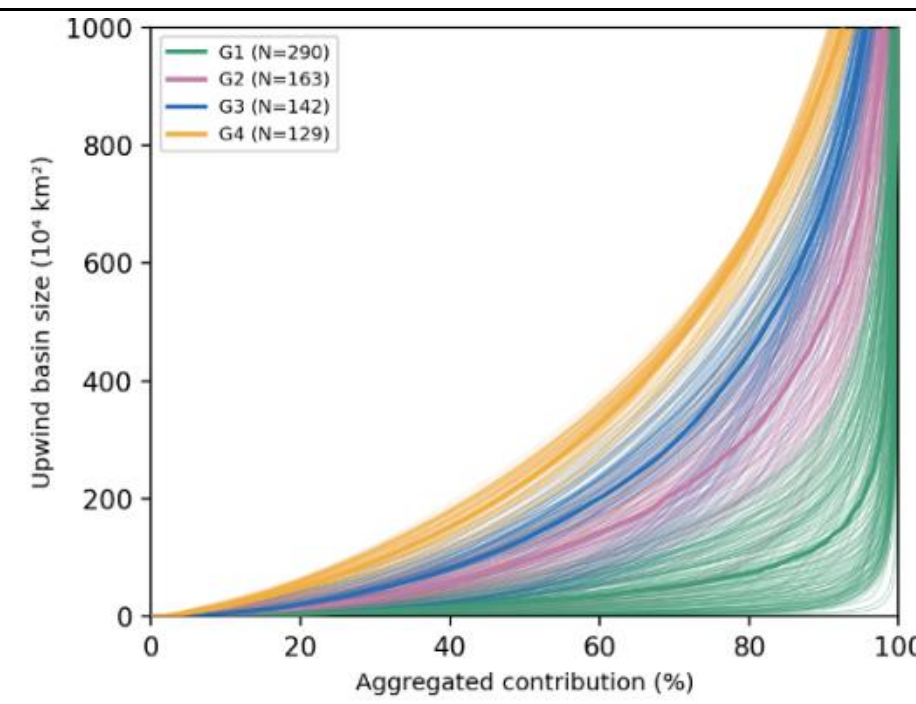

Fig. S14. ERA5 30 years comparison with Fig. 2b</td></tr>
<tr><td>Comparison with Fig. 3a</td><td>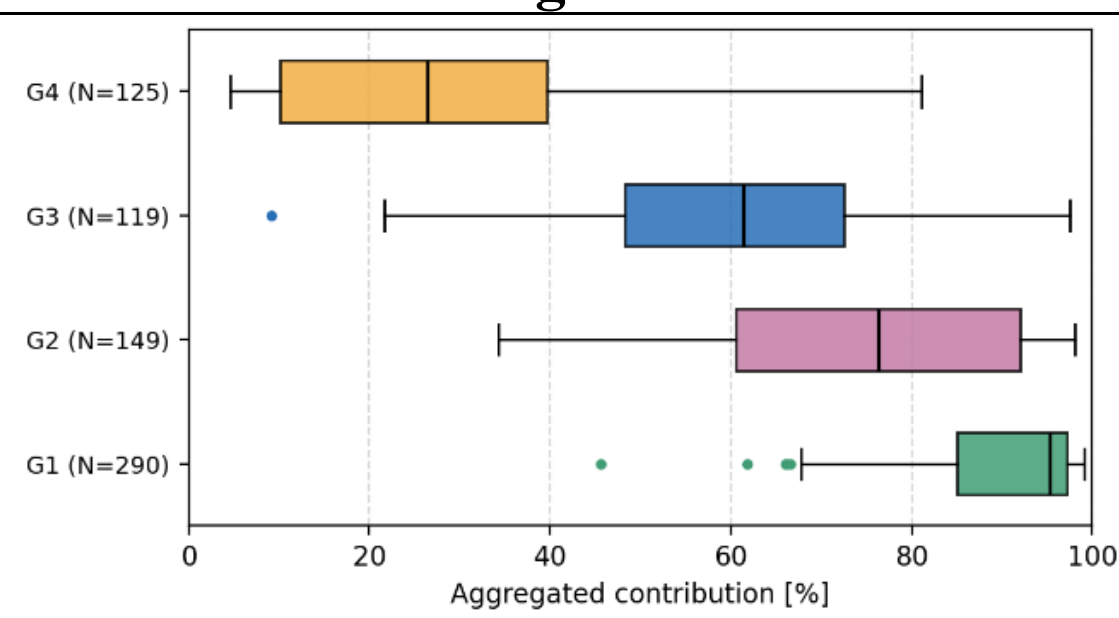

Fig. S15. ERA5 10 years comparison with Fig. 3a</td><td>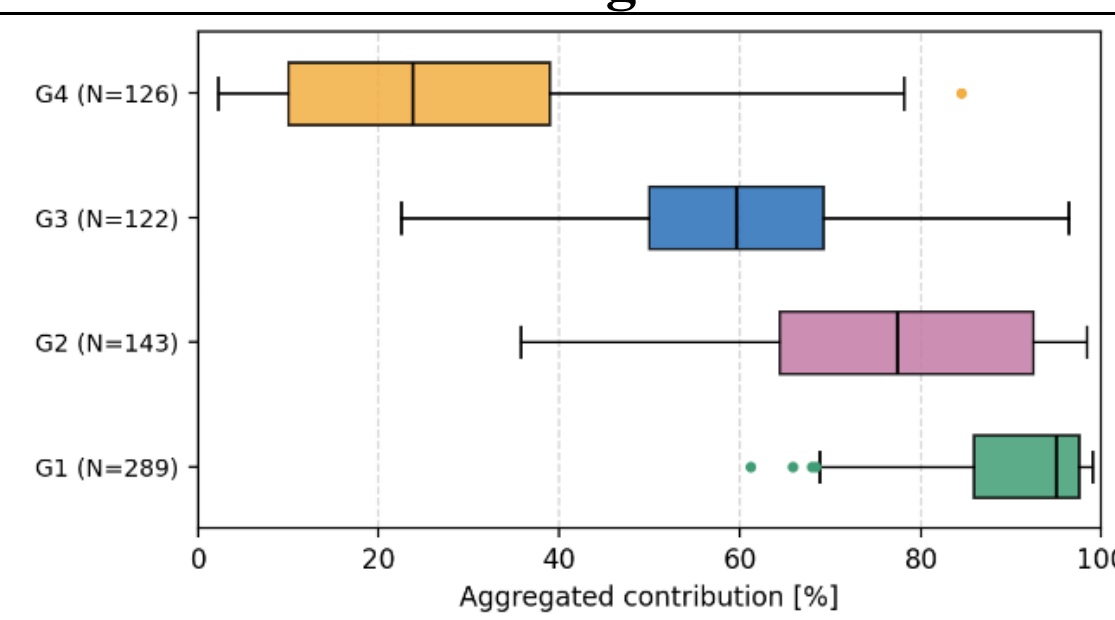

Fig. S16. ERA5 30 years comparison with Fig. 3a</td></tr>
<tr><td>Comparison with Fig. 3b</td><td>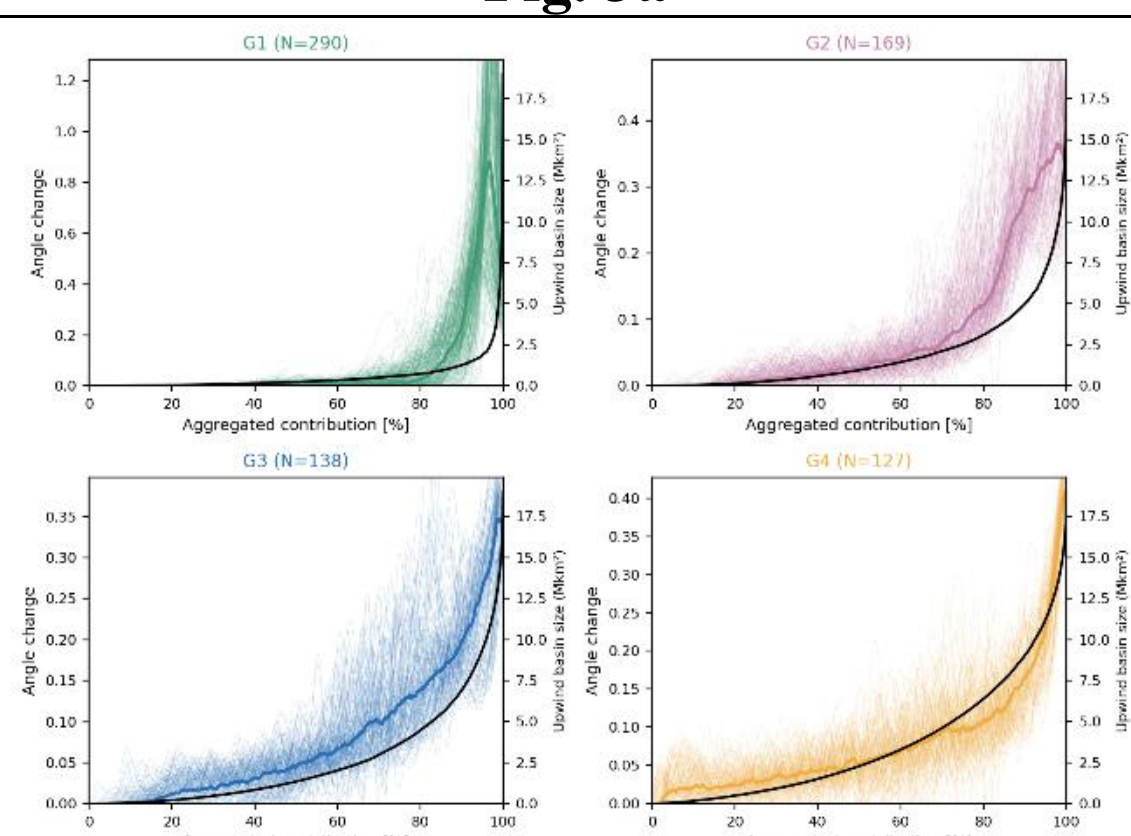

Fig. S17. ERA5 10 years comparison with Fig. 3b</td><td>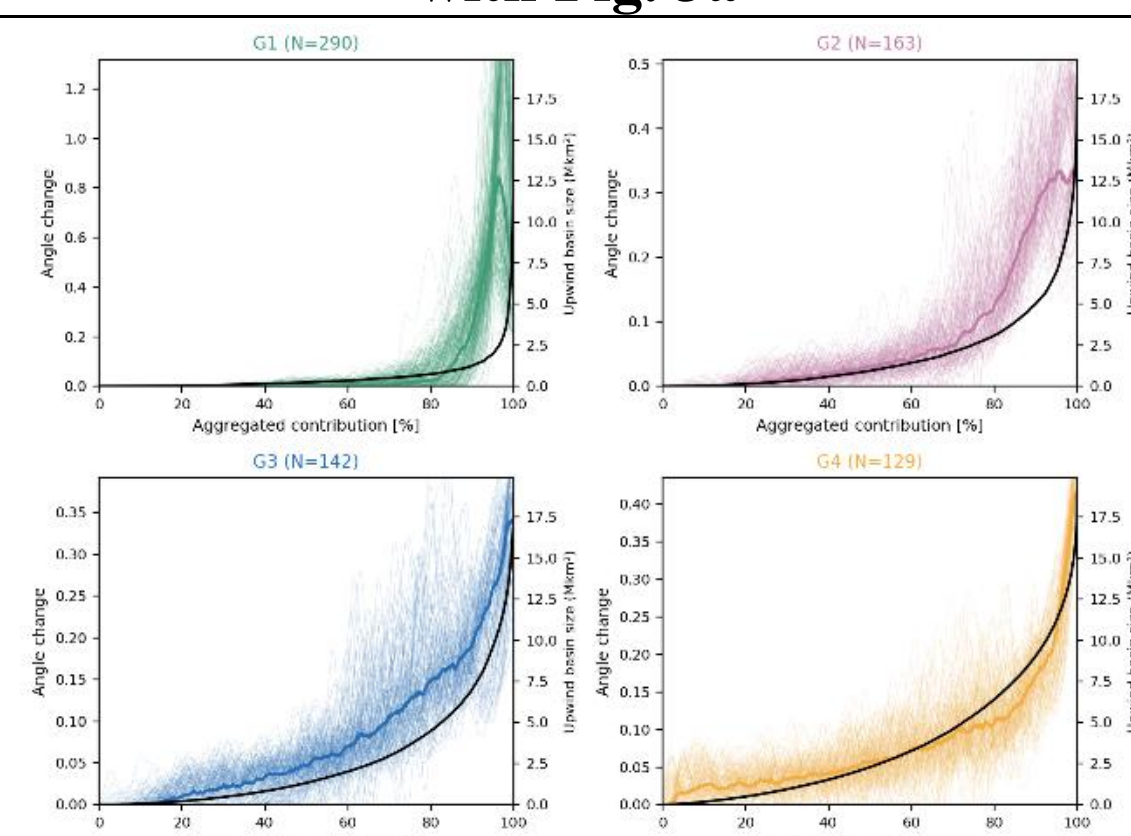

Fig. S18. ERA5 30 years comparison with Fig. 3b</td></tr>
</table>

Comparison with Fig.S5

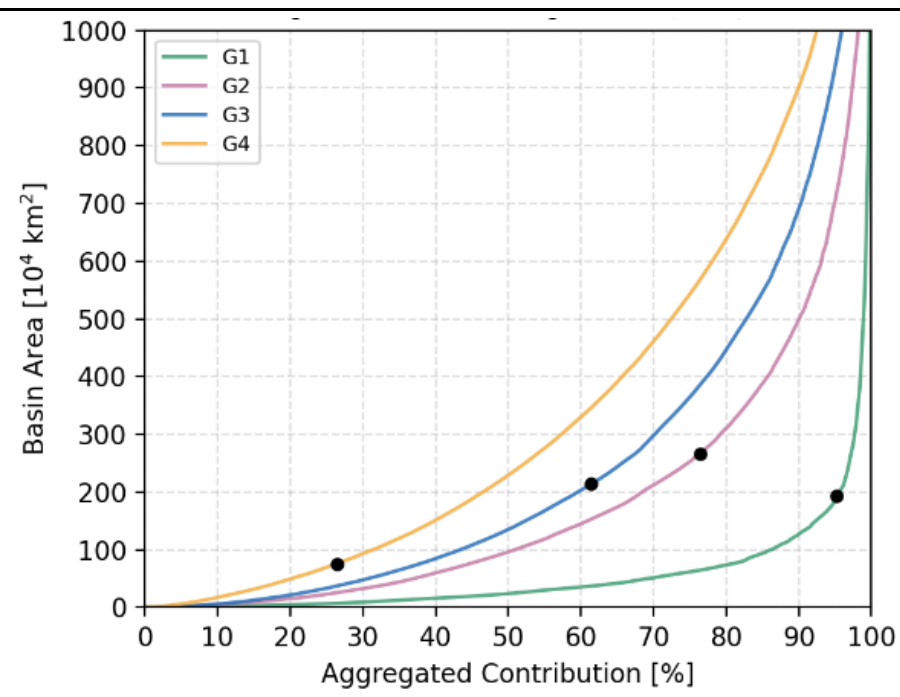

Fig. S19. ERA5 10 years comparison with Fig. S5

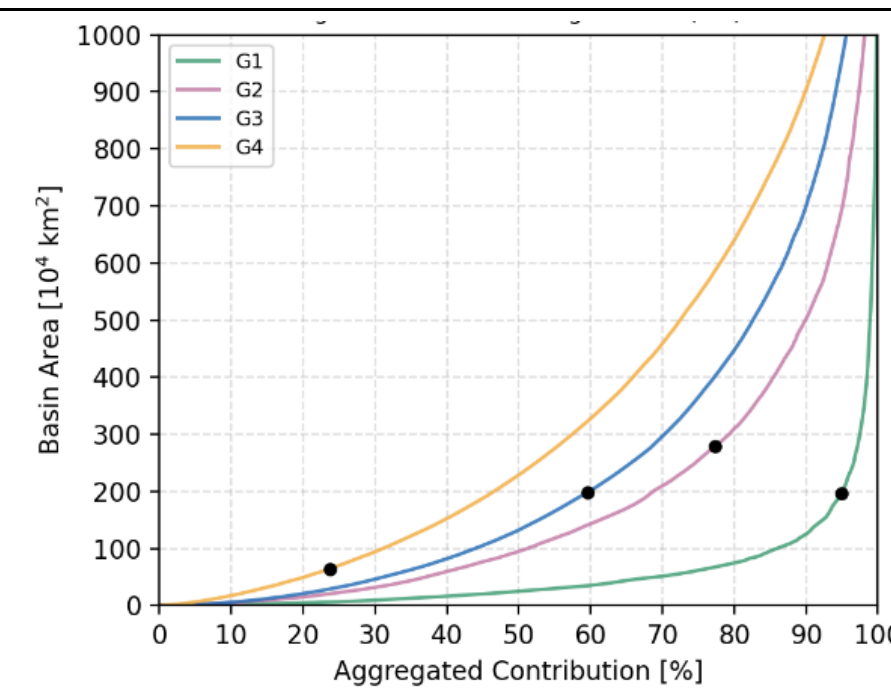

Fig. S20. ERA5 30 years comparison with Fig. S5

Comparison with Fig S3

ERA5 10 years (2006-2015, matching the study period of manuscript)

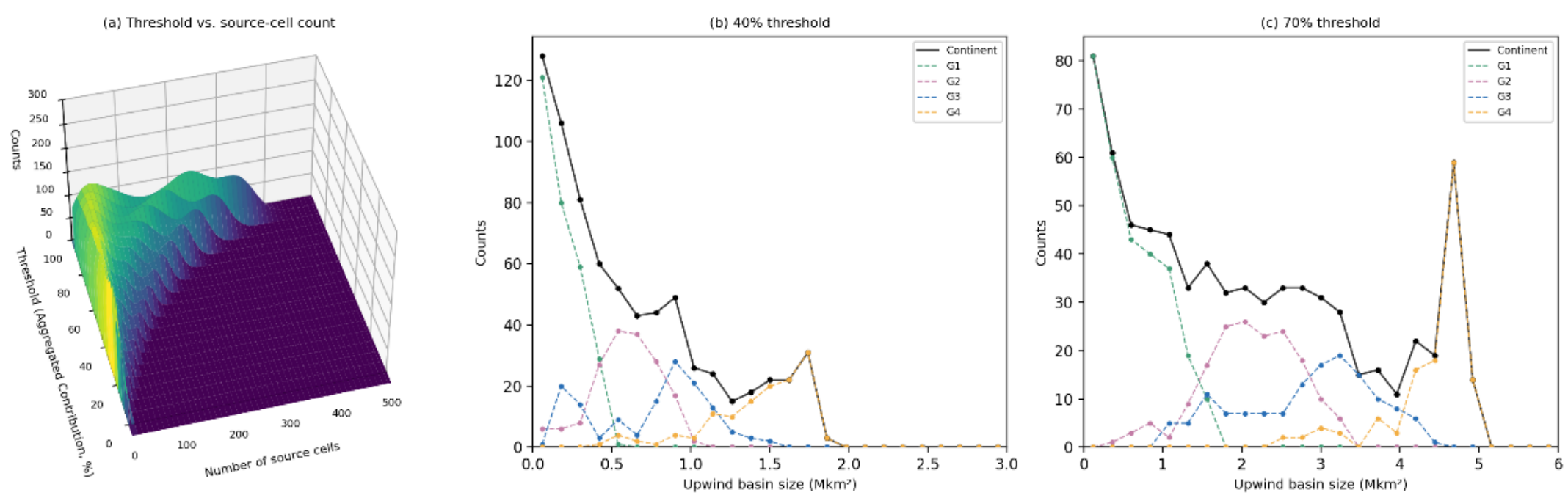

Fig. S21. ERA5 10 years comparison with Fig. S3

ERA5 30 years (1990-2019)

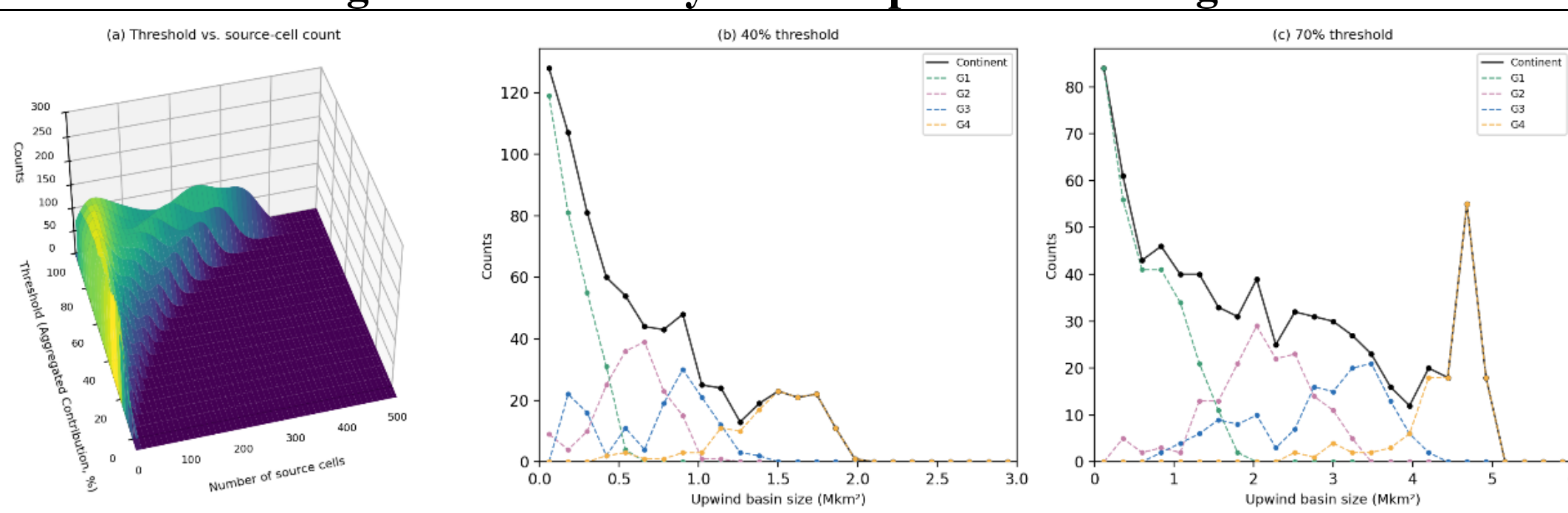

Fig. S22. ERA5 30 years comparison with Fig. S3

Comparison with Fig S8 for ERA5 30 years

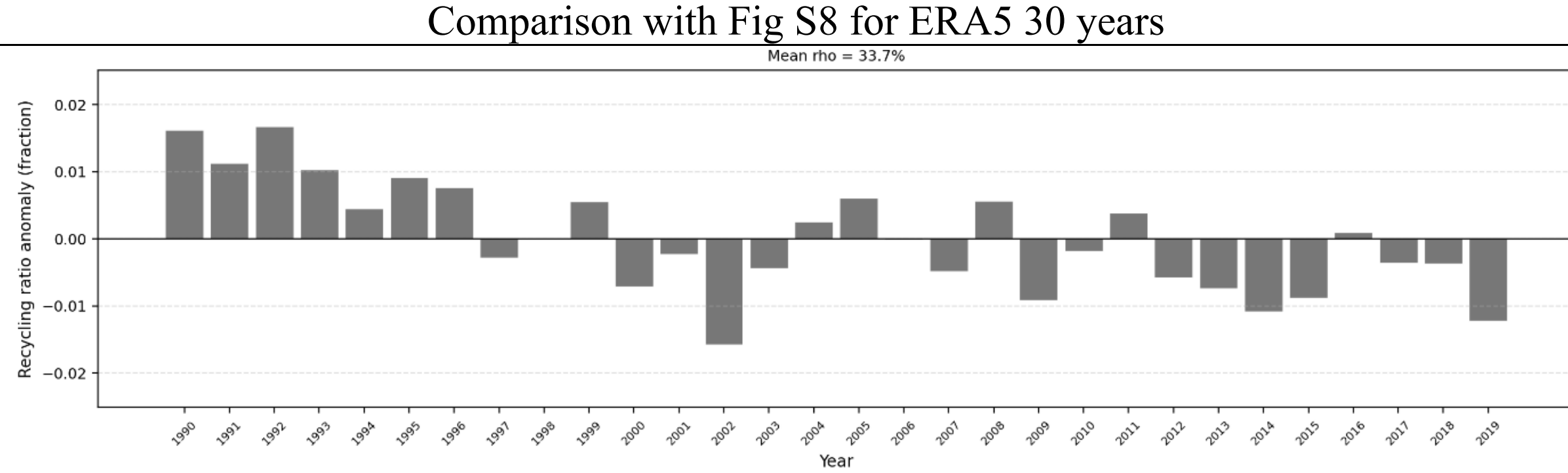

Fig. S23 ERA5 30 years comparison with Fig. S8

I. Bootstrap resampling for uncertainty estimation in drainage-type clustering.

We tested the robustness of our clustering results using the bootstrap method. This approach involves repeatedly resampling the data with replacement and recalculating the clustering for each resample. By comparing these repeated outcomes, we can assess how stable the clusters are: regions with consistently high probabilities are robust, while lower probabilities tend to occur near the boundaries between drainage types.

Each bootstrap dataset contained 724 moisture drainage curves of the South American continent, with individual curves potentially appearing multiple times within the same sampling round. The number of clusters was tested from 2 to 11 with 1000 bootstrap K-means runs for each. Based on the Calinski–Harabasz score, four clusters were optimal, though 5–6 clusters gave similar results (see Fig. S24).

For the 4-cluster scenario, 1000 bootstrap runs were performed, and each model classified the original moisture drainage curves. We computed (see Fig. S25):

- Majority class: the most frequent class across runs.
- Majority probability: the fraction of runs assigned to the majority class.

Most grid cells had a majority probability of 1, indicating stable classification, while lower probabilities (~0.6) occurred near cluster boundaries. Finally, because K-means assigns class labels randomly, we verified that the distributions of class membership across the 1000 runs were consistent. This consistency explains cases where the Majority Probability reached 1.

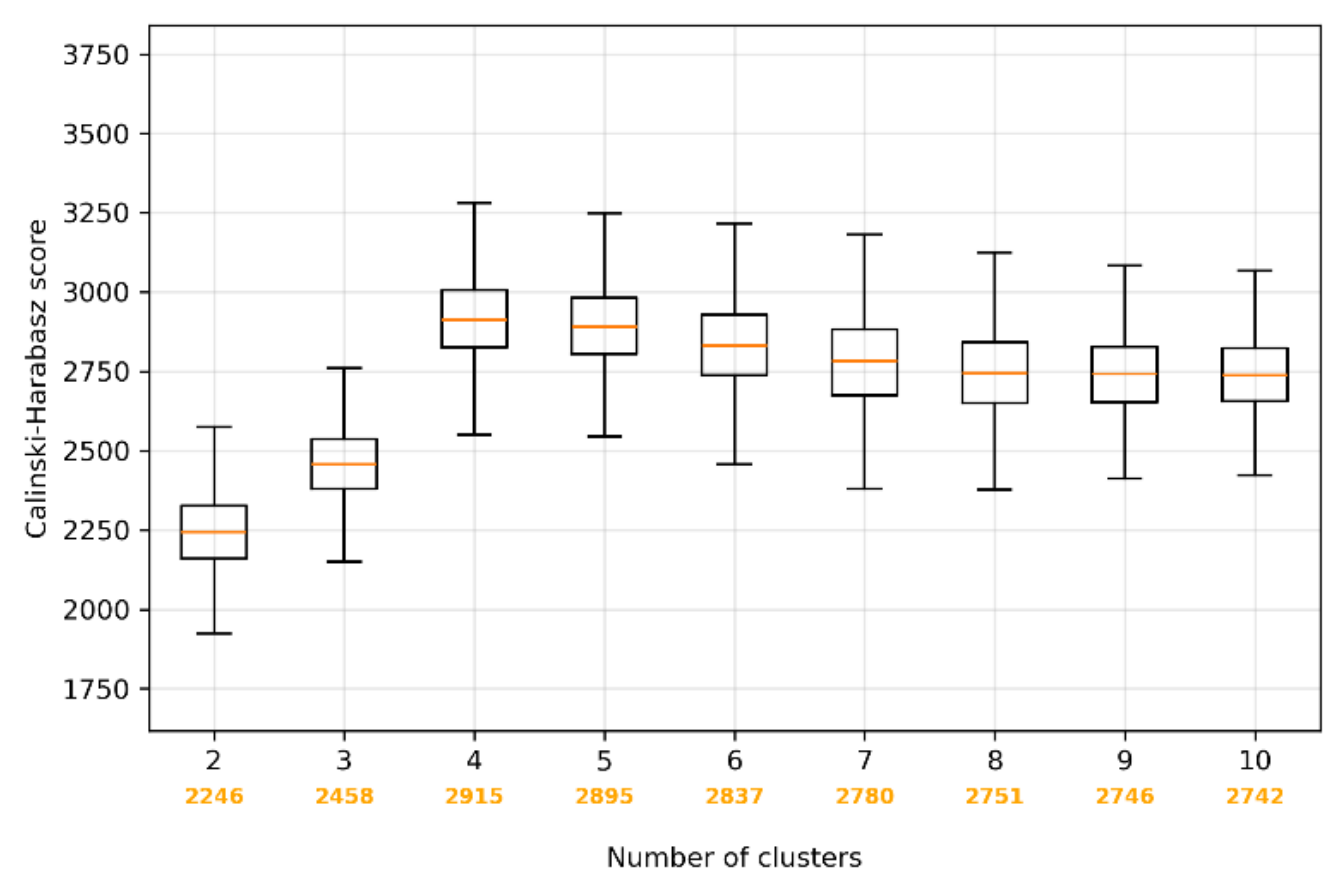

Fig. S24. Bootstrap Calinski–Harabasz score over 1000 runs. According to the Calinski–Harabasz score, the average performance was highest when the drainage curves were grouped into four categories, suggesting this as the optimal classification.

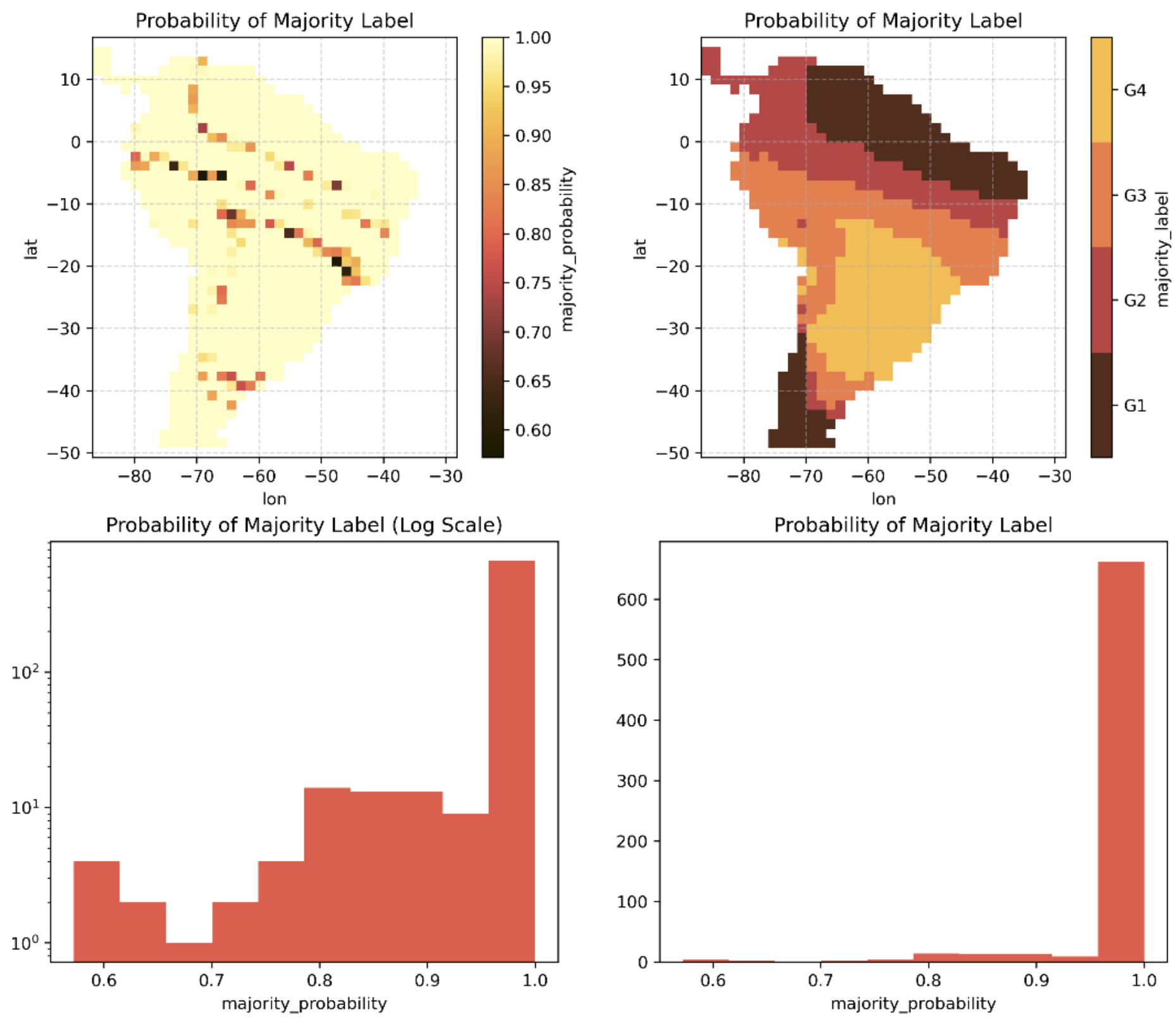

Fig. S25. Bootstrap majority class and probability over 1000 runs. Most grid cells had a majority probability of 1, indicating very stable classification. Some variability occurred near group boundaries, with minimum values around 0.6. The top-right panel shows that the distribution of the majority class aligns with the drainage type patterns.

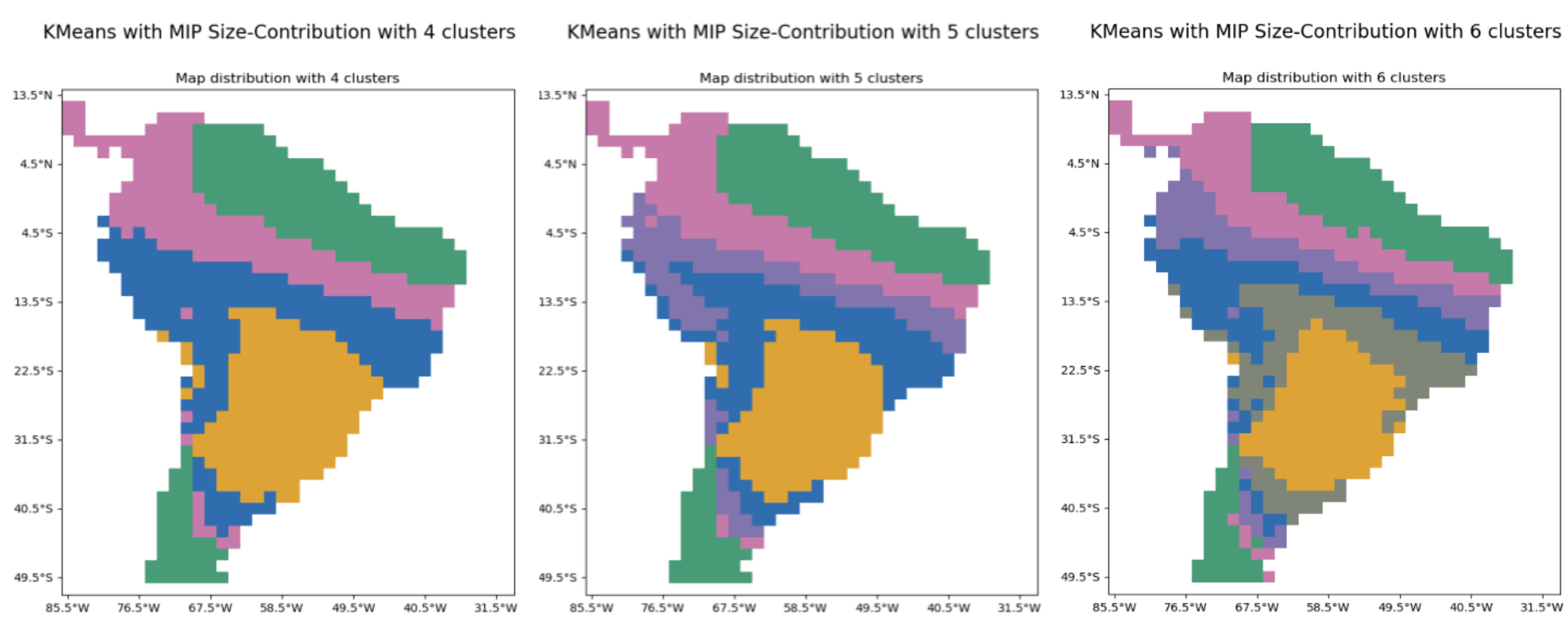

Fig. S26. More clusters reveal subdivisions, not new patterns. Four-cluster solution captures the primary structure of aerial river delivery dynamics, while higher cluster numbers mainly reflect finer subdivisions of existing groups. In particular, the additional clusters in the five- and six-cluster solutions largely represent finer partitioning of groups already identified in the four-cluster

configuration. A subdivision within G2, identified in the main text (see G2 Atmospheric drainage and Supplementary Fig. S4), emerges in the six-cluster solution. In both the five- and six-cluster solutions, an additional subdivision appears predominantly within G3, corresponding to differences in the local recycling ratio. One G3 subgroup exhibits a lower local recycling contribution, with moisture drainage curves that depart from the x-axis earlier and follow a steeper slope. However, because this study focuses on the moisture delivery pattern of aerial rivers, both subdivisions of G3 show similar behavior of the outfall region where wind speed slows abruptly. Therefore, we adopt the four-cluster solution, which better represents the primary structure relevant to aerial river delivery dynamics. Studies on local rainfall partition may benefit from exploring these finer subdivisions of the underlying mechanisms.

Table S1. Results of the difference test between moisture types.

P-values from a two-sided t-test for each variable. A "*" marks no significant difference was observed at P = 0.01.

	Continental recycling ratio	Water content	Moisture Traveling Time	Moisture Traveling Distance	Delivery intensity	Wind speed
G1/G2	5.66e-52	4.49e-09	1.72e-20	4.76e-25	0.070*	2.31e-31
G1/G3	2.78e-102	0.035*	4.33e-33	1.87e-22	9.78e-14	8.53e-67
G1/G4	8.71e-153	1.99e-05	1.12e-79	1.51e-54	9.36e-18	8.08e-70
G2/G3	3.12e-17	1.29e-13	8.19e-09	0.081*	2.30e-05	5.97e-15
G2/G4	3.43e-40	1.67e-19	1.08e-38	1.09e-23	7.76e-08	4.16e-14
G3/G4	1.51e-06	0.078*	1.86e-12	2.29e-17	2.55e-10	0.13*

Table S2. Comparison with Table 1 for ERA5 10 years (2006-2015).

	Grid cell counts	Continental recycling ratio	Water content [kg]	Moisture Traveling Time [hr]	Moisture Traveling Distance [km]	Delivery intensity [kg/m]	Wind speed [m/s]
G1	290	0.159±0.098	2.52e11±2.03e11	71.86±130.62	1099±910	2.95e05±3.24e05	5.77±2.25
G2	169	0.357±0.093	3.51e11±2.62e11	157.67±158.54	1935±1255	4.07e05±1.13e06	3.17±1.87
G3	138	0.506±0.091	2.77e11±2.24e11	294.63±217.19	2335±1564	2.10e05±2.73e05	1.82±1.11
G4	127	0.550±0.031	2.11e11±1.53e11	374.81±144.61	3581±1057	6.04e04±3.65e04	2.60±1.41

References:

1. Weng, W. et al. Aerial river management by smart cross-border reforestation. *Land Use Policy* **84**, 105–113 (2019).
2. Weng, W. et al. Hydrological regimes and drainage systems of aerial rivers across South America: Supplementary A. Zenodo https://doi.org/10.5281/zenodo.17547634 (2025).
3. Bastin, J.-F. et al. The global tree restoration potential. *Science* **365**, 76–79 (2019).
4. Isometric. Reforestation Protocol, version 1.2, section "Safeguarding of Water Resources". https://registry.isometric.com/protocol/reforestation/1.2 (2025). Accessed 12 July 2026.
5. World Resources Institute. Aqueduct Water Risk Atlas. https://www.wri.org/aqueduct (2023). Accessed 8 March 2026.
6. Müller Schmied, H. et al. The global water resources and use model WaterGAP v2.2d: model description and evaluation. *Geosci. Model Dev.* **14**, 1037–1079 (2021).

7. Weng, W., Luedeke, M. K. B., Zemp, D. C., Lakes, T. & Kropp, J. P. Aerial and surface rivers: downwind impacts on water availability from land use changes in Amazonia. *Hydrol. Earth Syst. Sci.* **22**, 911–927 (2018).
8. Hoek van Dijke, A. J. et al. Shifts in regional water availability due to global tree restoration. *Nat. Geosci.* **15**, 363–368 (2022).
9. Liu, H. et al. Intercomparison and evaluation of ten global ET products at site and basin scales. *J. Hydrol.* **617**, 128887 (2023).
10. Polasky, A. et al. Discrepancies in precipitation trends between observational and reanalysis datasets in the Amazon Basin. *Sci. Rep.* **15**, 7268 (2025).
11. Baker, J. C. A. et al. Evapotranspiration in the Amazon: spatial patterns, seasonality, and recent trends in observations, reanalysis, and climate models. *Hydrol. Earth Syst. Sci.* **25**, 2279–2300 (2021).
12. Lavers, D. A. et al. An evaluation of ERA5 precipitation for climate monitoring. *Q. J. R. Meteorol. Soc.* **148**, 3152–3165 (2022).
13. Hersbach, H. et al. The ERA5 global reanalysis. *Q. J. R. Meteorol. Soc.* **146**, 1999–2049 (2020).
14. van der Ent, R. J. et al. WAM2layers (v3.3.1). Zenodo https://doi.org/10.5281/zenodo.19135489 (2026).
