# Supplementary material for: Hydrological regimes and drainage systems of aerial rivers across South America
Source: Nat Commun. 2026 Aug 3;17:9341. doi: 10.1038/s41467-026-76303-y (PMC13529790; doi:10.1038/s41467-026-76303-y)
Supplement: Supplementary file 2 [file 41467_2026_76303_MOESM2_ESM.pdf]

nature portfolio

Peer Review File

Hydrological regimes and drainage systems of aerial rivers across South America

Corresponding Author: Professor Wei Weng

This file contains all reviewer reports in order by version, followed by all author rebuttals in order by version.

Version 1:

Reviewer comments:

Reviewer #1

(Remarks to the Author)
SUMMARY
The authors of this manuscript have investigated a new approach for classifying atmospheric moisture sources for the South American continent. Using a method to classify moisture sources based on a clustering of moisture backtracking analysis, he authors perform a comprehensive categorization scheme of moisture source regions. They then suggest management applications of their approach.

I think this work, if substantially revised, could be interesting. However, the current manuscript does not adequately explain why this classification is both worth digging into more deeply, and how it meaningfully is distinguished from other approaches. Notably this submission is to Nature Communications which requires a very high bar of both scientific novelty and clarity of presentation.

I recommend either comprehensive revision & resubmission of this manuscript, or rejection.

GENERAL COMMENTS
1. Distinguishing more clearly how the authors' work is new: Given this is a Nature Communications, there is a substantial expectation of novelty. The authors need to do a better job of crediting existing scholarship and highlighting where they have actually done something new.

2. Consistency in referencing: There are quite a few references that are not apparently correct. Given this, the authors need to very carefully review which papers are referenced, and for what.

3. Generalizability of findings: The South American continent is well-studied for moisture recycling, but has specific characteristics that render generalizability challenging at best. The presence of tropical moisture colliding with the Andes mountains is but one of the challenges for generalizability of something like moisture curves.

4. Utility of findings: The authors argue that past methods of delineation of moisture source boundaries are limited, incomplete, and/or inappropriate. However, the G1, G2, G3 and G4 definitions seem to be areas that receive progressively less moisture from the ocean, and progressively more from continental regions. This assertion is supported by the authors' own results, where they highlight "Aerial river management potential:" and then list the percent of total rainfall provided by the critical upwind basin. There is an intriguing difference between G2 and G3, apparently, but the authors do not dig into this enough.

5. Management discussion is incomplete: The authors are suggesting that their work provides a relevant insight for moisture management, yet they fail to comprehensively engage in the substantial literature that now exists on this subject. If the authors want to keep this discussion in their paper, they will need to much more substantially highlight how their work complements other approaches that have already been published (and the authors include many though not all those references), as well as highlight where their suggestions diverge from or advance beyond past approaches.

SPECIFIC COMMENTS
Page 2 Line 13. Do the authors mean topography? Altitude, at least on its own, is not what channels surface water. Topographic surface gradients, or something similar, would be a more appropriate phrase.

Page 2 Line 20. The reference of Keys et al. might be wrong here, since New Delhi is not apparently referenced in Ref #20. Please double check the referencing throughout.

Page 3 Line 12. As highlighted above the Authors will need to explain how their specific study can lead to more generalized findings.

Page 3 Line 19. I suggest choosing a single phrase to refer to the source regions, since having two names ("atmospheric basins" and "upwind regions") is not contributing to clarity.

Page 4 Line 1. The authors refer to G1 types as receiving a high percentage of terrestrial moisture from a small upwind basin. Is this simply because it's proximate to the coast? Most of the moisture falling in the G1 areas are coming from the ocean anyway, so this is an odd classification.

Page 6 Line 21. The authors are defining a new term ("head-moistureshed") for the flow of moisture when it transitions from ocean to continental areas. I would suggest that this is unnecessary and that the authors should simply call this "G1 Ocean to Continental regions."

Page 7 Line 7. In this paragraph, the authors use phrases like "shift left" and "shift upward", and it's not clear to what they are referring. Is this an XY plot? Is this on a map? I encourage the authors to be much more precise in their language here.

Page 8 Line 1. At this point, I am confused what critical upwind basin means in practical terms. The authors have defined the concept mathematically. I think the authors have something here, but it is not clear in the text. The fact that the length scale of moisture transport is similar in G2 and G3 seems very important, since the authors are claiming there is a substantial difference between G2 and G3. I encourage the authors to dig much more deeply here.

Page 8 Line 20. There has been quite a lot written already about what the authors are calling "moisture management". Yet there does not seem to be much explicit engagement with that literature. If the authors want to retain this section, the authors need to provide much more explicit engagement with the existing literature that discusses governance challenges. Moreover, an important author on this subject that has not been cited is Sofie te Wierik:
te Wierik et al. (2021) Reviewing the Impact of Land Use and Land-Use Change on Moisture Recycling and Precipitation Patterns. https://doi.org/10.1029/2020WR029234

te Wierik et al. (2019) The need for green and atmospheric water governance. https://doi.org/10.1002/wat2.1406

Page 13 Line 18. The authors claim that their use of annual flows of moisture is sufficient to build a picture of aerial rivers. But what about interannual variability? The authors need to clarify how a relatively short record (~10 years) is sufficient to eliminate multi-year modes of variability from their analysis. Or, acknowledge the presence of these modes, and discuss the implications of this variability in terms of their findings. They do so with regard to mega-droughts. The same ought to be done for other modes of large scale climate variability.

Page 13 Line 24. I encourage the authors to share the mathematical basis of their calculations. The key equations would be helpful here. At present, the methodological approach for calculating the turning points is insufficient.

Page 13 Line 35. This is an example of where the authors go into great detail about the k-means operation they perform (a very standard statistical method). A similar level of detail would be useful for the turning points mentioned above.

Page 14 Line 14. Did the authors construct their own point tracking algorithm "based on the Lagrangian specification of motion." If so, that's very interesting. However, it would be useful for the authors to explicit state that.

Page 14 Line 21. How were residence times calculated? The trajectory of the parcel is straightforward based on the description, however the residence time is often prescribed in Lagrangian calculations. More explanation is needed.

Reviewer #2

(Remarks to the Author)
Review of the paper entitled: Aerial river drainage: Hydrological boundaries from long-term continental moisture flows

General comments:

I found the paper interesting and innovative but also confusing due to the terms used for water in the atmosphere, such as

aerial rivers, atmospheric rivers, flying rivers, and low-level jets (as mentioned in some of the listed references). Are they the same? If not, what is the difference between them? In any case, while comparing surface rivers and aerial rivers in general terms may make some hydrological sense, using surface rivers in planning and management may be more sensible than using aerial rivers, which are derived from reanalysis (ERA-interim) and whose results may change if other reanalysis products are used, making these calculations uncertain. In sum, there are limits on what the results can be used for, but I do not believe they could be used for the management of water resources in geographic regions or even to discuss tipping points.

Therefore, I believe that major reviews are necessary.

Specific comments:

Page 1:
Line 14: it is a bit unnatural to define boundaries to atmospheric flow; nature does not work this way.
Line 30: Please discuss the differences between atmospheric rivers and aerial rivers, as well as low-level jets.
Line 32:... and also from moisture transport from the winds, that in the Amazon are the trade winds from the tropical Atlantic. Atmospheric rivers are not formed in the Amazon region; they originate from outside the Amazon.
Page 2:
Line 8: where are the aerial rivers born? In continental regions, are they fed by evapotranspiration only?
Page 3:
Line 6: why Santa Cruz de la Sierra?
Page 4:
Line 7: which regions show the contribution of moisture from the Atlantic? The Atlantic is also a source of moisture.
Line 25: again, where are the sources of moisture and rainfall? Are they all on the continent? What about the Atlantic and Pacific Oceans?
Page 6:
Line 10: each region shows a different type of vegetation, how the evapotranspiration of different vegetation types is included>, for instance, over the Amazon forest or the Cerrado region in west central Brazil
Table 1, second column: again, what is the role of vegetation type in each region? How is the continental recycling ratio calculated?
Line 19: I do not see this clearly; contrary to surface rivers, where one can delineate the basin and measure the flow, aerial rivers are more abstract, and calculations are more based on assumptions that may change if one changes the atmospheric data sets used for the calculations. How can one use this data for environmental policies (forest restoration, etc.)? The aerial river calculations are subject to uncertainties, which may be much higher than measures in surface rivers.
Line 30: does this suggest that 11-12% of moisture comes from the ocean and the rest is from continental origin? Does this mean that 11-12% of the moisture comes from the sea, and the rest is from continental origin?
Page 7:
Line 2: this number., as well as from the other G regions, needs to be better explained
Line 30: River management, surface river management? How can we manage something we do not see when measurements show uncertainties?
Page 8:
Line 29: The mathematical analysis is good; it may show uncertainties but seems to be good; now, the aerial rivers are not surface rivers where you can see the water and the basin and measure the flow. You can use the surface river data for water resources management, but doing this management with something more abstract, such as aerial rivers, I am not sure if this is the way to go.
Page 9:
Line 13: You can use the surface river data for water resources management, but doing this management with something more abstract, such as aerial rivers, I am not sure if this is the way to go.
Line 15: I am not sure if we have reached that level of refinement in managing the water rivers; this is an abstraction with uncertainties.
Line 26: I believe that the authors are expecting too much from these results.
Page 10:
Line 8: how much manageable is possible?
Line 14: I am sorry, but I do not see that
Line 16: this is a good example of connectivity,
Page 11:
Line 4: what does this mean?
Line 13: This kind of figure (Figure 5) was previously shown in studies considering the low-level jet east of the Andes in South America. In the figure, it appears that flying rivets are present, and the paper discusses aerial rivers and atmospheric rivers. Are all of those the same?, the components of the South American monsoon system?
Line 20: I am unsure if this term can be used in this paper, as the objective is not to define whether we have reached or even if we have defined a tipping point. I do not think that, based on the evidence presented in this paper, we can discuss reaching or surpassing a tipping point (ecological, climate-social).

Reviewer #3

(Remarks to the Author)
Summary:
This paper classified the South American region into four categories based on the moisture drainage curve. The moisture

drainage curves were generated based on the moisture tracking results, focusing on a climatological scale with the concept of "aerial rivers". The study further examined the derivative of the curve angle to quantify the critical upwind basin size for each region. Such a characterization analysis was also conducted in the aspect of aerial rivers (i.e., physical characteristics of aerial rivers). Finally, the study discussed the potential of applying the proposed framework for water resource management in the target region.

Overall assessment:
I believe the concept of defining hydrological boundaries based on the critical upwind source region, rather than the conventional watershed boundary or municipal boundary, is interesting.
The regional difference in classification is worthwhile investigating to better understand the water source of such a region based on the provision of atmospheric moisture. Such understanding would be useful for future water resource management in the region.
However, I have major concerns about the difficulty of interpreting the results, the uncertainty with the employed methods, the novelty of the study, and the insufficiency of the proposed framework.
I have some moderate comments, which I believe are all essential to enhance the value of this paper.
Please find my comments below:

Major
-Difficult interpretability of the results:
It was quite hard to physically/geographically interpret the findings like the ones shown in Figure 2. In that sense, I found Figure S5 extremely useful to understand the actual extent of the inland upwind basin. My suggestion about showing the dominant wind pattern is also relevant.
I suggest the authors incorporate the figures showing the actual extents of the critical upwind basins as well as the dominant wind pattern (you can test and see), ideally for G1-G4 (maybe one or two for each region?).

-Uncertainty in the employed method:
I see the authors' statement about the significant sensitivity of the fixed threshold to determining the classification. I believe that such sensitivity also applies to the clustering method (also the moisture tracking method). Quantifying the uncertainty associated with the method would be important. This is also because the authors suggested the framework in Figure 4 in a general way in which the readers have some freedom to select the methodology in clustering and moisture tracking. Performing several methodologies may not be realistic, but could the authors at least somehow quantify the uncertainty rooted in the methodology? Please consider.

-Novelty of the study:
I do not clearly see the novelty of this study. It reads as just applying the existing methodology to the continental scale. I suggest re-structuring the introduction and discussion based on the following points:
- The authors need to (more) clarify the novelty in the methodology if there is any.
- The authors need to better structure the problem statement, which ideally is connected to the novelty statement.

-P9 Lines 3-26 and Figure 4:
I have major concerns about the insufficiency of the suggested framework regarding the points below.
- The practice of the author's proposal (e.g., reforestation) for a region (let's say Sorriso in G3) should affect the downstream. Thus, it seems to need an optimization for multiple regions. This may apply not only to the downstream close by, but also to much larger regions. I understand that Figure 4 is just a suggested framework. However, if the authors insist on suggesting an actual framework in this high-impact journal, the authors should be mindful of the potential impact of the suggested plan. Does it not reduce the downstream water availability? Unless the authors show a scientifically/socially reasonable argument, I do not believe it is ready to suggest as a framework. In fact, this may work as a maladaptation. Please explain/revise.
- As being one Figure to be impactful enough, I believe the figure needs to be not only conceptual but also show the actual example. Why don't the authors consider integrating Figure 4 and Figure S5? Please consider.

Moderate
p1 Lines 29-30:
The sentence does not sound scientifically appropriate. I believe "aerial rivers" themselves represent climatology, not "like Earth's climate". The authors may consider phrasing "Aerial rivers are often described as climatology expressed through the persistent flow of atmospheric moisture over time." or anything similar.

P2 Line 1:
"[…] decrease in annual rainfall and a 19-50% decrease in annual runoff […]"
Did the authors mean a decrease in the downstream? Please specify.

P2 Lines 1-3:
The sentence about Peruvian Ucayali and the one about the more general can be combined.

P2 Lines 5-6:
"are most likely inadequate"
"So, what should a water management system integrating aerial rivers be based on?"
The issue the authors raise here is vague. The authors need to be more specific about the issue in the gap between aerial river coverage and watershed/political boundaries. What do you mean by inadequate? In what sense is it inadequate? Why

is it an issue not to consider aerial rivers in the current water management system?
Better clarification of the research gap and issue here is critical for highlighting the value of this paper.

P3 Line 2:
Here, the research question seems to be replaced with “the need to explore an objective standard for identifying upwind regions”, which is not necessarily straightforwardly consistent with the water management question (see the comment above). The authors need to clarify what the research question is and better structure the introduction.

Figure 2
It is interesting to examine the large variability in Chile and Argentina, which makes sense based on their geographical shape.
For instance, the central to southern Chile and Argentina where heavy precipitation is dominated by ARs (Viale et al., 2018; Hiraga and Meza, 2025), is G1. Can we say a major portion of impactful “aerial rivers” are “ARs” in this region? Meanwhile, the northern Chile and southern Peru where convective systems occur (Huang et al., 2024) are classified as G3/G4.
I believe this shows the climatology rather than the extremes, but the regional classification seems to be somewhat related to the dominant storm types. I suggest the authors discuss the results in the context of such storm types.
References:
- Viale, M., Valenzuela, R., Garreaud, R. D., & Ralph, F. M. (2018). Impacts of atmospheric rivers on precipitation in southern South America. Journal of Hydrometeorology, 19(10), 1671-1687.
- Hiraga, Y., & Meza, J. (2025). Extreme precipitation modeling and Probable Maximum Precipitation (PMP) estimation in Chile. Journal of Hydrology: Regional Studies, 58, 102274.
- Huang, Y., Xue, M., Hu, X. M., Martin, E., Novoa, H. M., McPherson, R. A., ... & Flores Luna, A. J. (2024). Characteristics of precipitation and mesoscale convective systems over the Peruvian Central Andes in multi 5-year convection-permitting simulations. Journal of Geophysical Research: Atmospheres, 129(17), e2023JD040394.

Figure 3
I believe the box plots show the variability of the peaks. If so, the authors should mention it in the caption. Also, peaks seem not to be always prominent, especially for G3. It is objective to always select a single peak, but the authors may need to ignore the second or third peaks even if they are very close to the first peak. I believe the treatment of the peaks may affect the distribution summarized in Figure 3a. The authors can provide a discussion on this issue if the authors think it is significant.

p4 Lines 8-25:
The authors discuss the results shown in Figure 3 based on the upwind basin size. Then why did the authors not summarize Figure 3 based on the upwind basin size, not the aggregated contribution? Isn’t it more straightforward? Please explain/consider.

P7 Lines 5-6:
I believe showing the climatological wind pattern (maybe at 850hPa?) over the region would be highly effective for a better physical interpretation of the results. This applies to not only here.

P11 Lines 19-28:
I wonder whether the authors should discuss the ecological tipping point only based on the G1-G4 category. This should be strongly related to the vegetation types, including natural or human-managed. Although the point raised is important and worth mentioning, it is necessary to acknowledge such limitations for discussing the ecological tipping point.

P13 Line 6
As the authors mentioned, aerial rivers should be defined based on climatology.
With that said, the time period the authors focused on seems to be too short to define the climatology.
The authors need to quantify the impact of the selected time period on the results to enhance the robustness of the findings.

Version 2:

Reviewer comments:

Reviewer #1

(Remarks to the Author)
The authors have submitted a revised version of their manuscript. In review #1, I suggested either "comprehensive revision and resubmission", or "rejection". The authors were given the opportunity to revise their submission.

While the authors have made some changes, many of my original comments were not actually addressed.
I recommend Rejection and provide these final comments.

1. The results are not long-term. The authors use the phrase "long-term" in the title, and throughout their text. However, 10 years of analysis is not consistent with a long-term analysis, let alone for the climate system. 10 years would be appropriate for shorter-term exploratory analyses. However, the authors are aiming to provide management-relevant science, and make mathematical claims about the behavior of atmospheric water transport. It would be challenging at best to provide insights relevant to water management with only 10 years of analysis. Multiple decades (at a minimum) is likely required to capture

enough variability to support management-relevant findings.

I will note that the Limitations section (which acknowledges the issue of a short duration) is at the end of Methods in this current revised manuscript. Nature Communications typically puts Methods at the end. So, in practice, the authors have put their Limitations section at the end of the manuscript, after all the other Methods. If the authors resubmit this work elsewhere, I suggest that they put such a limitations section in the Discussion.

2. Not the state of the art. Given that this is Nature Communications, it is reasonable to expect that cutting edge data and tools be used. While the authors use multiple datasets (including IMERG and GLEAM), they are also using ERA-Interim data for a period of 10 years. A cutting edge analysis would reflect the latest in atmospheric water tracking procedures. Given the authors use the WAM2-layers, that could mean using ERA5 reanalysis data (the current state-of-the-art reanalysis data product) for at least 30 years of analysis. The authors are aiming to publish their work in a highly prestigious journal, so I am deliberately holding this work to a high standard.

3. Insufficient engagement with the management literature. In the first set of comments, I suggested the authors needed to "much more substantially highlight how their work complements other approaches that have already been published", as well as "highlight where their suggestions diverge from or advance beyond past approaches." It is frustrating that the authors did not do this. The authors make claims about their own work and then reference previous studies. But in this revised manuscript the authors, again, fail to actually discuss how their work complements what has already been written over the past decade (at least) of atmospheric water management research.

The paragraph on page 18, Line 3 of the revised manuscript is a perfect example of inadequate claims of novelty. The second sentence of this paragraph reads: "Managing aerial rivers at the continental scale necessitates a reconceptualization of the governable dimensions of hydrology and a redefinition of the critical zones where water resource conservation intersects with land-use decisions."

This is written as an original thought (and it very well may be). However, the same concept has been written about many times by others over the past ten years at least. Currently, a reader of the author's Revised manuscript could easily draw the conclusion that very little if anything has been written about these subjects, while the opposite is true. Indeed, much has been written about atmospheric water management, precipitation recycling management, upwind source region management, and even aerial river management. The authors cite some of this work, but they did not "substantially highlight how their work complements other approaches that have already been published", and they did not "highlight where their suggestions diverge from or advance beyond past approaches."

(Remarks on code availability)

Reviewer #2

(Remarks to the Author)
I believe that the authors have responded to my comments and suggestions in a satisfactory way. Therefore, I suggest acceptance of the paper.

(Remarks on code availability)
I believe that the authors have responded to my comments and suggestions in a satisfactory way. Therefore, I suggest acceptance of the paper.

Reviewer #3

(Remarks to the Author)
I would like to thank the authors for their thoughtful and comprehensive responses. The manuscript has certainly improved, particularly in terms of clarity and structure. That said, I believe there are still a few areas that would benefit from further clarification or development, especially to strengthen the scientific foundation and practical applicability of the study, which are outlined below:

Methodological Uncertainty
I appreciate the authors' efforts in conducting a bootstrap analysis for quantifying the uncertainty. It is now clear that the selection of four clusters was not significantly optimal, since five or six clusters show similar performance. Based on these results, I believe the authors need a stronger justification for focusing only on the four-cluster results.
Can the authors explain the similarity of cluster divisions in the cases of four, five, and six clusters based on physical mechanisms, such as climatological moisture flow patterns? Such additional analysis is necessary to increase the robustness of the findings presented in the paper based on the four-cluster results.

Potential Trade-offs in the Framework
I appreciate the addition of discussion around the potential downstream impacts of interventions like reforestation. However, the current framing still feels somewhat conceptual, and readers may find it difficult to evaluate how this framework might perform in a real-world context.
Would it be possible to include a simple illustrative example showing how trade-offs between upstream and downstream

regions could be assessed using your framework? This doesn’t have to be a full model—just a basic water balance sketch or reference to existing studies could help ground the discussion.

Time Period and Climatological Representation
It would be helpful to discuss more explicitly how this limitation might affect the results. For example, do the classifications hold steady across different sub-periods? If longer datasets (e.g., reanalysis products) suggest similar patterns, even a mention of that would help put the current results into context.

Peak Selection
The added explanation of peak selection criteria is helpful. However, since some curves are multi-peaked, I wonder how sensitive the analysis is to this prominence threshold. Would varying the threshold (e.g., from 0.05 to 0.03) significantly change the distribution or classification?

(Remarks on code availability)

Version 3:

Reviewer comments:

Reviewer #1

(Remarks to the Author)
The authors have made many new additions to their analysis to respond to previous reviewer comments, something that is very helpful toward evaluating the robustness of the different clusters, and ultimately for evaluating whether the claims are supportable related to the robustness of the different types of upwind aerial basins. This is good progress. I have a final revision request that I describe below. I also want to reiterate that I am holding the authors to a high standard here because this is for Nature Communications.

The authors suggest that their contribution is centrally methodological, and their statements throughout their work (including the first two sentences of their final Maintext paragraph) point to the fact that they reveal positions that reflect long-term planetary moisture transport:
"The moisture drainage types we found here are geographic divisions shaped by long-term planetary atmospheric moisture transport which is relevant for regional water resources. They correspond to fluvial positions within the continental aerial river system: headwater, drainage, outfall, and plain."

This is not me shifting the focus away from the author's intended contribution; The authors state their intended contribution very clearly in this final paragraph.

That being said, I think there is an easy/straightforward resolution to all of this: Provide clearer more comprehensive information about the long-term WAM2layers analysis the authors performed using ERA5.

The authors note that they have already completed this 30 yr analysis, but only show a single figure in the Supplement (Supp Fig S11). They provide almost no other detail about this (considerable) additional work. 30 years of ERA5 analysis run through the WAM2layers is a substantial undertaking, and there is much that could be done with this analysis to support their claim of long-term relevance.

I would like to see a full suite of sensitivity analysis figures to complement the central findings of the GLEAM-GPM data. Specifically, provide the following for the 30 year ERA5 WAM2layers runs (which you've already produced for the GLEAM-GPM analysis):
1. Maintext Fig 2b, 3a and 3b, Table 1, and
2. Supplemental Figs S3, S5, and S7.
These could all go in the Supplement (which doesn't cost anything in word count, yet will build much more trust with a skeptical reader).

I would recommend (though its up to the authors) to include a comparison figure in the maintext which would give the average reader of the article (who doesn't dive into the supplement) a chance to see the good agreement between the long-term dataset (ERA5) and the GPM-GLEAM dataset the authors choose to feature.

To sum up: The authors have are almost there. The final step is to show comprehensively that their 10 year results (from GPM-GLEAM) align closely with the 30-year results that are actually at the time scale of long-term atmospheric behavior. Making these figures should be a small amount of work for the authors since they have already completed the ERA5-based WAM2layers analysis. And yet, these figures will disproportionately strengthen their work by providing the fundamental evidence that their concluding paragraph claims to find.

If the authors do this, I will be happy to see this contribution published.

(Remarks on code availability)

Reviewer #3

(Remarks to the Author)
This manuscript addresses a critical methodological challenge in water management: how to define upwind moisture source regions objectively without relying on arbitrary fixed thresholds. The core concept of critical upwind basins is originally proposed by the authors and has significant potential to improve land-water management and moisture governance under a changing climate.

However, I have the following major concerns remaining:

#1
In Table 1 and the Methods section, the authors define "Moisture transport efficiency" by dividing the total moisture content by the distance traveled. However, physically, this represents a spatial derivative or a path-averaged line density, not an "efficiency" in the standard hydrological sense (which is usually a dimensionless ratio of output to input, or a measure of mass loss). The authors should clarify why this specific metric represents efficiency, or rename the variable to better match its physical meaning.

#2
Methods state that moisture content is calculated as the sum of the moisture load along the pathway. If this is a simple summation of values sampled at 3-hour intervals, the total value will artificially change depending on the sampling frequency and resolution. The authors need to clarify the exact mathematical formulation (e.g., whether it is a path integral) and prove that the metric is independent of model resolution.

#3
In the Discussion section, the authors argue that deforestation per unit area in the upwind basins of G1 and G2 will trigger Amazonian forest dieback more easily than in G3 or G4. They base this claim entirely on the high delivery efficiency per unit area shown by the sharpness of the G1/G2 drainage curves.
However, this conclusion overlooks the absolute moisture dependency of the receiving regions: According to Table 1, G1 regions rely almost entirely on ocean sources, and continental-sourced rainfall accounts for only 12% of their total precipitation. Thus, even if its critical upwind basin is completely deforested, the maximum potential loss of total rainfall in G1 is limited to about 11%. In contrast, G4 regions depend heavily on continental land sources, with a continental recycling ratio of 49%.
While G1 and G2 upwind basins show higher sensitivity per unit area, G4 is far more vulnerable in terms of the absolute water volume at risk. The claim that the tipping point threshold is lower for G1/G2 clearing is a logical leap. The authors must revise the discussion to balance per-area sensitivity with absolute hydrological vulnerability.

#4
Under "Deriving moisture drainage curves" in the Methods, the authors state that the drainage curves were transformed to natural logarithms, interpolated into 1,000 evenly spaced intervals, and then converted back to the original scale using an exponential function before calculating the local curve angles and derivatives.
Transforming a highly non-linear, exponential-like curve into log-space, performing interpolation, and then transforming it back via an exponential function can mathematically alter the local curvature (the second derivative).
This pre-processing step might introduce artificial smoothing or sharpening at the critical bend of the curve. The authors need to demonstrate whether the identified turning points change significantly if the interpolation and derivative calculations are performed directly on the raw, non-log-transformed data.

(Remarks on code availability)

Version 4:

Reviewer comments:

Reviewer #1

(Remarks to the Author)
Thank you. The authors have responded to all of my comments.

(Remarks on code availability)

Reviewer #3

(Remarks to the Author)

I appreciate the authors addressing my comments, which I beleive significantly improved the quality of the paper.

(Remarks on code availability)

Open Access This Peer Review File is licensed under a Creative Commons Attribution 4.0 International License, which permits use, sharing, adaptation, distribution and reproduction in any medium or format, as long as you give appropriate credit to the original author(s) and the source, provide a link to the Creative Commons license, and indicate if changes were made.
In cases where reviewers are anonymous, credit should be given to 'Anonymous Referee' and the source.
The images or other third party material in this Peer Review File are included in the article's Creative Commons license, unless indicated otherwise in a credit line to the material. If material is not included in the article's Creative Commons license and your intended use is not permitted by statutory regulation or exceeds the permitted use, you will need to obtain permission directly from the copyright holder.
To view a copy of this license, visit https://creativecommons.org/licenses/by/4.0/

Response to Reviewer 1.

We thank the reviewer for the valuable comments, which have greatly improved the clarity and scientific value of the manuscript. Below, we provide a point-by-point response to each comment. The reviewer's comments are shown in black, and our responses are provided in purple.

The authors of this manuscript have investigated a new approach for classifying atmospheric moisture sources for the South American continent. Using a method to classify moisture sources based on a clustering of moisture backtracking analysis, he authors perform a comprehensive categorization scheme of moisture source regions. They then suggest management applications of their approach.

I think this work, if substantially revised, could be interesting. However, the current manuscript does not adequately explain why this classification is both worth digging into more deeply, and how it meaningfully is distinguished from other approaches. Notably this submission is to Nature Communications which requires a very high bar of both scientific novelty and clarity of presentation.

I recommend either comprehensive revision & resubmission of this manuscript, or rejection.

We thank the reviewer for these valuable suggestions. We agree that the clarity and coherence of the manuscript should be improved. Accordingly, we have revised our problem statement, clarified the presentation of our methodological novelty, and highlighted our main findings and their implications. Our study improves the understanding of long-term continental moisture drainage patterns and advances knowledge of aerial river characteristics. It introduces systematic methods for identifying turning points on moisture drainage curves, enabling the estimation of moisture sustained by critical upwind basins through long-term aerial river patterns. Unlike the universal thresholds employed in previous studies, our turning point method allows case-specific estimates which can be quantitatively integrated into broader water resource management assessments. By addressing the threshold challenge and advancing knowledge of long-term moisture drainage, our analysis provides a foundation for developing a framework to evaluate the potential of aerial rivers and support their integration into water management. We agree with the reviewer that this distinction should be clarified to better highlight the relevance of our results, and we

sincerely appreciate this insightful observation.

GENERAL COMMENTS

1. Distinguishing more clearly how the authors' work is new: Given this is a Nature Communications, there is a substantial expectation of novelty. The authors need to do a better job of crediting existing scholarship and highlighting where they have actually done something new.

We agree with the reviewer that it is essential to highlight the novelty of our work. In response, we have more clearly articulated the research gap and added relevant references (P2L23–P3L21). We have clarified the new methodology used to investigate turning points (P1L8–L9, P4L1–L7) and provided a more detailed description of our methods (P22L14–L32). Additionally, we have explicitly discussed new insights into aerial river drainage systems, further elaborated on critical upwind basins in the context of moisture transport patterns, and addressed the sharp decline in transport efficiency between G2 and G3 regions as suggested by the reviewer. (P13L6–L36). Furthermore, we have strengthened the section on aerial river management by incorporating key references and clarifying how our approach builds upon and extends ongoing discussions on moisture governance (P14L32–L36, P15L11–34). Finally, we have highlighted our main findings, including the novel method for identifying critical upwind basins, to address the research gap in the revised discussion section (P14 L10-L18, P20L17-L33).

2. Consistency in referencing: There are quite a few references that are not apparently correct. Given this, the authors need to very carefully review which papers are referenced, and for what.

We have checked the reference indicated by the reviewer later and specified the relevant parts we cited in the response. We have also improved specification in P3L15 and added relevant literature in P2L23-L33, P15L18, P15 L19-L34.

3. Generalizability of findings: The South American continent is well-studied for moisture recycling, but has specific characteristics that render generalizability challenging at best. The presence of tropical moisture colliding with the Andes mountains is but one of the challenges for generalizability of something like moisture curves.

We agree with the reviewer that moisture drainage types are shaped by the geographical

specificity of each continent. We focused on South America, where aerial river studies initially began, to better complement previous findings (P4L10-L12, P14L10–L18). We have also elaborated on how drainage patterns reflect regional climate systems (P13L24–L36), which helps to interpret our results and their broader implications. Moisture drainage curves capture the long-term influences of both regional climate and topography. Although these curves may differ across continents, this does not limit their applicability for investigating drainage types in other regions. This represents an exciting avenue for future research, which we have highlighted now in P20L29–L33 and P25L14–L19.

4. Utility of findings: The authors argue that past methods of delineation of moisture source boundaries are limited, incomplete, and/or inappropriate. However, the G1, G2, G3 and G4 definitions seem to be areas that receive progressively less moisture from the ocean, and progressively more from continental regions. This assertion is supported by the authors' own results, where they highlight "Aerial river management potential:" and then list the percent of total rainfall provided by the critical upwind basin. There is an intriguing difference between G2 and G3, apparently, but the authors do not dig into this enough.

We appreciate the reviewer's comment and understand that our previous description may have caused some misunderstanding. We have improved the manuscript's flow to clarify this point and revised the text to specify that our approach uses turning points to identify critical upwind basins, providing a mathematically, rather than subjectively, determined boundary for identifying moisture sources (revised in P2L31-L33, P3L7-L21, P3L23-P4L20, Fig. 4, Fig. 5, discussed in P14L10-P15-L34, and highlighted in P20L17-L22). Explicit discussion of critical upwind basins has also been added in P13L6–L14. Variations in moisture drainage curves allow us to identify four distinct types along the aerial river, each characterized by different turning point thresholds, critical upwind basin sizes, and drainage conditions (P13L15-L23). We also appreciate the reviewer's observation regarding the difference between G2 and G3, which we find insightful, and have added explicit discussion on this in P13L15–L36.

5. Management discussion is incomplete: The authors are suggesting that their work provides a relevant insight for moisture management, yet they fail to comprehensively engage in the substantial literature that now exists on this subject. If the authors want

to keep this discussion in their paper, they will need to much more substantially highlight how their work complements other approaches that have already been published (and the authors include many though not all those references), as well as highlight where their suggestions diverge from or advance beyond past approaches.

We thank the reviewer for the suggestion and have enhanced the discussion on moisture management (P14L32-P15L34). We acknowledge that the previous version did not sufficiently engage with the existing literature on the governance of atmospheric moisture. In the revised manuscript, we have strengthened both Introduction and Discussions section by incorporating key reference addressing moisture resource governance (P2L23–P3L21, P10L20–L23, P14L32–L36, P15L11–34). These additions clarify how our approach builds upon and extends ongoing discussions on moisture governance.

SPECIFIC COMMENTS

Page 2 Line 13. Do the authors mean topography? Altitude, at least on its own, is not what channels surface water. Topographic surface gradients, or something similar, would be a more appropriate phrase.

P2 L13 Revised as suggested (Now P2L26).

Page 2 Line 20. The reference of Keys et al. might be wrong here, since New Delhi is not apparently referenced in Ref #20. Please double check the referencing throughout.
P2 L20. We thank the reviewer for pointing this out. Figure 4 of Keys et al. (2012) traces the upwind basins of major cities, with the region we referenced labeled as "Pakistan" in the figure. We have revised our manuscript P3L15 to align with this reference and appreciate the reviewer for specifying it.

Page 3 Line 12. As highlighted above the Authors will need to explain how their specific study can lead to more generalized findings.
We agree with the reviewer that moisture drainage types are shaped by the geographic and climatic specificity of each continent. We have elaborated on how drainage patterns reflect regional climate systems (P13L24–L36), thereby improving the interpretation of our results and their broader implications. Moisture drainage curves capture the long-term influences of both regional climate and topography. Although these curves may

vary across continents, this does not limit their applicability for identifying drainage types elsewhere. We have now highlighted in P20L29–L33 and P25L14–L19 how our methodological framework can be extended to other geographic contexts in future research.

Page 3 Line 19. I suggest choosing a single phrase to refer to the source regions, since having two names ("atmospheric basins" and "upwind regions") is not contributing to clarity.

P3L19 We thank the reviewer for the suggestion and have standardized our terminology, using "atmospheric basin" consistently for clarity (P4L17–L18).

Page 4 Line 1. The authors refer to G1 types as receiving a high percentage of terrestrial moisture from a small upwind basin. Is this simply because it's proximate to the coast? Most of the moisture falling in the G1 areas are coming from the ocean anyway, so this is an odd classification.

Page 6 Line 21. The authors are defining a new term ("head-moistureshed") for the flow of moisture when it transitions from ocean to continental areas. I would suggest that this is unnecessary and that the authors should simply call this "G1 Ocean to Continental regions."

P4L1 P6 L21. We thank the reviewer for raising this question. The decrease in the size and dominance of critical upwind basins from G1 to G4 reflects that, as aerial rivers move inland, moisture delivery becomes less efficient and increasingly constrained by prevailing wind patterns. We have further elaborated on this point in P13L6–L23. While G2, G3, and G4 also encompass large coastal regions, G1 does not necessarily correspond to coastal areas, but rather to the prevalent entry point of moisture flow into the continent, which means the entry point of continental aerial rivers, as clarified in P10L29–L31. To better convey this concept, we have revised the term to "continental aerial river headwater regions" (P10L26). In addition, we updated Fig. 2a (P6) to display the mean wind advection field over the ten-year analysis period, helping readers better understand this concept.

Page 7 Line 7. In this paragraph, the authors use phrases like "shift left" and "shift upward", and it's not clear to what they are referring. Is this an XY plot? Is this on a map? I encourage the authors to be much more precise in their language here.

P7L7 We agree with the reviewer and have revised the wording for clearer and more precise delivery (P11L15 and L20). Additionally, we have added Supplementary Figs. S4a and S4b(referred to in P11L16, L22) to better illustrate the evolution of the curves in G2. Supplementary Fig. S4a shows the evolution of the drainage curve, including the turning points shifting to lower x-values and then moving upward toward higher y-values. We further highlight this evolution by distinguishing two example types (Chubut vs. Valle del Cauca) in Supplementary Fig. S4c (referred to in P11L19, L24L25).

Page 8 Line 1. At this point, I am confused what critical upwind basin means in practical terms. The authors have defined the concept mathematically. I think the authors have something here, but it is not clear in the text. The fact that the length scale of moisture transport is similar in G2 and G3 seems very important, since the authors are claiming there is a substantial difference between G2 and G3. I encourage the authors to dig much more deeply here.
P8 L1 We thank the reviewer for this insightful suggestion, which has greatly improved the clarity and relevance of our results. In response, we have added the definition of critical upwind basins (P4L19–L20) and discussed them in detail in P13L6–L23. We also appreciate the reviewer's observation regarding the differences between G2 and G3, which we found valuable, and have explicitly addressed this in P13L20–L23 and P13L31–L35.

Page 8 Line 20. There has been quite a lot written already about what the authors are calling "moisture management". Yet there does not seem to be much explicit engagement with that literature. If the authors want to retain this section, the authors need to provide much more explicit engagement with the existing literature that discusses governance challenges. Moreover, an important author on this subject that has not been cited is Sofie te Wierik:

te Wierik et al. (2021) Reviewing the Impact of Land Use and Land-Use Change on Moisture Recycling and Precipitation Patterns. https://doi.org/10.1029/2020WR029234

te Wierik et al. (2019) The need for green and atmospheric water governance. https://doi.org/10.1002/wat2.1406

We thank the reviewer for highlighting this point. We acknowledge that the previous version did not sufficiently engage with the existing literature on the governance of atmospheric moisture. In the revised manuscript, we have strengthened this section by incorporating key references, including te Wierik et al. (2019, 2021), along with other relevant works addressing the governance of moisture resources. These additions (P2L23–P3L21, P10L20–L23, P15L11–L34) help clarify how our approach builds upon and extends ongoing discussions on moisture governance.

Keys et al. (2017) Approaching moisture recycling governance.
Elison et al. (2017) Trees, forests and water: Cool insights for a hot world.
Keys et al. (2019) Invisible water security: Moisture recycling and water resilience.
Whaley (2022) Water governance research in a messy world: A review.
Williams and Brown (2014) Adaptive management: from more talk to real action.
Allen et al., (2011) Adaptive management for a turbulent future.

Page 13 Line 18. The authors claim that their use of annual flows of moisture is sufficient to build a picture of aerial rivers. But what about interannual variability? The authors need to clarify how a relatively short record (~10 years) is sufficient to eliminate multi-year modes of variability from their analysis. Or, acknowledge the presence of these modes, and discuss the implications of this variability in terms of their findings. They do so with regard to mega-droughts. The same ought to be done for other modes of large scale climate variability.

P13L18 We thank the reviewer for this suggestion, which helps further strengthen the robustness of our findings. Indeed, continental-scale evaporation products have only become available in recent decades, limiting the full representation of aerial rivers within their climatological context. We have now acknowledged this limitation and added a discussion of the potential impacts of the selected time period on our results to enhance their contextualization (P24L22-L33). We examined the interannual variability of our results, showing modest interannual variability throughout the study period (P24L23-L24, Supplementary D, Fig. S7). We have also incorporated a discussion of our findings, considering the impacts of major regional climate variabilities, including ENSO, PDO, and AMO (P24L22-L33, Supplementary D).

Page 13 Line 24. I encourage the authors to share the mathematical basis of their calculations. The key equations would be helpful here. At present, the methodological

approach for calculating the turning points is insufficient.

P13L24 We agree with the reviewer that the calculation of turning points should be clarified. Accordingly, we have added a paragraph (P22L14–L32) detailing the procedures for deriving data curves and identifying turning points, including the corresponding equations (P22L26–L32) used to detect local peaks in the process.

Page 13 Line 35. This is an example of where the authors go into great detail about the k-means operation they perform (a very standard statistical method). A similar level of detail would be useful for the turning points mentioned above.

P13L35 We thank the reviewer for pointing this out, and we have revised the section on turning points as suggested (P22L14–L32).

Page 14 Line 14. Did the authors construct their own point tracking algorithm "based on the Lagrangian specification of motion." If so, that's very interesting. However, it would be useful for the authors to explicit state that.

P14L14 Yes, and we thank the reviewer for acknowledging our work. We have now explicitly stated this in P23L31 and made the corresponding code available in the Code Availability section to enable replication of our results.

Page 14 Line 21. How were residence times calculated? The trajectory of the parcel is straightforward based on the description, however the residence time is often prescribed in Lagrangian calculations. More explanation is needed.

P14L21 We thank the reviewer for raising this question. In this context, residence time refers to the duration that a water vapor molecule remains in the atmosphere before precipitating. Lagrangian moisture tracking models do not typically prescribe a fixed residence time. Rather, they simulate the trajectories of air parcels (or moisture particles) using wind fields and other atmospheric data, so that residence time emerges from the modelled advection and removal processes (in our algorithm, “removal” corresponds to the starting point of the backtracking). In our implementation, the tracking used long-term averaged ERA-Interim 843 hPa wind data (2006–2015) as the velocity field for aerial rivers. We applied a backtracking approach by initializing particles at the grid cells corresponding to each moisture drainage type and tracing their trajectories backward to the ocean under a reversed velocity field. Particle positions were updated at 3-hour intervals by solving the Lagrangian equations of motion with time-stepping scheme, and residence time was then calculated as the interval from ocean entry points

to the target grid. We have clarified this point in the text (P23L38–P24L2).

Response to Reviewer 2.

We thank the reviewer for the insightful comments, which have greatly strengthened the clarity and scientific quality of the manuscript. A point-by-point response to each comment is provided below, with reviewer comments in black and our responses in purple.

General comments:

I found the paper interesting and innovative but also confusing due to the terms used for water in the atmosphere, such as aerial rivers, atmospheric rivers, flying rivers, and low-level jets (as mentioned in some of the listed references). Are they the same? If not, what is the difference between them? In any case, while comparing surface rivers and aerial rivers in general terms may make some hydrological sense, using surface rivers in planning and management may be more sensible than using aerial rivers, which are derived from reanalysis (ERA-interim) and whose results may change if other reanalysis products are used, making these calculations uncertain. In sum, there are limits on what the results can be used for, but I do not believe they could be used for the management of water resources in geographic regions or even to discuss tipping points.

Therefore, I believe that major reviews are necessary.

We thank the reviewer for these valuable suggestions. In the age of growing population and environmental changes, it is reasonable to consider alternative water resources. The reviewer is correct that intangible atmospheric moisture relies on moisture tracking algorithm and Earth observation data to describe, for now. There are data and modeling uncertainties involved, although these have been shown to be minor, as indicated in Zemp et al. (2014, Table 2, p. 13344). We thank the reviewer for the valuable suggestion and have accordingly enhanced our discussion of these uncertainties in the revised manuscript (P25L1-L8 and Supplementary E).

Low-level jets (LLJs), atmospheric rivers, and aerial rivers each represent distinct modes of atmospheric circulation and moisture transport, differing in both scale and persistence. LLJs form when pressure gradients, the Coriolis force, and terrain effects combine to accelerate winds in a concentrated corridor, often at night when surface

friction decreases. LLJs have the most common temporal scale of diurnal or seasonal cycle, such as the South American low-level jet (SALLJ) is prominent during austral summer. Atmospheric rivers, by contrast, are transient corridors of concentrated water vapor that affect limited areas of the planet and usually persist for less than a day. Aerial rivers (also called flying rivers) differ from both in that they are not short-lived events but rather long-term statistical pathways of moisture flow, persisting over years to decades and integrating dynamic variations in wind.

Although they share similar names, aerial rivers are preferential pathways of moisture flow in the atmosphere and differ from atmospheric rivers both spatially and temporally. Atmospheric rivers are short-lived, covering specific areas on Earth for typically less than a day. In contrast, aerial rivers represent long-term preferential pathways for moisture transport, spanning several years to decades containing dynamic changes of wind. We have clarified this in the Introduction (P1L29–P2L4) and further discussed our results in response to the reviewer's comments (P13L24–L36).

Our study advances the understanding of aerial river characteristics and provides systematic methods for identifying turning points on moisture drainage curves as a tool for assessment. This quantitative framework facilitates the identification of key areas for land management to conserve or regulate moisture inputs to aerial rivers. We acknowledge that the previous version lacked a clear explanation of how these findings could inform water management (now clarified in P14L32–P15L34, Fig. 4, Fig. 5, and Supplementary C) and did not adequately discuss the uncertainties inherent in our estimates (revised in P25L1-L8 and Supplementary E). We thank the reviewer for these critical comments. Additionally, while our results have implications for ecological tipping points, the earlier presentation may have caused misunderstanding. We have revised P19L14–L18 and P20L11–L15 to clarify and strengthen these points.

Zemp et al. (2014). On the importance of cascading moisture recycling in South America. Atmospheric Chemistry and Physics, 14(23), 13337–13359. https://doi.org/10.5194/acp-14-13337-2014

Specific comments:

Page 1:

Line 14: it is a bit unnatural to define boundaries to atmospheric flow; nature does not

work this way.

P1L14 We understand the reviewer's concern. Here, the term "hydrological boundaries" refers to distinctions among moisture drainage types rather than sharp divisions, as in Köppen's climatic classification. We have therefore revised the term to "hydrological category." to enhance clarity (P1L1, P1L17)

Line 30: Please discuss the differences between atmospheric rivers and aerial rivers, as well as low-level jets.

P1L30 We have added a clarification to differentiate these often-confused terms as suggested (P1L29-P2L4).

Line 32:... and also from moisture transport from the winds, that in the Amazon are the trade winds from the tropical Atlantic. Atmospheric rivers are not formed in the Amazon region; they originate from outside the Amazon.

P1L32 We have added the description (now in P2L10).

Page 2:

Line 8: where are the aerial rivers born? In continental regions, are they fed by evapotranspiration only?

P2L8 Aerial rivers represent preferential pathways of moisture transport traverse any given region. Unlike low-level jets (LLJs) or atmospheric rivers, they do not have a clearly defined starting or ending point, but are characterized by their flow direction. The reviewer is correct that, when flowing inland, their moisture load is recharged by evapotranspiration.

Page 3:

Line 6: why Santa Cruz de la Sierra?

P3L6 As an example, Weng et al. (2019) examine Santa Cruz de la Sierra, a water-stressed city, to illustrate how aerial river management could provide an alternative water resource.

Page 4:

Line 7: which regions show the contribution of moisture from the Atlantic? The Atlantic is also a source of moisture.

Line 25: again, where are the sources of moisture and rainfall? Are they all on the

continent? What about the Atlantic and Pacific Oceans?

P4L7 and P4 L25 For any given region, the moisture-tracking tool can distinguish the relative contributions of terrestrial and oceanic rainfall. In response to the reviewer's question, our tracking results indicate that every grid cell across the South American continent receives rainfall originating from both continental and oceanic sources, primarily the Atlantic and the Pacific. As shown in Table 1, the G1 region derives approximately 88% of its rainfall from oceanic sources (continental 12%), whereas the G4 region receives nearly equal contributions from terrestrial and oceanic sources. Aerial river management is therefore more pertinent in regions such as G4, where dependence on terrestrial moisture sources is stronger. In this study, we focus on continental aerial rivers—that is, the terrestrial component of rainfall—because of their relevance for water management through upwind land interventions (Elison et al., 2017; Keys et al., 2018; Weng et al., 2019). Accordingly, our moisture drainage curves are derived from terrestrially sourced rainfall. Although this was described in the Methods (P22L10–L11), we recognize that the focus on terrestrial aerial rivers should be stated more explicitly in the Introduction, and we have added this clarification at P4L12–L15. In the Discussions, in addition to estimating the critical upwind basin regime for terrestrially sourced rainfall, we also provide estimates of total rainfall—including both terrestrial and oceanic contributions—for reference, and have explicitly clarified this in P11L5–L8, P11L31–L32, P12L19–L20, and P13L4–L5.

Keys et al., 2018. Megacity precipitationsheds reveal tele-connected water security challenges.

Weng et al., 2019. Aerial river management by smart cross-border reforestation.

Elison et al., 2017. Trees, forests and water: Cool insights for a hot world.

Page 6:

Line 10: each region shows a different type of vegetation, how the evapotranspiration of different vegetation types is included>, for instance, over the Amazon forest or the Cerrado region in west central Brazil

Table 1, second column: again, what is the role of vegetation type in each region? How is the continental recycling ratio calculated?

P6L10 and Table 1 In this study our evapotranspiration input data is from the GLEAM (Global Land Evaporation Amsterdam Model) product. In GLEAM, vegetation type is represented indirectly through satellite-derived biophysical variables rather than fixed land-use classes. Key inputs include leaf area index (LAI) from MODIS, vegetation optical depth (VOD) from passive microwave missions such as SMOS, SMAP, AMSR-E, and AMSR2, and the fraction of vegetation cover, which together control the partitioning of evapotranspiration into transpiration, interception loss, and soil evaporation. Dense vegetation such as forests, with high LAI and VOD, exhibit greater transpiration and canopy interception, while sparsely vegetated or degraded areas contribute more strongly to soil evaporation. Seasonal vegetation dynamics, such as crop growth cycles or forest phenology, are captured through time-varying LAI and VOD, while long-term land-use changes such as deforestation or afforestation are implicitly reflected as these indices adjust over time. Over South America, GLEAM additionally assimilates soil moisture from the same passive microwave missions, which constrains how vegetation interacts with available water, and it is forced by reanalysis meteorological data to represent climate–vegetation interactions. In this way, GLEAM accounts for vegetation type and dynamics through continuous satellite observations, ensuring that differences in vegetation structure and cover are embedded in the model's evapotranspiration estimates.

As for continental recycling: In this study, the continental recycling ratio is obtained using the WAM-2layers (Water Accounting Model 2-layers) framework, which explicitly tracks atmospheric moisture from its source to precipitation. For each grid cell, the model tracks the fraction of precipitation that originates from continental evapotranspiration versus oceanic sources. The continental recycling ratio for a grid cell is then defined as the fraction of its total precipitation that comes from continental evapotranspiration. We thank the reviewer for this question and have added this explanation in the Methods section (P23L27–L30).

Line 19: I do not see this clearly; contrary to surface rivers, where one can delineate the basin and measure the flow, aerial rivers are more abstract, and calculations are more based on assumptions that may change if one changes the atmospheric data sets used for the calculations. How can one use this data for environmental policies (forest restoration, etc.)? The aerial river calculations are subject to uncertainties, which may

be much higher than measures in surface rivers.

Page 7:

Line 30: River management, surface river management? How can we manage something we do not see when measurements show uncertainties?

P6L19 P7L30 In the context of future environmental change and growing water stress, considering multiple management options—including the conservation and regulation of atmospheric moisture—can enhance resilience and complement limited surface water resources. Although aerial rivers are less tangible than surface rivers, they resemble other invisible climate variables, such as carbon dioxide or ozone, which require scientific tools to understand their dynamics, societal relevance, and potential for regulation. While current tracking methods generally show good agreement, the reviewer is correct that aerial river estimates carry uncertainties. As science-based decision-making inevitably involves such uncertainties, acknowledging and addressing them is central to adaptive management (Allen et al., 2011; Williams and Brown, 2014). We thank the reviewer for this reminder and have enhanced our discussion of uncertainties (P25L1-L8 and Supplementary E). We acknowledge that the previous version did not clearly articulate how our results could contribute to water management. Our study advances the understanding of aerial river characteristics and provides systematic methods for identifying turning points on moisture drainage curves, enabling estimation of moisture sustained by critical upwind basins through long-term aerial river patterns. These estimates can be quantitatively integrated into broader water resource management assessments. We have therefore clarified this in P14L32-L36 to specify that these estimates can inform water planning but should be incorporated into cost–benefit analysis, active monitoring, and adaptive management to address inherent uncertainties (revised in P15L19-L34). Furthermore, in addition to the conceptual framework, we have added practical examples that better illustrate how critical upwind basins can be identified and used to guide decision-making (Fig. 4, Supplementary C, and Fig. 5). Future studies can build on these insights, benefiting from advances in Earth observation data and modeling techniques to further reduce uncertainties.

Allen et al., 2011. Adaptive management for a turbulent future.

Williams and Brown, 2014. Adaptive management: from more talk to real action.

Line 30: does this suggest that 11-12% of moisture comes from the ocean and the rest is from continental origin? Does this mean that 11-12% of the moisture comes from the sea, and the rest is from continental origin?

P6 L30 *With continental -sourced rainfall averaging 12% of total precipitation,* this means that about 12% of the precipitation originates from the continent. We have revised P11L5–L8 to convey this more clearly.

Page 7:

Line 2: this number., as well as from the other G regions, needs to be better explained

P7 L2 We thank the reviewer for the reminder. We have revised in P11L5–L8, P11L31–L32, P12L19–L20, and P13L4–L5 as suggested.

Page 8:

Line 29: The mathematical analysis is good; it may show uncertainties but seems to be good; now, the aerial rivers are not surface rivers where you can see the water and the basin and measure the flow. You can use the surface river data for water resources management, but doing this management with something more abstract, such as aerial rivers, I am not sure if this is the way to go.

Page 9:

Line 13: You can use the surface river data for water resources management, but doing this management with something more abstract, such as aerial rivers, I am not sure if this is the way to go.

P8 L29 P9 L13 We thank the reviewer for acknowledging our mathematical analysis. Our results provide estimates of the moisture sustained by critical upwind basins through the long-term patterns of aerial rivers, which can be quantitatively integrated into broader assessments of water resource management. This represents one potential component within an integrated water resources management framework. In some regions, managing surface water may be more feasible or urgent, whereas in others—particularly those already under water stress—the atmospheric moisture component may offer a complementary or alternative option. We acknowledge that these estimates carry uncertainties and have enhanced our discussion of uncertainties in P25L1-L8.

While similar to other components of water management, such as flood peak prediction or soil moisture estimation, which also involve inherent uncertainties, our estimates, when considered in water planning, should likewise undergo cost–benefit evaluation, stakeholder consultation, and active monitoring, with adaptive management explicitly designed to address uncertainty in decision-making (Allen et al., 2011; Williams and Brown, 2014). We have therefore included this clarification in P15L19-L34 and thank the reviewer for the reminder.

Allen et al., 2011. Adaptive management for a turbulent future. Journal of Environmental Management 92: 1339–1345.

Williams and Brown, 2014. Adaptive management: from more talk to real action. Environ Manage. Feb;53(2):465-79. doi: 10.1007/s00267-013-0205-7.

Line 15: I am not sure if we have reached that level of refinement in managing the water rivers; this is an abstraction with uncertainties.

P9L15 We agree with the reviewer that there was a jump between our results and their application to management. Therefore, we have revised to specify how the results should be incorporated into standard water management processes (P14L32-P15L18 and P15L19-L34), which should be accompanied by institutional arrangements, impact assessments, and stakeholder engagement for adaptive management. This normally includes cost–benefit analyses and active monitoring in the face of uncertainties (P15L27-L34 and Fig. 4).

Line 26: I believe that the authors are expecting too much from these results.

P9L26 In this instance, we refer to methods in cited literature. We have revised the sentence accordingly to prevent potential misunderstanding (P15L23-L25).

Page 10:

Line 8: how much manageable is possible?

P10L8 If we understand the reviewer's question correctly, this means the portion of freshwater resources that can be sustainably used or controlled for human purposes without compromising ecosystem health. To response to the reviewer's question, globally, total renewable freshwater is approximately 43,000 km^3/year, of which current

human withdrawals are ~4,000 km³/year (~10%). Considering environmental flow requirements and accessibility constraints, roughly 13,000–20,000 km³/year can be considered sustainably manageable for human use (FAO, 2025).

We noted from the reviewer's question that 'manageable' here in the sentences might also imply 'under control,', which can be misleading. We have rephrased and revised the paragraph for clarity and flow (P18L4-L6). We thank the reviewer for raising this point.

Food and Agriculture Organization of the United Nations (FAO). (2025). Annual freshwater withdrawals, total (billion cubic meters). Retrieved September 18, 2025, from https://data.worldbank.org/indicator/ER.H2O.FWTL.K3

Line 14: I am sorry, but I do not see that
Line 16: this is a good example of connectivity,
P10L14 P10L16 We would like to thank the reviewer for acknowledging our example for the connectivity between aerial rivers and surface rivers. In P10L14 of the previous version we actually mean that integrated water resources management should account for this connectivity. We have revised now in P18L9-L16 to clarify and strengthen our argument.

Page 11:
Line 4: what does this mean?

P11L4 Surface river regimes shape power relations by determining who controls access to water across time and space. Upstream actors often gain leverage through dams or diversions that control flow timing and water allocation, leaving downstream users dependent on managed releases. Likewise, upwind regions could influence moisture delivered by the aerial rivers to downwind regions. In the Amazon, the downstream portion of the basin lies upwind of aerial rivers, a configuration that could reshape existing power relations shaped by surface rivers. We have added this in the text at P18L30-L34 to better clarify what we mean.

Line 13: This kind of figure (Figure 5) was previously shown in studies considering the low-level jet east of the Andes in South America. In the figure, it appears that flying

rivets are present, and the paper discusses aerial rivers and atmospheric rivers. Are all of those the same?, the components of the South American monsoon system?

P11L13 (Figure 5) Aerial rivers are also referred to as “flying rivers” (P1L30). They are different from the SAMS, LLJs, and atmospheric rivers in both temporal and spatial scales. The South American Monsoon System (SAMS) is the dominant seasonal circulation controlling wet and dry seasons across tropical and subtropical South America, driven by land–sea contrasts and convection. Low-level jets (LLJs), in contrast, are narrow, fast-moving air currents in the lower troposphere that vary mainly on diurnal to seasonal timescales, while atmospheric rivers are transient corridors of concentrated water vapor, typically lasting less than a day and spanning ~400–600 km. Aerial rivers, by comparison, operate at broader spatial and temporal scales. Aerial rivers describe long-term statistical pathways of moisture transport that integrate the variability of wind systems, rather than episodic or regional events like SAMS, LLJs, or atmospheric rivers. In doing so, aerial rivers embody the cumulative influence of short-term processes—including land–sea breezes, SAMS, LLJs, atmospheric rivers, and other atmospheric events—capturing their net effect on moisture delivery at climatological timescales. As suggested by the reviewer, we have added a clarification in the Introduction (P1L29–P2L4) and expanded the discussion of aerial river drainage to address the influence of regional periodic mechanisms, including ARs, LLJs, and terrain-induced flows (P13L24-L36).

Figure 5 (now Fig. 6) illustrates the spatial relationship between aerial rivers and surface rivers across two basins. The reviewer is correct that readers familiar with studies on SAMS or SALLJ may observe similar patterns, as these atmospheric systems are contributors to regional moisture transport and are encompassed within the aerial rivers identified in both the Amazon and La Plata basins (discussion added in revision P13L24-L36). At long-term scales, however, aerial rivers reflect stable upwind–downwind connections, allowing for analogy and comparison with surface rivers, which operate at similar timescale. Fig. 6 highlights contrasting dynamics: in the Amazon, the reversal between upwind–downwind and upstream–downstream positions enhance the hydrological integrity of interconnected aerial and surface rivers, whereas this reversal does not occur in the La Plata basin. Although aerial rivers are also referred to as ‘flying rivers’ (P1L30), for consistency we have revised the label in Fig. 6 from

‘Flying rivers’ to ‘Aerial rivers’ (P19, Fig. 6). We thank the reviewer for pointing this out.

Line 20: I am unsure if this term can be used in this paper, as the objective is not to define whether we have reached or even if we have defined a tipping point. I do not think that, based on the evidence presented in this paper, we can discuss reaching or surpassing a tipping point (ecological, climate-social).

P11L20 The reviewer is correct that, based on our results, we cannot determine whether the ecological tipping point has been reached or surpassed. Our analysis instead suggests that tipping points may not be uniform: in addition to the well-recognized roles of size and scale, location also plays a critical role. Regarding aerial river influence, deforestation in critical upwind basins produces varying impacts on downwind regions depending on the drainage type. We have revised this long sentence (P19L14–L15) to more clearly convey our intended discussion (P19L14-P20L15).

Response to Reviewer 3

We thank the reviewer for the valuable comments, which have greatly enhanced the clarity and scientific quality of the manuscript. Below, we provide a point-by-point response to each comment. Reviewer's comments are shown in black, and our responses are provided in purple.

Summary:

This paper classified the South American region into four categories based on the moisture drainage curve. The moisture drainage curves were generated based on the moisture tracking results, focusing on a climatological scale with the concept of "aerial rivers". The study further examined the derivative of the curve angle to quantify the critical upwind basin size for each region. Such a characterization analysis was also conducted in the aspect of aerial rivers (i.e., physical characteristics of aerial rivers). Finally, the study discussed the potential of applying the proposed framework for water resource management in the target region.

Overall assessment:

I believe the concept of defining hydrological boundaries based on the critical upwind source region, rather than the conventional watershed boundary or municipal boundary, is interesting.

The regional difference in classification is worthwhile investigating to better understand the water source of such a region based on the provision of atmospheric moisture. Such understanding would be useful for future water resource management in the region.

We thank the reviewer for acknowledging the relevance of critical upwind basins and drainage types, as defined by continental moisture provision, for guiding future water resource management in the region.

However, I have major concerns about the difficulty of interpreting the results, the uncertainty with the employed methods, the novelty of the study, and the insufficiency of the proposed framework.

I have some moderate comments, which I believe are all essential to enhance the value of this paper.

Please find my comments below:

We thank the reviewer for the constructive comments, which have improved both the

clarity and scientific robustness of our study. We have included dominant wind pattern to Fig. 2 and integrated Fig. S5 into Fig. 4 to improve the interpretability of our results. Additionally, we have included examples for each G1–G4 type in Fig 5. and Supplementary C.

We agree with the reviewer that the discussion of uncertainties and their relevance to our results should be strengthened. We have now included a discussion of modeling uncertainties associated with both our tracking and clustering methods (P25L1-L8, Supplementary E, Fig. S8 and Fig. S9).

We also agree that the introduction of our problem statement and the presentation of the novelty in our methodology could be more deliberate and better connected to highlight our main findings and their implications (revised in P1L8L9, P2L23–P2L33, P3L7–L21, P3L23–P4L15). Furthermore, we recognize that the link between our results and their management implications could be more clearly articulated (P14L32–L36). In addition, we agree that the estimates obtained using our method should be further assessed through cost–benefit analysis and stakeholder engagement to prioritize water objectives, alongside the necessary institutional arrangements for integration into water management (revised in P15L19–L34). Revisions to other sections and our responses are detailed in the point-by-point responses.

Major comments

-Difficult interpretability of the results:

It was quite hard to physically/geographically interpret the findings like the ones shown in Figure 2. In that sense, I found Figure S5 extremely useful to understand the actual extent of the inland upwind basin. My suggestion about showing the dominant wind pattern is also relevant.

I suggest the authors incorporate the figures showing the actual extents of the critical upwind basins as well as the dominant wind pattern (you can test and see), ideally for G1-G4 (maybe one or two for each region?).

We thank the reviewer for the constructive comments. As suggested, we have included dominant wind pattern to Fig. 2, integrated Fig. S5 into Fig. 4, and added Fig. 5 to improve the interpretability of our results. Additionally, we have included examples for each G1–G4 type in Fig 5 and Supplementary C.

-Uncertainty in the employed method:

I see the authors' statement about the significant sensitivity of the fixed threshold to determining the classification. I believe that such sensitivity also applies to the clustering method (also the moisture tracking method). Quantifying the uncertainty associated with the method would be important. This is also because the authors suggested the framework in Figure 4 in a general way in which the readers have some freedom to select the methodology in clustering and moisture tracking. Performing several methodologies may not be realistic, but could the authors at least somehow quantify the uncertainty rooted in the methodology? Please consider.

We agree with the reviewer that the discussion of uncertainties and their relevance to our results should be strengthened. Previously, we only addressed data uncertainty from moisture tracking. Following the reviewer's suggestion, we have now included discussion of modeling uncertainties associated with both our tracking and clustering methods. (P25L1-L8, Supplementary E, Fig. S8 and Fig. S9).

-Novelty of the study:

I do not clearly see the novelty of this study. It reads as just applying the existing methodology to the continental scale. I suggest re-structuring the introduction and discussion based on the following points:

- The authors need to (more) clarify the novelty in the methodology if there is any.
- The authors need to better structure the problem statement, which ideally is connected to the novelty statement.

We thank the reviewer for the reminder. Methodologically, while we were inspired by Weng et al. (2019) to investigate drainage curves and turning points, their study did not specify how turning points should be determined. Our approach was to identify turning points through changes in the curve angle, which indicate where the per-area contribution decreases rapidly, and this represents one novelty in our method. This method also revealed what has not been recognized before: that turning points exist more generally across the continent, allowing us to capture continental-scale variability. These variations in the drainage curves reflect diverse long-term continental moisture drainage conditions that have not yet been systematically explored. From our cluster analysis, we identified drainage types which are geographic results of long-term of continental moisture flows. They represent distinct moisture dependencies and aerial

river drainage conditions across the continent, which shape regional hydrological processes and determine the reception of freshwater resources. We agree that the introduction of our problem statement and the presentation of the novelty in our methodology could be more deliberate and better connected to highlight our main findings and their implications (revised in P1L8L9, P2L23–P2L33, P3L7–L21, P3L23–P4L15). We have explicitly discussed new insights into aerial river drainage systems and critical upwind basins (P13L6-L36). We also recognize that the link between our results and their management implications could be better articulated (revised in P14L32–L36, and P15L19–L34). We have incorporated the actual case of Sorriso into Fig. 4 to illustrate the identification of the critical upwind basin using the moisture drainage curve and turning points (Fig. 4a, 4b), and we have added representative examples for each drainage type in Fig. 5. We thank the reviewer for this valuable suggestion.

Weng et al. (2019). Aerial river management by smart cross-border reforestation. Land Use Policy.

-P9 Lines 3-26 and Figure 4:

I have major concerns about the insufficiency of the suggested framework regarding the points below.

- The practice of the author's proposal (e.g., reforestation) for a region (let's say Sorriso in G3) should affect the downstream. Thus, it seems to need an optimization for multiple regions. This may apply not only to the downstream close by, but also to much larger regions. I understand that Figure 4 is just a suggested framework. However, if the authors insist on suggesting an actual framework in this high-impact journal, the authors should be mindful of the potential impact of the suggested plan. Does it not reduce the downstream water availability? Unless the authors show a scientifically/socially reasonable argument, I do not believe it is ready to suggest as a framework. In fact, this may work as a maladaptation. Please explain/revise.
- As being one Figure to be impactful enough, I believe the figure needs to be not only conceptual but also show the actual example. Why don't the authors consider integrating Figure 4 and Figure S5? Please consider.

P9 L3-26 The reviewer is correct that reforestation can influence downstream water

availability. While flying rivers provide benefits, they may also entail costs elsewhere, making it important to assess and prioritize the trade-offs between costs and benefits. For example, in the study we discussed previously, Weng et al. (2019) demonstrated in the Santa Cruz case that reforesting the upwind basin, rather than the upstream basin of a water scarce city, can secure aerial river benefits while minimizing the impact of surface water losses, given that the downstream region (Beni and Pando) of its upwind basin is relatively water-abundant. Our proposed framework emphasizes planning and assessing potential water resources, but we agree that these estimates should subsequently be subject to cost–benefit analysis and stakeholder engagement across affected regions to prioritize water objectives, along with institutional arrangements for integration into water management. We have incorporated this aspect into Fig. 4, and updated the figure by integrating Fig S5 (now Fig. 4a and 4b). We have clarified this point further in the main text (P14L32–L36, P15L19–L34).

Weng et al. (2019). Aerial river management by smart cross-border reforestation. Land Use Policy.

Moderate

p1 Lines 29-30:

The sentence does not sound scientifically appropriate. I believe "aerial rivers" themselves represent climatology, not "like Earth's climate". The authors may consider phrasing "Aerial rivers are often described as climatology expressed through the persistent flow of atmospheric moisture over time." or anything similar.

P1 L29-L30 We thank the reviewer for pointing out this misleading sentence and have revised it as suggested (P2L2–L4).

P2 Line 1:

"[…] decrease in annual rainfall and a 19-50% decrease in annual runoff […]"

Did the authors mean a decrease in the downstream? Please specify.

P2 L1 The annual runoff decrease occurs within the Ucayali region itself. This reduction results from deforestation in upwind areas, which are located downstream of Ucayali in terms of surface river flow. Such deforestation reduces the moisture loading of aerial rivers, leading to decreased rainfall in Ucayali's upstream catchments (Weng et al., 2018). This point has now been clarified in P2L17–L20. We thank the reviewer for the suggestion.

Weng et al. (2018). Aerial and surface rivers: Downwind impacts on water availability from land use changes in Amazonia. Hydrol. Earth Syst. Sci.

P2 Lines 1-3:
The sentence about Peruvian Ucayali and the one about the more general can be combined.
P2 L1-L3 Revised as suggested to enhance the flow (P2L12-L20).

P2 Lines 5-6:
"are most likely inadequate"
"So, what should a water management system integrating aerial rivers be based on?"
The issue the authors raise here is vague. The authors need to be more specific about the issue in the gap between aerial river coverage and watershed/political boundaries. What do you mean by inadequate? In what sense is it inadequate? Why is it an issue not to consider aerial rivers in the current water management system?
Better clarification of the research gap and issue here is critical for highlighting the value of this paper.
P3 Line 2:
Here, the research question seems to be replaced with "the need to explore an objective standard for identifying upwind regions", which is not necessarily straightforwardly consistent with the water management question (see the comment above). The authors need to clarify what the research question is and better structure the introduction.

P2 L56 P3L2 We agree with the reviewer that the introduction should be more elaborated to clearly frame the main question addressed by our results. Previously, the text could have been misleading by suggesting a focus on inadequacies in water management, which was not our primary objective. The Introduction has been revised to better frame the research gap, highlighting how the challenge of identifying relevant upwind regions has hindered the integration of aerial rivers into water management, and emphasizing the need for a systematic approach to address this issue (P2L23–P3L21). This directly relates to our approach using turning points of moisture drainage curves (P3L23–P4L15). This focus also highlights how our findings on differences in drainage patterns reveal the limitations of conventional fixed-threshold methods used in previous studies, while advancing understanding of aerial river drainage. To better

explain the implications of our findings regarding critical upwind basins and moisture drainage types, we have revised the Discussions to more clearly connect to the problem statement and its broader implications (P13L6–P14L18, P14L32–P15L34, and P20L17–L33). This suggestion has substantially strengthened the flow and coherence of our study, and we thank the reviewer for pointing it out.

Figure 2

It is interesting to examine the large variability in Chile and Argentina, which makes sense based on their geographical shape.

For instance, the central to southern Chile and Argentina where heavy precipitation is dominated by ARs (Viale et al., 2018; Hiraga and Meza, 2025), is G1. Can we say a major portion of impactful "aerial rivers" are "ARs" in this region? Meanwhile, the northern Chile and southern Peru where convective systems occur (Huang et al., 2024) are classified as G3/G4.

I believe this shows the climatology rather than the extremes, but the regional classification seems to be somewhat related to the dominant storm types. I suggest the authors discuss the results in the context of such storm types.

References:

- Viale, M., Valenzuela, R., Garreaud, R. D., & Ralph, F. M. (2018). Impacts of atmospheric rivers on precipitation in southern South America. Journal of Hydrometeorology, 19(10), 1671-1687.
- Hiraga, Y., & Meza, J. (2025). Extreme precipitation modeling and Probable Maximum Precipitation (PMP) estimation in Chile. Journal of Hydrology: Regional Studies, 58, 102274.
- Huang, Y., Xue, M., Hu, X. M., Martin, E., Novoa, H. M., McPherson, R. A., ... & Flores Luna, A. J. (2024). Characteristics of precipitation and mesoscale convective systems over the Peruvian Central Andes in multi 5-year convection-permitting simulations. Journal of Geophysical Research: Atmospheres, 129(17), e2023JD040394.

Figure 2 We appreciate the reviewer's valuable comments. Indeed, moisture transport across regions is governed by different atmospheric processes. G1 is generally dominated by oceanic moisture sources. In southern Chile, a major portion of climatological moisture transport is associated with atmospheric rivers, as documented by Viale et al. Accordingly, the corresponding aerial rivers necessarily include this dominant component. From our perspective, this region is classified as G1 because the

dominance of oceanic moisture produces a nearly vertical feature in the continental moisture drainage curve.

Although mesoscale convective systems (MCS) are key components of aerial rivers, we consider them unlikely to influence the classification. First, their influence extends across all G1–G4 regions. For instance, Amazonian MCS contribute significantly to moisture discharge in G1 and G2 regions (Rehbein et al., 2017). Second, while storm types may govern the moisture discharge (precipitation) of aerial rivers, the underlying mechanisms that drive these storms are more likely the primary factors determining the observed moisture drainage patterns (related to transport), this may explain the connection to storm type mentioned by the reviewer. For example, the diurnal mountain–valley wind cycle and the South American Low-Level Jet (SALLJ), which drives MCS in southern Peru (Huang et al., 2024), may be key mechanisms leading to the region's G3 and G4 type drainage curves.

In summary, the reviewer is correct that all these mechanisms contribute to the climatological event of aerial rivers. In southern Chile, atmospheric rivers dominate the aerial river moisture transport, whereas in northern Chile and southern Peru, the dominant transport mechanisms may be mountain–valley wind cycle and the South American Low-Level Jet (SALLJ). While specific storm types play a significant role in moisture discharge, they are unlikely to be the primary factor shaping the moisture drainage curve and determining the resulting classification. While partitioning the contributions of individual mechanisms (e.g., atmospheric rivers, trade winds, the mountain–valley wind cycle, and the South American Low-Level Jet) is beyond the scope of the current study, we acknowledge its importance and leave it for future work. Nevertheless, we believe that incorporating these mechanisms into the discussion strengthens the connection between the patterns presented in Fig. 2 and well-recognized regional climate systems. We have included them where appropriate in the discussion (P13L6-L36) for readers' reference and we thank the reviewer for the insightful comments.

Rehbein, A. et al. (2019). Mesoscale convective systems over the Amazon basin: The GoAmazon2014/5 program. International Journal of Climatology.

Figure 3

I believe the box plots show the variability of the peaks. If so, the authors should

mention it in the caption. Also, peaks seem not to be always prominent, especially for G3. It is objective to always select a single peak, but the authors may need to ignore the second or third peaks even if they are very close to the first peak. I believe the treatment of the peaks may affect the distribution summarized in Figure 3a. The authors can provide a discussion on this issue if the authors think it is significant.

Figure 3. We thank the reviewer for this important point and have added clarification in the figure caption (P9L2L3). The reviewer is correct that some curves exhibit multiple peaks. Peaks were selected using a data-driven prominence level (0.05), according to the following criteria: (1) the number of peaks across all groups remains consistent within ±1% of this prominence, and (2) the first and second peaks on each curve are separated by at least 10% of the aggregated contribution. In our study, the turning point is defined as the location where moisture delivery efficiency declines (P4L5L6), our primary focus is therefore on the first peak. The second and third peaks may reflect transitions in the dominance of different long-term moisture transport mechanisms; however, this aspect is beyond the scope of this work. We agree that clarifying the prominence level used for peak selection improves the transparency of the paper, and we have now specified this in the Methods section (P22L17-L32). We thank the reviewer for the helpful reminder.

p4 Lines 8-25:

The authors discuss the results shown in Figure 3 based on the upwind basin size. Then why did the authors not summarize Figure 3 based on the upwind basin size, not the aggregated contribution? Isn't it more straightforward? Please explain/consider.

P4 L8-25 We thank the reviewer for the reminder. To enhance clarity, we have now included in Fig. 3 (P8 and P9L8L9) the median moisture drainage curve for each type (originally shown in Supplementary Fig. S6., now Fig. S5.), providing a reference for readers to better interpret the relationship between basin size and aggregated contribution for each type.

P7 Lines 5-6:

I believe showing the climatological wind pattern (maybe at 850hPa?) over the region would be highly effective for a better physical interpretation of the results. This applies to not only here.

We have added the long-term averaged 843 hPa wind field (2006–2015) to Fig. 2a (P6,

P6L9-P7L1) as suggested. We thank the reviewer for the recommendation.

P11 Lines 19-28:

I wonder whether the authors should discuss the ecological tipping point only based on the G1-G4 category. This should be strongly related to the vegetation types, including natural or human-managed. Although the point raised is important and worth mentioning, it is necessary to acknowledge such limitations for discussing the ecological tipping point.

P11 L19-L28 We thank the reviewer for highlighting the relevance of our point and agree that this implication should be presented with appropriate caution in the ongoing discussion of tipping points. Accordingly, we have revised the text to frame our discussion of the implications for ecological tipping points while explicitly noting these limitations (P19L14-P20L4, P20L11-L15).

P13 Line 6

As the authors mentioned, aerial rivers should be defined based on climatology.

With that said, the time period the authors focused on seems to be too short to define the climatology.

The authors need to quantify the impact of the selected time period on the results to enhance the robustness of the findings.

P13 L6 We thank the reviewer for the reminder. Indeed, continental-scale evaporation products have only become available in recent decades, limiting the full representation of aerial rivers within their climatological context. We have now acknowledged this limitation and added a discussion of the potential impacts of the selected time period on our results to enhance their contextualization (P24L22–L33, Supplementary D). We also thank the reviewer for this suggestion, which helps further strengthen the robustness of our findings. To address this, we examined the interannual variability of our results, showing modest interannual variability throughout the study period (P24L23-L24, Supplementary D, Fig. S7). We have also incorporated a discussion of our findings, considering the impacts of major regional climate variabilities, including ENSO, PDO, and AMO (P24L22-L33, Supplementary D, Fig. S7).

We thank the reviewer for the insightful comments, which have greatly strengthened the clarity and scientific quality of the manuscript. A point-by-point response to each comment is provided below, with reviewer comments in black and our responses in purple.

Reviewer #1 (Remarks to the Author):

The authors have submitted a revised version of their manuscript. In review #1, I suggested either "comprehensive revision and resubmission", or "rejection". The authors were given the opportunity to revise their submission. While the authors have made some changes, many of my original comments were not actually addressed. I recommend Rejection and provide these final comments.

We appreciate the reviewer's suggestions, which have helped us improve the manuscript. At the same time, At the same time, we note that Reviewer 1's comments appear to shift attention away from the manuscript's central contribution. The main achievement of this study is the objective identification of turning points and critical upwind basins, addressing a longstanding methodological challenge that remains unresolved in the literature. Building on this contribution, the manuscript also proposes a hydro-geographical framework for aerial river systems, distinguishing headwater, drainage, outfall, and plain regions, and thereby offering, to our knowledge, the first systematic conceptualization of invisible geomorphology within the aerial river regime.

We have substantially expanded the literature review in the revised manuscript, beyond the scope suggested by Reviewer 1 in the first round of review. Nevertheless, we believe that the second-round critique does not fully engage with the manuscript's principal methodological novelty and instead places primary emphasis on interpreting the manuscript as an application of existing moisture-recycling concepts to water management. This, however, is not presented as the central novelty of the study; rather, it is discussed only as one possible context in which our methodological results may interact with and inform existing research.

The main novelty of the manuscript lies in its methodological framework, as stated throughout the Abstract, Introduction, Results, and Discussion. In this light, we suggest that the reviewer's focus on a contextual bridging paragraph in the Discussion may understate the manuscript's principal methodological contribution and core results. At

the same time, we appreciate the reviewer's other scientific comments and have addressed them through additional clarifications, including the concern regarding the term "long-term."

1. The results are not long-term. The authors use the phrase "long-term" in the title, and throughout their text. However, 10 years of analysis is not consistent with a long-term analysis, let alone for the climate system. 10 years would be appropriate for shorter-term exploratory analyses. However, the authors are aiming to provide management-relevant science, and make mathematical claims about the behavior of atmospheric water transport. It would be challenging at best to provide insights relevant to water management with only 10 years of analysis. Multiple decades (at a minimum) is likely required to capture enough variability to support management-relevant findings.

In the title, "long-term" refers to the persistence of large-scale atmospheric moisture-flow patterns despite short-term variability at diurnal, synoptic, and seasonal timescales. Aerial rivers may evolve over time, like other climate patterns, yet still maintain a broadly consistent continental-scale structure over decades.

The choice of a 10-year period in this paper is informed by sub-period analyses based on moving windows of 1, 3, and 5 years, as well as by our preference for the more observation-based GLEAM-GPM data pair in this region. These results indicate that the 5-year window captures the evolving patterns in a more concentrated manner, suggesting that the 10-year pattern used in the main analysis is stable. We have added these analyses to help readers better assess the stability of the aerial river regime patterns (Supplementary E). For reference, we also provide results based on 18 years of the GLEAM-GPM pair (the longest period available for the dataset combination) as well as 30 years from the ERA5 reanalysis (1990-2019). The clustering results remain highly consistent across decades, differing by only about 5% in labeling over the continent (P19 L29-37, P20 L1-L9 and Supplementary E).

I will note that the Limitations section (which acknowledges the issue of a short duration) is at the end of Methods in this current revised manuscript. Nature Communications typically puts Methods at the end. So, in practice, the authors have put their Limitations section at the end of the manuscript, after all the other Methods. If the authors resubmit this work elsewhere, I suggest that they put such a limitations

section in the Discussion.

The authors follow what the journal instruction suggests.

2. Not the state of the art. Given that this is Nature Communications, it is reasonable to expect that cutting edge data and tools be used. While the authors use multiple datasets (including IMERG and GLEAM), they are also using ERA-Interim data for a period of 10 years. A cutting edge analysis would reflect the latest in atmospheric water tracking procedures. Given the authors use the WAM2-layers, that could mean using ERA5 reanalysis data (the current state-of-the-art reanalysis data product) for at least 30 years of analysis. The authors are aiming to publish their work in a highly prestigious journal, so I am deliberately holding this work to a high standard.

We thank the reviewer for this suggestion. In response, we chose the more observation-based dataset for the main analysis. For the reader's reference, we also include results based on 18 years (the longest time span available for the selected dataset) as well as 30 years from the ERA5 reanalysis. The clustering results remain highly consistent across decades, differing by only about 5% in labeling over the continent (P19 L29-37, P20 L1-L9 and Supplementary E).

3. *Insufficient engagement with the management literature. In the first set of comments, I suggested the authors needed to "much more substantially highlight how their work complements other approaches that have already been published", as well as "highlight where their suggestions diverge from or advance beyond past approaches." It is frustrating that the authors did not do this. The authors make claims about their own work and then reference previous studies. But in this revised manuscript the authors, again, fail to actually discuss how their work complements what has already been written over the past decade (at least) of atmospheric water management research.*

The reviewer's critique appears to reflect a misunderstanding of the manuscript's core novelty. Specifically, the reviewer interprets the study primarily as an application of existing moisture-recycling concepts to water management. However, the main contribution of this work is the objective identification of turning points and critical upwind basins, which addresses a longstanding methodological challenge that remains unresolved in the literature. This contribution is stated clearly throughout the Abstract,

Introduction, Results, and Discussion. We also explicitly discuss how our work complements existing studies across substantial portions of the Discussion (P10, L8–20 and L23–32; P11, L2–8; P14, L4–10 and L22–31; P15, L10 to P16 L14).

The paragraph on page 18, Line 3 of the revised manuscript is a perfect example of inadequate claims of novelty. The second sentence of this paragraph reads: "Managing aerial rivers at the continental scale necessitates a reconceptualization of the governable dimensions of hydrology and a redefinition of the critical zones where water resource conservation intersects with land-use decisions."
This is written as an original thought (and it very well may be). However, the same concept has been written about many times by others over the past ten years at least. Currently, a reader of the author's Revised manuscript could easily draw the conclusion that very little if anything has been written about these subjects, while the opposite is true. Indeed, much has been written about atmospheric water management, precipitation recycling management, upwind source region management, and even aerial river management. The authors cite some of this work, but they did not "substantially highlight how their work complements other approaches that have already been published", and they did not "highlight where their suggestions diverge from or advance beyond past approaches."

The central contribution of this study is the objective identification of turning points and critical upwind basins, addressing a longstanding methodological challenge that has not yet been resolved in the existing literature. Building on this approach, the manuscript further introduces a hydro-geographical framework for aerial river systems, distinguishing headwater, drainage, outfall, and plain regions. It represents the first systematic conceptualization of invisible geomorphology within the aerial river regime.

We would like to clarify that the revised manuscript includes a substantially expanded literature review, exceeding the scope suggested in Reviewer 1's first-round comments. Reviewer 1 claims in the second-round critique that additional literature should be cited in relation to our main contribution, but fail to reference studies demonstrating the objective identification of critical upwind basins using mathematical methods are referenced. This capability constitutes the core novelty of this study. The reviewer's concern therefore appears to arise from a misinterpretation of the manuscript's primary

contribution.

The reviewer interprets the manuscript primarily as an application of existing concepts related to moisture recycling for water management, neither of which is claimed as novel in this study. Our novelty has been clearly described in the Abstract, Introduction, Results, and Discussion sections.

This interpretation is reflected in the reviewer's quotation of a bridging paragraph on page 18 (situation at the seventh page of the Discussion) as evidence of a lack of novelty, although the paragraph was intended only to contextualize the methodological findings rather than present the study's core contribution. The critique shifts focus away from the manuscript's central methodological framework, including the primary results and the preceding sections of the Discussion where the main contributions are presented.

Reviewer #2 (Remarks to the Author):

I believe that the authors have responded to my comments and suggestions in a satisfactory way. Therefore, I suggest acceptance of the paper.

Reviewer #2 (Remarks on code availability):

I believe that the authors have responded to my comments and suggestions in a satisfactory way. Therefore, I suggest acceptance of the paper.

We appreciate the reviewer's suggestions, which have helped strengthen the manuscript, and we thank the reviewer for recommending its acceptance.

We thank the reviewer for the insightful comments, which have greatly strengthened the clarity and scientific quality of the manuscript. A point-by-point response to each comment is provided below, with reviewer comments in black and our responses in purple.

Reviewer #3 (Remarks to the Author):

I would like to thank the authors for their thoughtful and comprehensive responses. The manuscript has certainly improved, particularly in terms of clarity and structure. That said, I believe there are still a few areas that would benefit from further clarification or development, especially to strengthen the scientific foundation and practical applicability of the study, which are outlined below:

We thank the reviewer for the kind feedback and here we provide the clarification of the parts that the reviewer suggested. This has helped fortify the scientific foundation and applicability of our study.

Methodological Uncertainty
I appreciate the authors' efforts in conducting a bootstrap analysis for quantifying the uncertainty. It is now clear that the selection of four clusters was not significantly optimal, since five or six clusters show similar performance. Based on these results, I believe the authors need a stronger justification for focusing only on the four-cluster results.
Can the authors explain the similarity of cluster divisions in the cases of four, five, and six clusters based on physical mechanisms, such as climatological moisture flow patterns? Such additional analysis is necessary to increase the robustness of the findings presented in the paper based on the four-cluster results.

The clustering results for four, five, and six clusters show a broadly consistent group structure. Increasing the number of clusters mainly results in the subdivision of existing clusters rather than the emergence of entirely new patterns. In particular, the additional clusters in the five- and six-cluster solutions largely represent finer partitioning of groups already identified in the four-cluster configuration. A subdivision within G2 we

have identified in the main text (G2 Atmospheric drainage and Supplementary S4) leads to the five-cluster and six-cluster solutions. In the six-cluster case, an additional subdivision appears in G3, associated with differences in the local recycling ratio. This G3 subgroup exhibits a lower local recycling contribution, and its moisture drainage curve departs from the x-axis earlier, with a steeper slope. However, because this study focuses on the moisture delivery function of aerial rivers, both subdivisions of G3 show similar behavior of the outfall region where wind speed slows abruptly. Therefore, we adopt the four-cluster solution, which better represents the primary structure relevant to aerial river delivery dynamics. Studies on local rainfall partition may benefit from exploring these finer subdivisions of the underlying mechanisms.

We have added this discussion to Supplementary F Fig. S14, which is cited in the main text (P20, L17-18)

Potential Trade-offs in the Framework

I appreciate the addition of discussion around the potential downstream impacts of interventions like reforestation. However, the current framing still feels somewhat conceptual, and readers may find it difficult to evaluate how this framework might perform in a real-world context.

Would it be possible to include a simple illustrative example showing how trade-offs between upstream and downstream regions could be assessed using your framework? This doesn't have to be a full model—just a basic water balance sketch or reference to existing studies could help ground the discussion.

We thank the reviewer for the suggestion to enhance the applicability of our study. In response, we have added a section in Supplementary C presenting an example framework for assessing downstream runoff impacts from strategic reforestation in a critical upwind basin. This section is referenced in the main text (P11, L21–22).

Time Period and Climatological Representation

It would be helpful to discuss more explicitly how this limitation might affect the results. For example, do the classifications hold steady across different sub-periods? If longer datasets (e.g., reanalysis products) suggest similar patterns, even a mention of that would help put the current results into context.

We thank the reviewer for this valuable suggestion, which has helped us further

strengthen the scientific basis of the manuscript. In response, we have expanded the discussion in the main text (P19 L29-37, P20 L1-L9) and added supplementary analyses (Supplementary E) assessing the choice of sub-period lengths (1, 3, and 5 years) and longer-period analysis. The results indicate that a 3-year window is sufficient to smooth much of the annual variability, whereas a 5-year window captures the principal patterns in a clear and concentrated manner. For longer-period tests, the clustering patterns are in strong agreement with those derived from the 18-year GLEAM–GPM record (2001–2018), the longest continuous period available without data gaps, with only about a 5% difference in grouping across the continent. We further compared these results with those obtained using ERA5 reanalysis data over 10-year (2006–2015) and 30-year (1990–2019) periods, and found the clustering structure to be similarly robust, again with only about a 5% difference. This additional comparison has been included for the reader's reference. It should be noted that the ERA5-based experiment was included primarily to assess the influence of longer-term climate variability, whereas the GLEAM–GPM dataset was retained for the main analysis because of its more observation-based character. Overall, these additional results provide further support for the robustness of the methodological choices adopted in this study.

Peak Selection

The added explanation of peak selection criteria is helpful. However, since some curves are multi-peaked, I wonder how sensitive the analysis is to this prominence threshold. Would varying the threshold (e.g., from 0.05 to 0.03) significantly change the distribution or classification?

The classification results are robust to the choice of prominence threshold in terms of grid-cell counts, spatial patterns, and the reported interquartile ranges for each group. Variation is mainly observed in the distribution of outliers, while the overall structure remains stable. For completeness, we include the box plot based on a prominence threshold of 0.03 for the reviewers' reference.

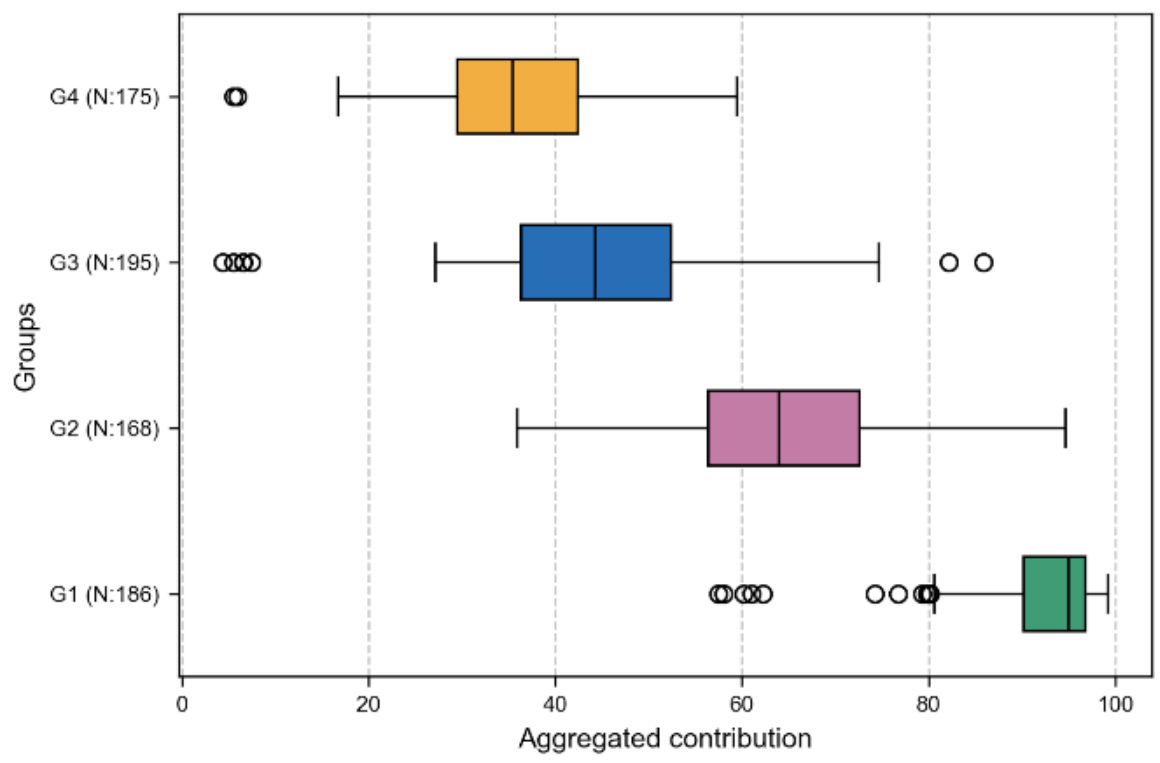
G4 (N:175)
G3 (N:195)
G2 (N:168)
G1 (N:186)
Groups
0
20
40
60
80
100
Aggregated contribution

We thank the reviewer for the insightful comments, which have greatly strengthened the clarity and scientific quality of the manuscript. A point-by-point response to each comment is provided below, with reviewer comments in black and our responses in purple.

Reviewer #1 (Remarks to the Author):

The authors have made many new additions to their analysis to respond to previous reviewer comments, something that is very helpful toward evaluating the robustness of the different clusters, and ultimately for evaluating whether the claims are supportable related to the robustness of the different types of upwind aerial basins. This is good progress. I have a final revision request that I describe below. I also want to reiterate that I am holding the authors to a high standard here because this is for Nature Communications.

We thank the reviewer for constructive comments which help to strengthen the manuscript.

The authors suggest that their contribution is centrally methodological, and their statements throughout their work (including the first two sentences of their final Maintext paragraph) point to the fact that they reveal positions that reflect long-term planetary moisture transport:

"The moisture drainage types we found here are geographic divisions shaped by long-term planetary atmospheric moisture transport which is relevant for regional water resources. They correspond to fluvial positions within the continental aerial river system: headwater, drainage, outfall, and plain."

This is not me shifting the focus away from the author's intended contribution; The authors state their intended contribution very clearly in this final paragraph.

We agree with the reviewer that our contribution is not purely methodological. As the reviewer noted, the main contribution of this study lies not in describing the well-established process of moisture transport, but in identifying distinct moisture drainage types and the invisible geographies they shape.

That being said, I think there is an easy/straightforward resolution to all of this: Provide clearer more comprehensive information about the long-term WAM2layers analysis the authors performed using ERA5.

The authors note that they have already completed this 30 yr analysis, but only show a single figure in the Supplement (Supp Fig S11). They provide almost no other detail about this (considerable) additional work. 30 years of ERA5 analysis run through the WAM2layers is a substantial undertaking, and there is much that could be done with this analysis to support their claim of long-term relevance.

I would like to see a full suite of sensitivity analysis figures to complement the central findings of the GLEAM-GPM data. Specifically, provide the following for the 30 year ERA5 WAM2layers runs (which you've already produced for the GLEAM-GPM analysis):

1. Maintext Fig 2b, 3a and 3b, Table 1, and

2. Supplemental Figs S3, S5, and S7.

These could all go in the Supplement (which doesn't cost anything in word count, yet will build much more trust with a skeptical reader).

I would recommend (though its up to the authors) to include a comparison figure in the maintext which would give the average reader of the article (who doesn't dive into the supplement) a chance to see the good agreement between the long-term dataset (ERA5) and the GPM-GLEAM dataset the authors choose to feature.

We appreciate the reviewer's suggestion. However, substantial differences, reaching up to 90%, are already present between the ERA5 and GPM–GLEAM products in their underlying precipitation and evapotranspiration fields before our methodology is applied (see Figure 1 below). Requiring the results from the ERA5 and GPM–GLEAM dataset to quantitatively align would conflate dataset uncertainty with methodological consistency.

To sum up: The authors have are almost there. The final step is to show comprehensively that their 10 year results (from GPM-GLEAM) align closely with the 30-year results that are actually at the time scale of long-term atmospheric behavior.

Making these figures should be a small amount of work for the authors since they have already completed the ERA5-based WAM2layers analysis. And yet, these figures will disproportionately strengthen their work by providing the fundamental evidence that their concluding paragraph claims to find.

If the authors do this, I will be happy to see this contribution published.

We thank the reviewer for this important comment. We agree that presenting the ERA5

results in only a single figure did not sufficiently document the additional analysis. We have therefore added the suggested figures to Supplementary H, provided a brief description of the analysis configuration, and clarified the specific role of ERA5 in the study. As ERA5 is a reanalysis product, these analyses are included as a supplementary reference for assessing long-term temporal variability and should not be interpreted as observational evidence.

ERA5 and GPM–GLEAM differ substantially in their data sources, structures, and estimation approaches. To illustrate these differences, we present below the original precipitation and evaporation fields from both datasets over the same analysis period. The comparison shows that discrepancies are already present in the input fields before our methodology is applied. Quantitative agreement between the ERA5 and GPM–GLEAM-based results should therefore not serve as the criterion for assessing methodological consistency.

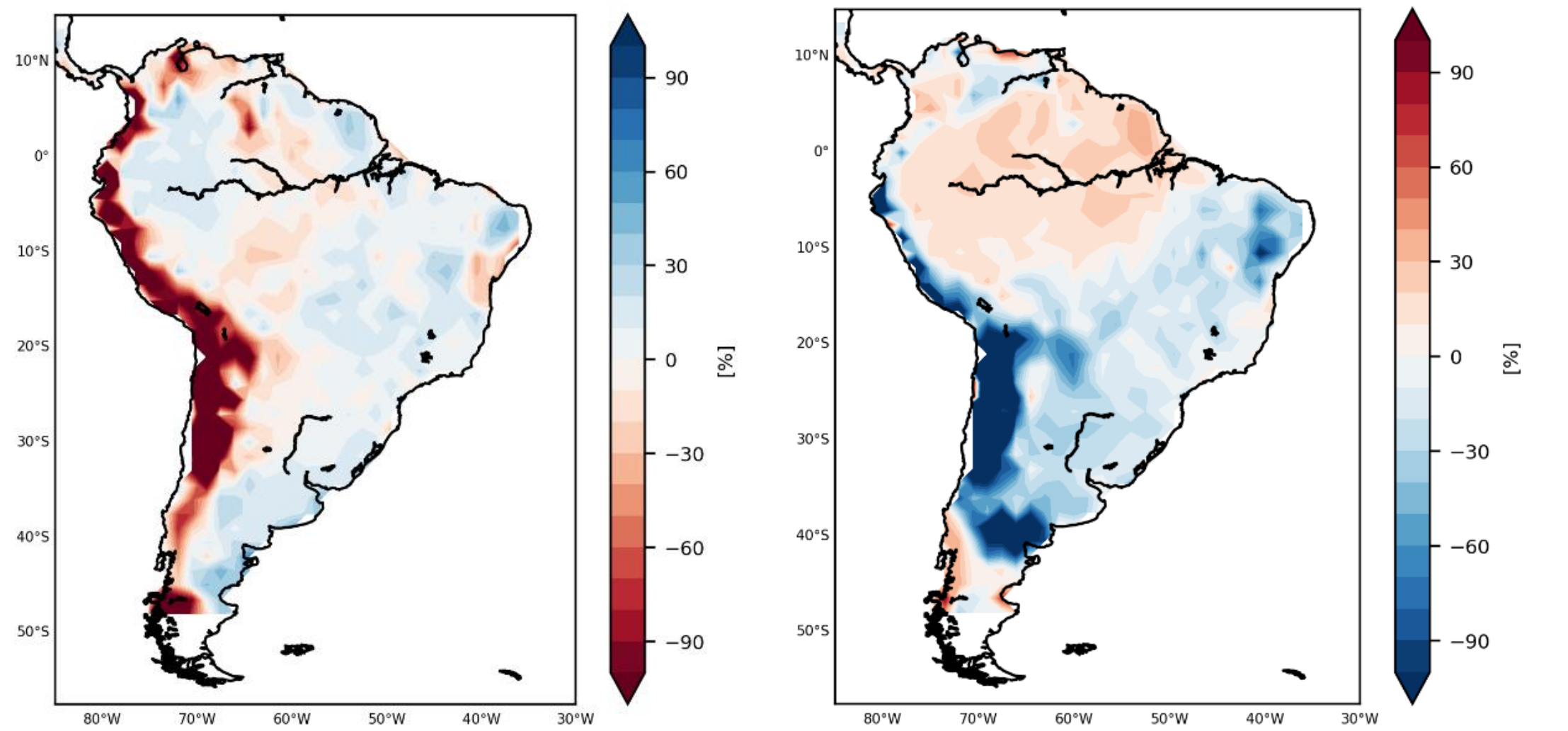

Fig 1. Normalized differences between GPM–GLEAM and ERA5 for 2006–2015, using GPM–GLEAM as the reference: precipitation (left) and evaporation (right), showing substantial differences of up to 90% before our methodology is applied.

Although ERA5 provides globally consistent and spatially detailed reanalysis fields, it is a model-based product generated by combining numerical weather prediction with data assimilation. It should not be interpreted in the same manner as direct observational data. Lavers et al. (2022) documented substantial ERA5 precipitation errors in tropical regions. Over the Amazon Basin, Polasky et al. (2025) identified discrepancies between ERA5 and observational datasets, particularly in the southern and western regions.

They found that ERA5 produces a drying trend in the southern Amazon that is not supported by ground-based or satellite observations and suggested that these uncertainties may arise from the representation of moisture transport within the reanalysis system. Baker et al. (2021) further showed that precipitation biases in ERA5 can propagate into its regional evapotranspiration estimates.

For these reasons, GPM–GLEAM, as the more observation-based dataset, remains the primary basis for defining the main aerial river drainage characteristics. ERA5, which captures broad, long-term climatic patterns, is used as an additional reference for assessing longer-term temporal variability because it provides a continuous 30-year record without data gaps. In response to the reviewer's previous comment, we evaluated temporal-window sensitivity separately within each dataset: the original 10-year GPM–GLEAM results were compared with the longest continuous 18-year GPM–GLEAM record available without data gaps, while the 10-year ERA5 results were compared with the corresponding 30-year ERA5 analysis.

These comparisons show that the principal aerial river drainage structures remain relatively stable across longer temporal windows. Specifically, the 10-year and 30-year ERA5 analyses yielded different classification labels for only approximately 5% of grid cells across the continent. Similarly, differences between the 10-year and 18-year GPM–GLEAM analyses were smaller than those arising from the use of different datasets. These results suggest that dataset uncertainty exceeds temporal-window uncertainty in this case. We have revised the corresponding discussion on P20L16–L24, to clarify this distinction.

Overall, these results support our conclusion that the principal aerial river drainage structures are robust across longer temporal windows, whereas larger discrepancies arise from differences between datasets rather than from the length of the analysis period itself. In the revised version, we have retained GPM–GLEAM as the basis of the primary analysis and used ERA5 as a supplementary assessment of temporal sensitivity.

We have added the ERA5-related figures suggested by the reviewer to Supplementary H, clarified their purpose, and included a footnote stating that the ERA5-based comparison should not be interpreted as observational evidence. These revisions improve transparency while maintaining the focus of the manuscript on the primary observation-based dataset.

References:

Baker, J. C. A et al. (2021). Evapotranspiration in the Amazon: Spatial patterns, seasonality, and recent trends in observations, reanalysis, and climate models. Hydrology and Earth System Sciences, 25, 2279–2300. https://doi.org/10.5194/hess-25-2279-2021

Lavers, D. A. et al. (2022). An evaluation of ERA5 precipitation for climate monitoring. Quarterly Journal of the Royal Meteorological Society, 148, 3152–3165. https://doi.org/10.1002/qj.4351

Polasky, A. et al. (2025). Discrepancies in precipitation trends between observational and reanalysis datasets in the Amazon Basin. Scientific Reports, 15, Article 7268. https://doi.org/10.1038/s41598-025-87418-5

We thank the reviewer for the insightful comments, which have greatly strengthened the clarity and scientific quality of the manuscript. A point-by-point response to each comment is provided below, with reviewer comments in black and our responses in purple.

Reviewer #3 (Remarks to the Author):

This manuscript addresses a critical methodological challenge in water management: how to define upwind moisture source regions objectively without relying on arbitrary fixed thresholds. The core concept of critical upwind basins is originally proposed by the authors and has significant potential to improve land-water management and moisture governance under a changing climate.

We thank the reviewer for the constructive comments, which helped us clarify the methodology and implications of our work, and we appreciate the reviewer's recognition of the originality of our study.

However, I have the following major concerns remaining:

#1

In Table 1 and the Methods section, the authors define "Moisture transport efficiency" by dividing the total moisture content by the distance traveled. However, physically, this represents a spatial derivative or a path-averaged line density, not an "efficiency" in the standard hydrological sense (which is usually a dimensionless ratio of output to input, or a measure of mass loss). The authors should clarify why this specific metric represents efficiency, or rename the variable to better match its physical meaning.

We thank the reviewer for pointing this out. We agree that the previous terminology did not fully reflect the physical character of the variable. We have therefore revised the term to "delivery intensity," which more accurately captures its physical meaning.

To distinguish between the two related concepts, we now define "delivery intensity" as an aerial river characteristic representing the path-averaged linear density of moisture transport. By contrast, the upwind moisture contribution per unit area used to identify turning points along the drainage curve is now referred to as "upwind moisture

contribution intensity." This distinction is explained on P7L2-L4, and the terminology has been revised throughout the manuscript to improve conceptual clarity and consistency.

We have also revised the description of water content in response to the reviewer's Suggestion #2. To facilitate comparison with other hydrological quantities while avoiding dependence on grid-cell size, we now report water content for each group as an annual precipitation depth in mm. Accordingly, delivery intensity is expressed in mm/km. We have revised the Methods section and Table 1 to clarify the definition, calculation, units, and physical interpretation of these variables (P7L2-L4, P7L15, P19L9-L16). Interestingly, following this revision, we found that the annual water content associated with continental aerial rivers, calculated by multiplying the depth recorded for each grid cell by that grid cell's area and then summing across the continent, is comparable in magnitude to the annual volume of water discharged by South American surface rivers to the ocean, approximately 1.1×10^{13} m^3/year.

We thank the reviewer for this valuable suggestion, which has improved the scientific clarity of the results and enabled a more direct comparison with other hydrological characteristics.

#2

Methods state that moisture content is calculated as the sum of the moisture load along the pathway. If this is a simple summation of values sampled at 3-hour intervals, the total value will artificially change depending on the sampling frequency and resolution. The authors need to clarify the exact mathematical formulation (e.g., whether it is a path integral) and prove that the metric is independent of model resolution.

We thank the reviewer for this insightful comment regarding the scaling and consistency of our tracking metrics.

Water content is calculated from outputs of the WAM-2layers moisture-tracking framework, which quantifies the volume of moisture delivered from an upwind source grid cell to a downwind target grid cell. By backtracking and integrating these contributions across the unique spatial grid cells that constitute the aerial river pathway, as identified using the simplified Lagrangian point-tracking algorithm, the resulting

sum represents the cumulative terrestrial precipitation delivered to the target location. Because the backtracking procedure counts unique spatial grid cells rather than repeatedly accumulating contributions over discrete time steps, it avoids double-counting; consequently, the choice of temporal resolution (dt) does not affect the tracking results. We agree that this configuration was not explained sufficiently clearly in the previous version and have revised accordingly (P19L9-13, Supplementary E).

In the previous version, however, the integrated water content reported in Table 1 was expressed as a water volume averaged per grid cell. We agree with the reviewer that this original metric was sensitive to spatial grid-cell size, particularly when comparing results across different spatial grids. To address this issue, we have revised Table 1 to report water content as a normalized precipitation depth in millimetres (mm). Expressing the metric as the depth of precipitation delivered through the aerial river pathway removes the grid-cell area multiplier and makes it more suitable for comparison across spatial configurations.

Under the revised formulation, the water-content values of G3 and G4 are no longer significantly different. This change does not alter the main argument, which was not based on differences in water content among the drainage types. Rather, the revised metric provides a clearer representation of the characteristic moisture load reacing each aerial river drainage type by removing the influence of grid cell area. We have updated Table 1 (P7L15), revised the corresponding text in the Methods section (P19L9-L13), and added an explicit explanation of the mathematical formulation and physical interpretation of this bounded metric in Supplementary E.

#3

In the Discussion section, the authors argue that deforestation per unit area in the upwind basins of G1 and G2 will trigger Amazonian forest dieback more easily than in G3 or G4. They base this claim entirely on the high delivery efficiency per unit area shown by the sharpness of the G1/G2 drainage curves.

However, this conclusion overlooks the absolute moisture dependency of the receiving regions: According to Table 1, G1 regions rely almost entirely on ocean sources, and continental-sourced rainfall accounts for only 12% of their total precipitation. Thus, even if its critical upwind basin is completely deforested, the maximum potential loss

of total rainfall in G1 is limited to about 11%. In contrast, G4 regions depend heavily on continental land sources, with a continental recycling ratio of 49%.

While G1 and G2 upwind basins show higher sensitivity per unit area, G4 is far more vulnerable in terms of the absolute water volume at risk. The claim that the tipping point threshold is lower for G1/G2 clearing is a logical leap. The authors must revise the discussion to balance per-area sensitivity with absolute hydrological vulnerability.

The reviewer is correct that this argument should also account for dependence on terrestrially sourced rainfall. We agree and have revised the Discussion accordingly. In the revised text, we distinguish between delivery intensity and moisture vulnerability per unit area of deforestation within the critical upwind basin. These revisions are presented on P15L10-L29, and P16L1-L4. We thank the reviewer for this valuable suggestion.

#4

Under "Deriving moisture drainage curves" in the Methods, the authors state that the drainage curves were transformed to natural logarithms, interpolated into 1,000 evenly spaced intervals, and then converted back to the original scale using an exponential function before calculating the local curve angles and derivatives.

Transforming a highly non-linear, exponential-like curve into log-space, performing interpolation, and then transforming it back via an exponential function can mathematically alter the local curvature (the second derivative).

This pre-processing step might introduce artificial smoothing or sharpening at the critical bend of the curve. The authors need to demonstrate whether the identified turning points change significantly if the interpolation and derivative calculations are performed directly on the raw, non-log-transformed data.

We appreciate the reviewer's comments regarding the mathematical implications of our pre-processing pipeline. This step was introduced to address specific characteristics of the raw tracking outputs, which contain high-frequency, small-scale spatial variability and step-like discontinuities associated with the discrete model grid. We therefore applied a physically informed smoothing procedure to the moisture drainage curve to reduce grid-scale artefacts while preserving the dominant structure required for turning-point identification.

Here, we clarify the physical and mathematical rationale behind this design choice. We

have also clarified this rationale and documented the differences between the results obtained from the raw and preprocessed curves in the revised version.

1. Mathematical justification of the log-transformed preprocessing

The pre-processing pipeline consists of three steps applied to the raw moisture drainage curves generated by the WAM-2layers model. First, we convert the raw moisture drainage curve into a cumulative distribution function (CDF) and take its natural logarithm. Second, we apply linear interpolation to the log-transformed CDF. Third, we transform the interpolated curve back to the original scale using the exponential function and calculate its derivative to obtain the corresponding probability density function (PDF) and local curve angles.

The rationale for applying interpolation in log-CDF space is that moisture accumulation along an aerial river pathway is typically highly skewed, with rapid accumulation in dominant contributing areas and a long tail of weaker contributions. In this context, interpolation in the original scale would place disproportionate weight on large absolute values, whereas interpolation in log-CDF space preserves the relative structure of both high- and low-contribution segments. This allows the dominant macroscopic drainage pattern to be retained while reducing localized, high-frequency numerical variability associated with the discrete model grid. Mathematically, an approximately linear relationship in log-CDF space implies that cumulative moisture accumulation exhibits exponential- or Pareto-like scaling. The corresponding PDF, obtained by differentiation, retains a lower-order, smoothly varying structure consistent with the statistical-mechanical behavior of large-scale atmospheric diffusion and advection. The procedure was applied to suppress localized, high-frequency variability while preserving the macroscopic climatological behavior of aerial river basins.

2.Comparison of turning-point identification using preprocessed and raw data approaches

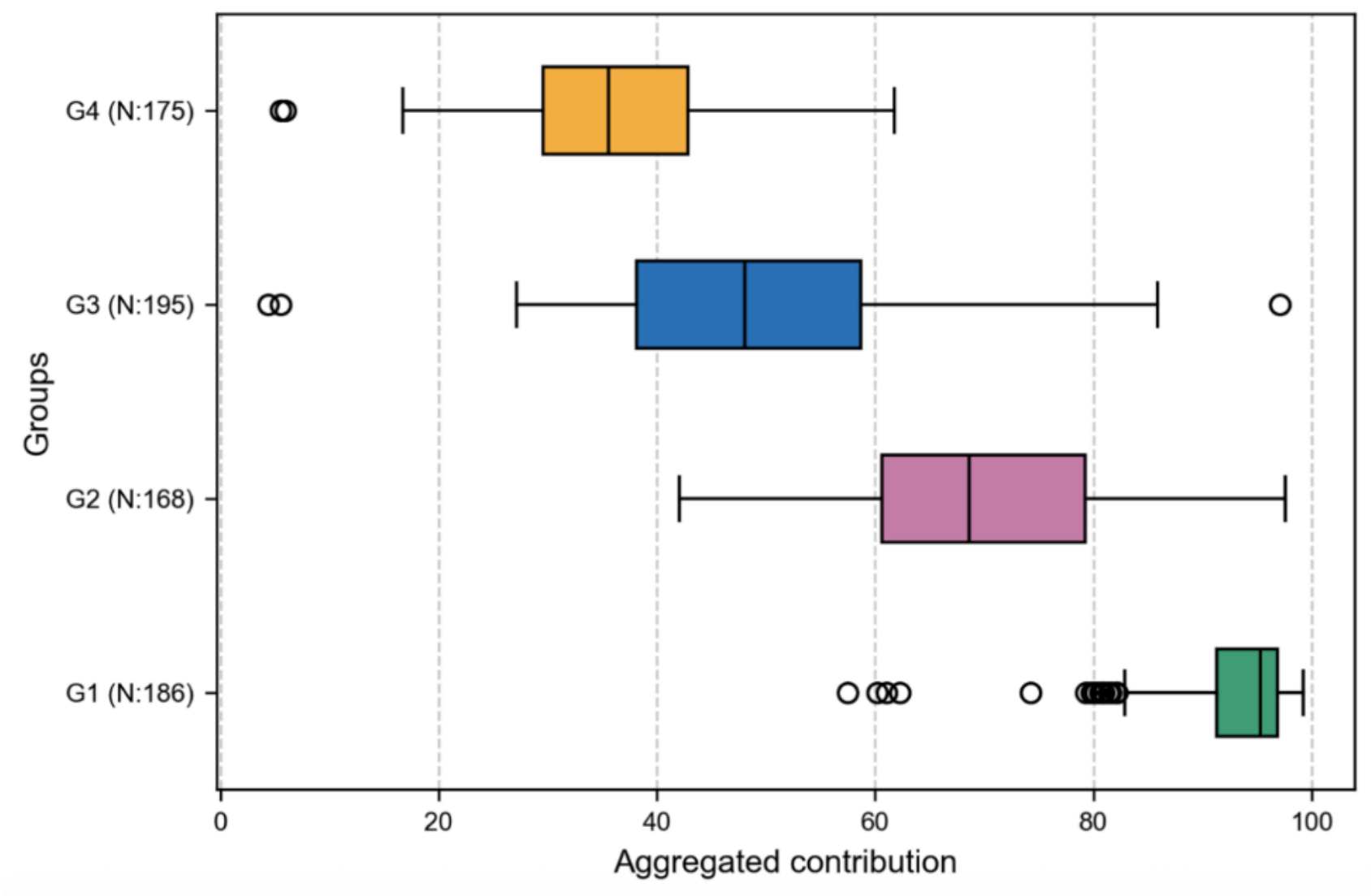

Fig 1. Boxplots of turning points derived from the log-transformed preprocessed data

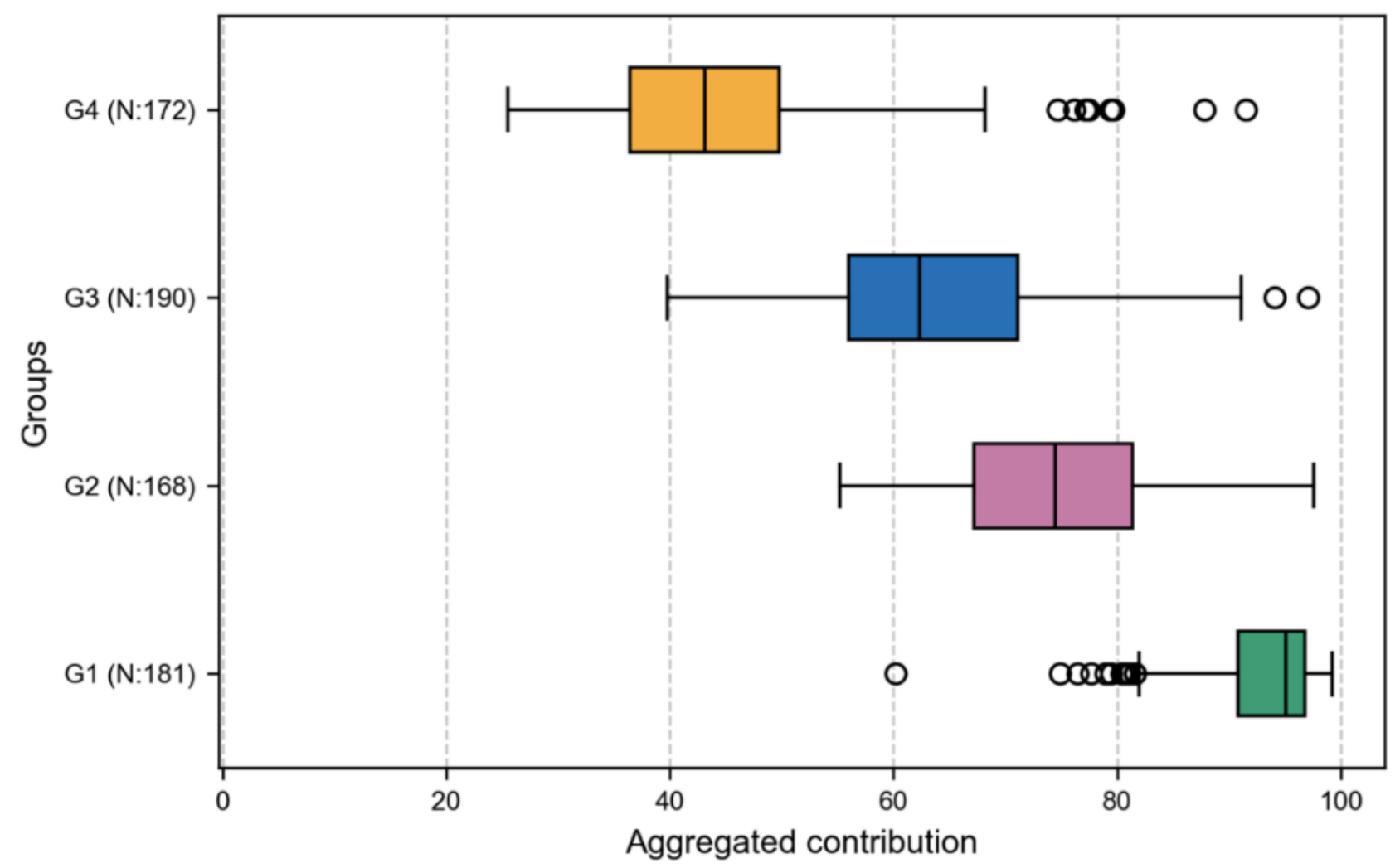

Fig 2. Boxplots of turning points derived from the raw data

We repeated the full turning-point identification analysis using the raw, non-log-transformed moisture drainage data. The turning points derived from the preprocessed curves (Fig. 1) were then compared with those obtained directly from the raw data (Fig. 2), as shown above.

The two approaches are generally consistent in their overall conclusions, with minor differences in the tails and outliers. The group size of each drainage type is similar between the two approaches, with differences of less than 3%, indicating that the classification is stable with respect to the preprocessing procedure. The preprocessed approach produces slightly lower median values for G3 and G4 and show changes in

some extreme values. Nevertheless, the interquartile ranges and relative separation among groups remain broadly comparable. These results indicate that the methodological choice preserves the dominant group structure and relative contribution patterns.

The most noticeable difference occurs in G3, which is characterized by more complex terrain and transitional moisture-delivery regimes and may therefore be more sensitive to local-scale variability in the raw tracking outputs. The complexity in moisture-delivery behavior within G3 provides an interesting avenue for future research.

Nevertheless, although calculating angle changes directly from the raw data is technically straightforward, we note that using raw, non-log-transformed curves for turning-point detection introduces additional mathematical uncertainty. The raw tracking outputs contain high-frequency, small-scale spatial features and step-like discontinuities associated with the discrete model grid. Applying angle-change analysis to these raw fields amplifies such variability, producing multiple artificial local peaks that obscure the identification of the macroscopic turning point. This sensitivity is reflected in the missing value in the raw-data analysis (Fig 2). To address this issue, we applied a physically informed smoothing procedure to the moisture drainage curve to suppress grid-scale artefacts while retaining the large-scale structure relevant for turning-point identification.

To enhance transparency, we have included an explanation of the preprocessing rationale, the comparison, and the associated discussion in Supplementary D, and have explicitly referenced this in the Methods section (P17L27L28).

Reviewer #1 (Remarks to the Author):

Thank you. The authors have responded to all of my comments.

We appreciate the reviewer’s constructive suggestions, which have helped us strengthen the manuscript.

Reviewer #3 (Remarks to the Author):

I appreciate the authors addressing my comments, which I beleive significantly improved the quality of the paper.

We thank the reviewer for the constructive comments, which have helped improve the clarity and quality of the manuscript.
